# Loss of ganglioglomerular nerve input to the carotid body impacts the hypoxic ventilatory response in freely-moving rats

**DOI:** 10.3389/fphys.2023.1007043

**Published:** 2023-03-16

**Authors:** Paulina M. Getsy, Gregory A. Coffee, Stephen J. Lewis

**Affiliations:** ^1^ Department of Pediatrics, Division of Pulmonology, Allergy and Immunology, Case Western Reserve University, Cleveland, OH, United States; ^2^ Department of Pharmacology, Case Western Reserve University, Cleveland, OH, United States; ^3^ Functional Electrical Stimulation Center, Case Western Reserve University, Cleveland, OH, United States

**Keywords:** carotid body, ganglioglomerular nerve, ventilatory parameters, hypoxic challenge, juvenile rats

## Abstract

The carotid bodies are the primary sensors of blood pH, pO_2_ and pCO_2_. The ganglioglomerular nerve (GGN) provides post-ganglionic sympathetic nerve input to the carotid bodies, however the physiological relevance of this innervation is still unclear. The main objective of this study was to determine how the absence of the GGN influences the hypoxic ventilatory response in juvenile rats. As such, we determined the ventilatory responses that occur during and following five successive episodes of hypoxic gas challenge (HXC, 10% O_2_, 90% N_2_), each separated by 15 min of room-air, in juvenile (P25) sham-operated (SHAM) male Sprague Dawley rats and in those with bilateral transection of the ganglioglomerular nerves (GGNX). The key findings were that 1) resting ventilatory parameters were similar in SHAM and GGNX rats, 2) the initial changes in frequency of breathing, tidal volume, minute ventilation, inspiratory time, peak inspiratory and expiratory flows, and inspiratory and expiratory drives were markedly different in GGNX rats, 3) the initial changes in expiratory time, relaxation time, end inspiratory or expiratory pauses, apneic pause and non-eupneic breathing index (NEBI) were similar in SHAM and GGNX rats, 4) the plateau phases obtained during each HXC were similar in SHAM and GGNX rats, and 5) the ventilatory responses that occurred upon return to room-air were similar in SHAM and GGNX rats. Overall, these changes in ventilation during and following HXC in GGNX rats raises the possibility the loss of GGN input to the carotid bodies effects how primary glomus cells respond to hypoxia and the return to room-air.

## Highlights

• Bilateral GGNX blunts the initial increases in minute ventilation in response to consecutive hypoxic challenges in juvenile Sprague Dawley rats.
• Bilateral GGNX blunts the initial increases in inspiratory and expiratory drives in response to consecutive hypoxic challenges.
• Bilateral GGNX blunts the initial increases in non-eupneic breathing index that occur upon return to room-air following consecutive hypoxic challenges.

## Introduction

The superior cervical ganglion (SCG) contains cell bodies of post-ganglionic sympathetic nerves (Rando et al., 1981; Tang et al., 1995a; Tang et al., 1995b; Tang et al., 1995c; Llewellyn-Smith et al., 1998) and small intensely fluorescent (SIF) cells (McDonald, 1983a; McDonald, 1983b; Zaidi and Matthews, 2013; Takaki et al., 2015). These post-ganglionic sympathetic nerves and SIF cells receive their pre-ganglionic innervation from thoracic spinal cord (T1-T4) nerves that course through the ipsilateral cervical sympathetic chain (CSC) (Rando et al., 1981; McDonald, 1983a; McDonald, 1983b; Tang et al., 1995a; Tang et al., 1995b; Tang et al., 1995c; Llewellyn-Smith et al., 1998). Previously we reported that freely-moving mice with bilateral CSC transection (CSCX) (Getsy et al., 2021a) or bilateral superior cervical ganglionectomy (SCGX) (Getsy et al., 2021b) display substantially different ventilatory responses during and after a hypoxic gas challenge (HXC) than those of their sham-operated counterparts. While providing evidence that the CSC-SCG complex has a role in modulating the hypoxic ventilatory response (HVR), the exact pathways and target structures by which this complex regulates the HVR were not elucidated in these mouse studies. It is well-known that, post-ganglionic nerves in the SCG project to target structures in the head and neck *via* the external (ECN) and the internal (ICN) carotid nerves (Bowers and Zigmond, 1979; Buller and Bolter, 1997; Asamoto, 2004; Savastano et al., 2010) including, the upper airway and tongue (Flett and Bell, 1991; Kummer et al., 1992; O'Halloran et al., 1996; O'Halloran et al., 1998; Hisa et al., 1999; Wang and Chiou, 2004; Oh et al., 2006), vascular structures within the brain including the Circle of Willis and cerebral arteries (Sadoshima et al., 1981; Sadoshima et al., 1983a; Sadoshima et al., 1983b; Werber and Heistad, 1984), and nuclei in the hypothalamus and brainstem (Cardinali et al., 1981a; Cardinali et al., 1981b; Cardinali et al., 1982; Gallardo et al., 1984; Saavedra, 1985; Wiberg and Widenfalk, 1993; Westerhaus and Loewy, 1999; Esquifino et al., 2004; Hughes-Davis et al., 2005; Mathew, 2007).

SCG post-ganglionic neurons in the ECN branch into the ganglioglomerular nerve (GGN) to innervate type 1 glomus (chemoresponsive) cells, chemoreceptor afferent nerve terminals and vasculature in the carotid bodies (Biscoe and Purves, 1967; Zapata et al., 1969; Bowers and Zigmond, 1979; Brattström, 1981a; McDonald and Mitchell, 1981; McDonald, 1983a; McDonald, 1983b; Verna et al., 1984; Torrealba and Claps, 1988; Ichikawa, 2002; Asamoto, 2004; Savastano et al., 2010). Fibers in the GGN also modulate responsiveness of baroreceptor afferent nerve terminals within the carotid sinus (Floyd and Neil, 1952; Rees, 1967; Bolter and Ledsome, 1976; Brattström, 1981b; Felder et al., 1983; Buller and Bolter, 1993). It is clear that the ability of the GGN input to elicit vasoconstriction within the carotid body indirectly activates primary glomus cells *via* ensuing tissue hypoxemia (Majcherczyk et al., 1974; Llados and Zapata, 1978; Majcherczyk et al., 1980; Matsumoto et al., 1981; Yokoyama et al., 2015). However, studies examining the direct effects of GGN input to the carotid body on resting activity of glomus cells and chemoafferents in the carotid sinus nerve (CSN), and the responses of these structures during HXC have yielded controversial findings. For instance, GGN (and CSC) activity increases during HXC raising the likelihood that excitatory neurotransmitters, such as norepinephrine, dopamine and neuropeptide Y are released during the challenge (Lahiri et al., 1986; Matsumoto et al., 1986; Matsumoto et al., 1987; Yokoyama et al., 2015), although direct activation of the GGN decreases the chemosensory responsiveness to HXC in the carotid bodies of the cat (McQueen et al., 1989). Disparate responses also occur upon application of the above neurotransmitters to carotid body preparations (*in vivo* and *in vitro*), including 1) biphasic responses that consisted of initial brief bursts in CSN activity followed by a more prolonged phase of depressed activity (Bisgard et al., 1979), 2) a biphasic pattern of responses consisting of initial brief decrease in CSN activity followed by long-lasting excitation (Matsumoto et al., 1981), 3) direct activation of primary glomus cells and/or chemoafferents (Lahiri et al., 1981; Matsumoto et al., 1981; Milsom and Sadig, 1983; Heinert et al., 1995; Pang et al., 1999), 4) direct inhibition of primary glomus cells and/or chemoafferents (Zapata et al., 1969; Zapata, 1975; Llados and Zapata, 1978; Mills, et al., 1978; Folgering et al., 1982; Kou et al., 1991; Pizarro et al., 1992; Bisgard et al., 1993; Prabhakar et al., 1993; Ryan et al., 1995; Almaraz, et al., 1997; Overholt and Prabhakar, 1999), and 5) constriction of arteriolar blood flow in carotid bodies leading to indirect excitation of carotid body glomus cells (Potter et al., 1987; Yokoyama et al., 2015).

As mentioned above, our studies in mice with bilateral CSCX or SCGX could not discriminate between the roles of SCG projections to the brain from those to the carotid bodies with respect to the ventilatory responses to HXC (Getsy et al., 2021a; Getsy et al., 2021b). We have not been able to confidently transect the GGN (GGNX) bilaterally in mice to date, but have successfully performed this surgery in juvenile P21 (21 days post-natal age) male Sprague Dawley rats to allow for direct comparison of potential findings to those we obtained following bilateral CSN transection (CSNX) in such rats (Getsy et al., 2020). We described in detail why we use juvenile rats and the reasons included that this is a pivotal age in their development and that P25 (day of actual testing) is the optimal day that we take the rats for electrophysiological experiments (Getsy et al., 2020). To directly address how the absence of GGN input to the carotid bodies affects the ventilatory responses that occur during and after episodes of HXC, the present study determined the responses that occurred during five successive episodes of HXC (10% O_2_, 90% N_2_) each separated by 15 min of room-air in 25 day-old (P25) male Sprague Dawley rats that had undergone sham-operation (SHAM rats) or bilateral GGNX (GGNX rats) 4 days earlier (P21).

## Materials and methods

### Animals and surgeries

**FIGURE 1:**
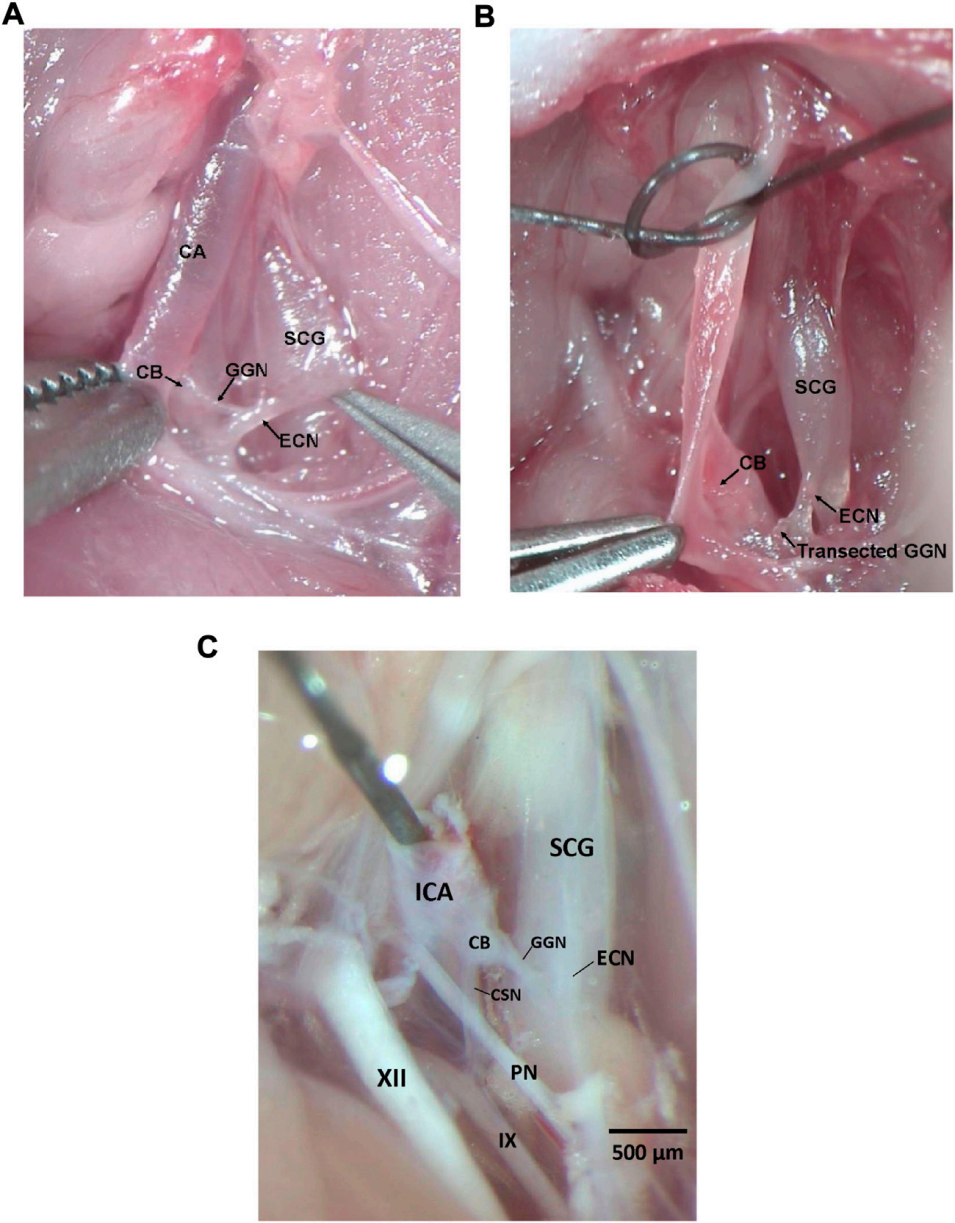
**(A)** Photograph in a male P25 juvenile Sprague Dawley rat under anesthesia showing the ganglioglomerular nerve (GGN), a branch off the external carotid nerve (ECN), entering the carotid body (CB). The ECN branches from the superior cervical ganglion (SCG) as shown. The carotid artery (CA) is also depicted. **(B)** Photograph in a male P25 juvenile Sprague Dawley rat under anesthesia showing the ganglioglomerular nerve (GGN), a branch off the external carotid nerve (ECN), transected. The ECN branches from the superior cervical ganglion (SCG) as shown. The carotid body (CB) is also depicted. **(C)** Photograph in a male P25 juvenile Sprague Dawley rat perfused with 4% paraformaldehyde showing the ganglioglomerular nerve (GGN), a branch off the external carotid nerve (ECN), entering the carotid body (CB), and the carotid sinus nerve (CSN) branching off the glossopharyngeal nerve (IX) and entering the CB. The ECN branches from the superior cervical ganglion (SCG) as shown. The pharyngeal nerve (PN), hypoglossal nerve (XII), and internal carotid artery (ICA) are also depicted. Dissections for panels **(A–C)** were done on the left side of the rat. Scale bar for all photos is 500 μm.

All experiments described here were carried out in strict accordance with the National Institute of Health Guide for the Care and Use of Laboratory Animals (NIH Publication No. 80–23) revised in 1996 (https://www.nap.edu/catalog/5140/guide-for-the-care-and-use-of-laboratory-animals). The protocols were approved by the Institutional Animal Care and Use Committee of Case Western Reserve University (Cleveland, OH). Ninety male Sprague Dawley (SD) rats (postnatal age 21, P21) from ENVIGO (Indianapolis, IN) were used in these studies. All of the rats were anesthetized with an intraperitoneal injection of ketamine (80 mg/kg, Ketaset, Zoetis, Parsippany, NJ) and xylazine (10 mg/kg, Akorn Animal Health, Lake Forest, IL), and placed on a surgical station to maintain body temperature at 37°C *via* a heating pad (SurgiSuite, Kent Scientific Corporation, Torrington, CT). The surgical plane of anesthesia was checked every 15 min by a toe pinch. Bilateral GGNX and sham-operations (SHAM) were performed. For bilateral (i.e., left and right side) GGNX, a midline incision of approximately 2 cm was made in the neck. Dual forceps were used to tease away connective tissue and expose the SCG behind the carotid artery bifurcation. Figure 1A shows the dissection to expose the GGN immediately before being transected and Figure 1B shows the GGN immediately after being transected. Briefly, in the anesthetized rat, the ECN was exposed as it exited the SCG and was followed until the GGN was located branching off the ECN. The GGN was then transected using micro-scissors at the point where it branched from the ipsilateral ECN. This procedure was performed on both the left and ride side. For SHAM procedures, the left and right GGN was identified but not transected. Figure 1C shows the anatomy in a paraformaldehyde P25 male rat to show the proximity of the CSN and other nerve structures.

The rats were allowed 4 days to recover from surgery and were P25 on the day of the study. All rats were monitored for pain and distress every day following surgery. Rats were given an injection of the non-steroidal anti-inflammatory drug, carprofen (2 mg/kg, Rimadyl, Zoetis, Parsippany, NJ), 24 and 48 h post-surgery to reduce any pain or inflammation at the incision site. None of the rats showed any signs of pain or inflammation from the surgeries and began moving about the cages and eating and drinking approximately 1 h after surgery. Rats were weighed daily to ensure proper weight gain. We have determined that these injections of carprofen do not affect resting ventilation or the responses to HXC on day 4 post-surgery (data not shown).

### Recording of ventilatory parameters

#### Protocols for whole body plethysmography measurement of ventilatory parameters

Ventilatory parameters were continuously recorded in the unanesthetized unrestrained SHAM or GGNX rats *via* whole body plethysmography (Buxco Small Animal Whole Body Plethysmography, DSI a division of Harvard Biosciences, Inc., St. Paul, MN, USA) as detailed previously (May et al., 2013a; May et al., 2013b; Young et al., 2013; Getsy et al., 2014; Henderson et al., 2014; Baby et al., 2018; Gaston et al., 2020; Getsy et al., 2020; Baby S. et al., 2021; Getsy et al., 2021a; Baby S. M. et al., 2021; Getsy et al., 2021b; Gaston et al., 2021; Seckler et al., 2022). The directly recorded and calculated (derived) parameters are defined in Supplementary Table S1 (Hamelmann et al., 1997; Lomask, 2006; Tsumuro et al., 2006; Quindry et al., 2016; Gaston et al., 2021). Directly recorded and derived ventilatory parameters and the abbreviations used in this manuscript are: frequency of breathing (Freq), tidal volume (TV), minute ventilation (MV), inspiratory time (Ti), expiratory time (Te), Ti/Te, end inspiratory pause (EIP), end expiratory pause (EEP), peak inspiratory flow (PIF), peak expiratory flow (PEF), PIF/PEF, expiratory flow at 50% expired TV (EF_50_), relaxation time (RT), inspiratory drive (TV/Ti), expiratory drive (TV/Te), apneic pause [(Te/RT)-1], non-eupneic breathing index (NEBI) and NEBI corrected for Freq (NEBI/Freq). A diagram of relationships between some directly recorded parameters and apneic pause (adapted from Lomask, 2006) are shown in Supplementary Figure S1. All studies were done in a quiet laboratory with atmospheric pressure of 760 mmHg (sea-level). The chamber volumes were 1.5 L and the room-air or gas flowing through each of the chambers was set at 1.5 L/min. The chamber temperatures during the acclimatization period were: 26.6 ± 0.1°C for the SHAM rats and 26.6 ± 0.1°C for GGNX rats (*p* > 0.05, GGNX *versus* SHAM). The chamber humidities during acclimatization were 52.1% ± 2.4% for SHAM rats and 50.8% ± 2.0% for GGNX rats (*p* > 0.05, GGNX *versus* SHAM). The FinePointe (DSI) software constantly corrected digitized ventilatory values originating from the actual waveforms for alterations in chamber humidity and chamber temperature. Pressure changes associated with the respiratory waveforms were converted to volumes (e.g., TV, PIF, PEF, EF_50_) employing the algorithms of Epstein and colleagues (Epstein and Epstein, 1978; Epstein et al., 1980). Specifically, factoring in chamber humidity and temperature, cycle analyzers filtered the acquired signals, and FinePointe algorithms generated an array of box flow data that identified a waveform segment as an acceptable breath. From that data vector, minimum and maximum box flow values were determined and multiplied by a compensation factor provided by selected algorithms (Epstein and Epstein, 1978; Epstein et al., 1980) thus producing TV, PIF and PEF values that were used to determine non-eupneic breathing events expressed as the non-eupneic breathing index (NEBI), reported as the percentage of non-eupneic breathing events per epoch (Getsy et al., 2014).

#### Protocols and data recording including maximal attainable responses

The rats were placed in plethysmography chambers to continuously record breath by breath ventilatory parameters. The rats were allowed to acclimatize for at least 60 min to allow stable baseline values to be recorded over a 15 min period prior to exposing the rats to HXC. After the rat was placed in the chamber, it usually explores the new environment for about 15–20 min and then would usually lay still, periodically grooming and occasionally sniffing the air. As such, at the time the hypoxic gas was delivered to the chambers, the rats were awake and resting quietly. The behavior of the rats did not change appreciably upon delivery of the HXC. The occasional rat explored the chamber for 5–10 s or groomed for 2–5 s. The rats were exposed to five 5-min episodes of a poikilocapnic hypoxic (10% O_2_, 90% N_2_) gas challenge each separated by 15 min of room-air. For each ventilatory parameter, data points collected during every 15 s epoch were averaged for each rat for graphing and analyses. The maximal values during each HXC did not necessarily occur during the same 15 s epoch in each rat, and so the maximal values obtained by each rat were also collected.

#### Data analysis

The directly recorded and arithmetically-derived parameters (1 min bins) were taken for statistical analyses. The Pre hypoxic gas challenge 1 min bins excluded occasional marked deviations from resting values due to abrupt movements by the rats, such as grooming or sniffing. The exclusions ensured accurate determination of baseline parameters. All data are presented as mean ± SEM and were evaluated using one-way and two-way ANOVA followed by Bonferroni corrections for multiple comparisons between means using the error mean square terms from each ANOVA analysis (Wallenstein et al., 1980; Ludbrook, 1998; McHugh, 2011) as detailed previously (Getsy et al., 2021a; Getsy et al., 2021b). A *p* < 0.05 value denoted the initial level of statistical significance that was modified according to the number of comparisons between means as described by Wallenstein et al. (1980). The modified *t-*statistic is t = (mean group 1—mean group 2)/[s x (1/n_1_ + 1/n_2_)^1/2^] where s^2^ = the mean square within groups term from the ANOVA (the square root of this value is used in the modified *t*-statistic formula) and n_1_ and n_2_ are the number of rats in each group under comparison. Based on an elementary inequality called Bonferroni’s inequality, a conservative critical value for modified *t*-statistics obtained from tables of *t*-distribution, using a significance level of P/m, where m is the number of comparisons between groups to be performed (Winer, 1971). The degrees of freedom are those for the mean square for within group variation from the ANOVA table. In the majority of situations, the critical Bonferroni value cannot be found in conventional tables of the t-distribution, but can be approximated from tables of the normal curve by t* = z + (z + z^3^)/4n, with n being the degrees of freedom and z being the critical normal curve value for P/m (Wallenstein et al., 1980; Ludbrook, 1998; McHugh, 2011). Wallenstein et al. (1980) first demonstrated that the Bonferroni procedure is preferable for general use since it is easy to apply, has the widest range of applications, and provides critical values that are lower than those of other procedures when the investigator can limit the number of comparisons and will be slightly larger than those of other procedures if many comparisons are made. As mentioned, a value of *p* < 0.05 was taken as the initial level of statistical significance (Wallenstein et al., 1980; Ludbrook, 1998; McHugh, 2011 and statistical analyses were performed with the aid of GraphPad Prism software (GraphPad Software, Inc., La Jolla, CA).

## Results

### Resting ventilatory values

A summary of the numbers of rats in the SHAM and GGNX groups, and their ages and body weights are provided in Supplementary Table S2. There were no between-group differences in the ages or body weights (*p* > 0.05 for both comparisons). The baseline (Pre-HX challenge) values recorded in the SHAM and GGNX rats are also summarized in Supplementary Table S2. There were no between-group differences for most of the parameters (*p* > 0.05, for all comparisons). However, resting Freq was lower in GGNX rats than in SHAM rats and this was associated with higher Te and EEP values in GGNX rats than in SHAM rats. In addition, the PEF/PIF quotient was higher in the GGNX rats than in SHAM rats. Finally, although NEBI was lower in the GGNX rats due to their lower Freq values, the NEBI/Freq quotient was similar in GGNX and SHAM rats.

### Hypoxic gas challenges

#### Freq, TV and MV responses

**FIGURE 2:**
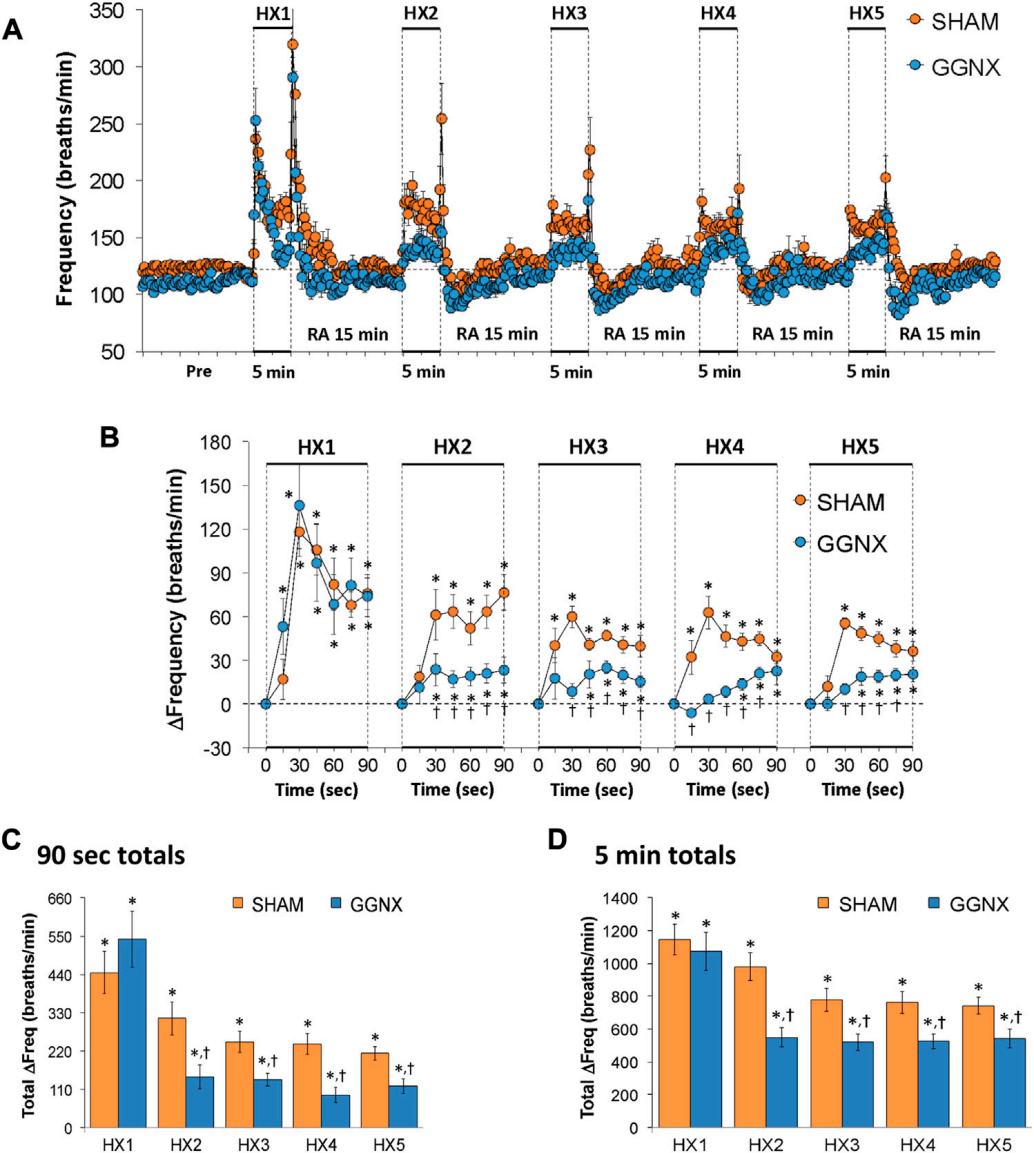
**(A)** Frequency of breathing in sham-operated (SHAM) rats and in rats with bilateral ganglioglomerular nerve transection (GGNX) before (Pre) and during five hypoxic (HX, 10% O_2_, 90% N_2_) gas challenges, each separated by 15 min of room-air (RA). **(B)** Arithmetic changes in frequency during the first 90 s of HX gas challenge. **(C)** Total changes in frequency during the first 90 s of HX gas challenge. **(D)** Total changes in frequency during the entire 5 min of HX gas challenge. The SHAM group had 10 rats. The GGNX group had 12 rats. The data are presented as mean ± SEM. *p < 0.05, significant response. ^†^ *p* < 0.05, GGNX rats *versus* SHAM rats.

**FIGURE 3:**
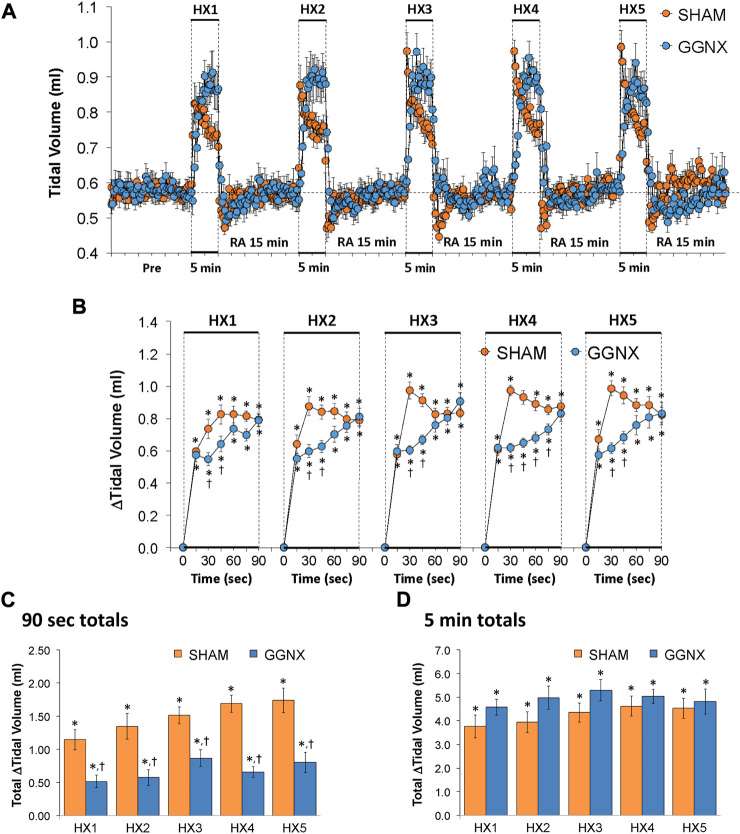
**(A)** Tidal volume in sham-operated (SHAM) rats and in rats with bilateral ganglioglomerular nerve transection (GGNX) before (Pre) and during five hypoxic (HX, 10% O_2_, 90% N_2_) gas challenges, each separated by 15 min of room-air (RA). **(B)** Arithmetic changes in tidal volume during the first 90 s of HX gas challenge. **(C)** Total changes in tidal volume during the first 90 s of HX gas challenge. **(D)** Total changes in tidal volume during the entire 5 min of HX gas challenge. The SHAM group had 10 rats. The GGNX group had 12 rats. The data are presented as mean ± SEM. *p < 0.05, significant response. ^†^ *p* < 0.05, GGNX rats *versus* SHAM rats.

**FIGURE 4:**
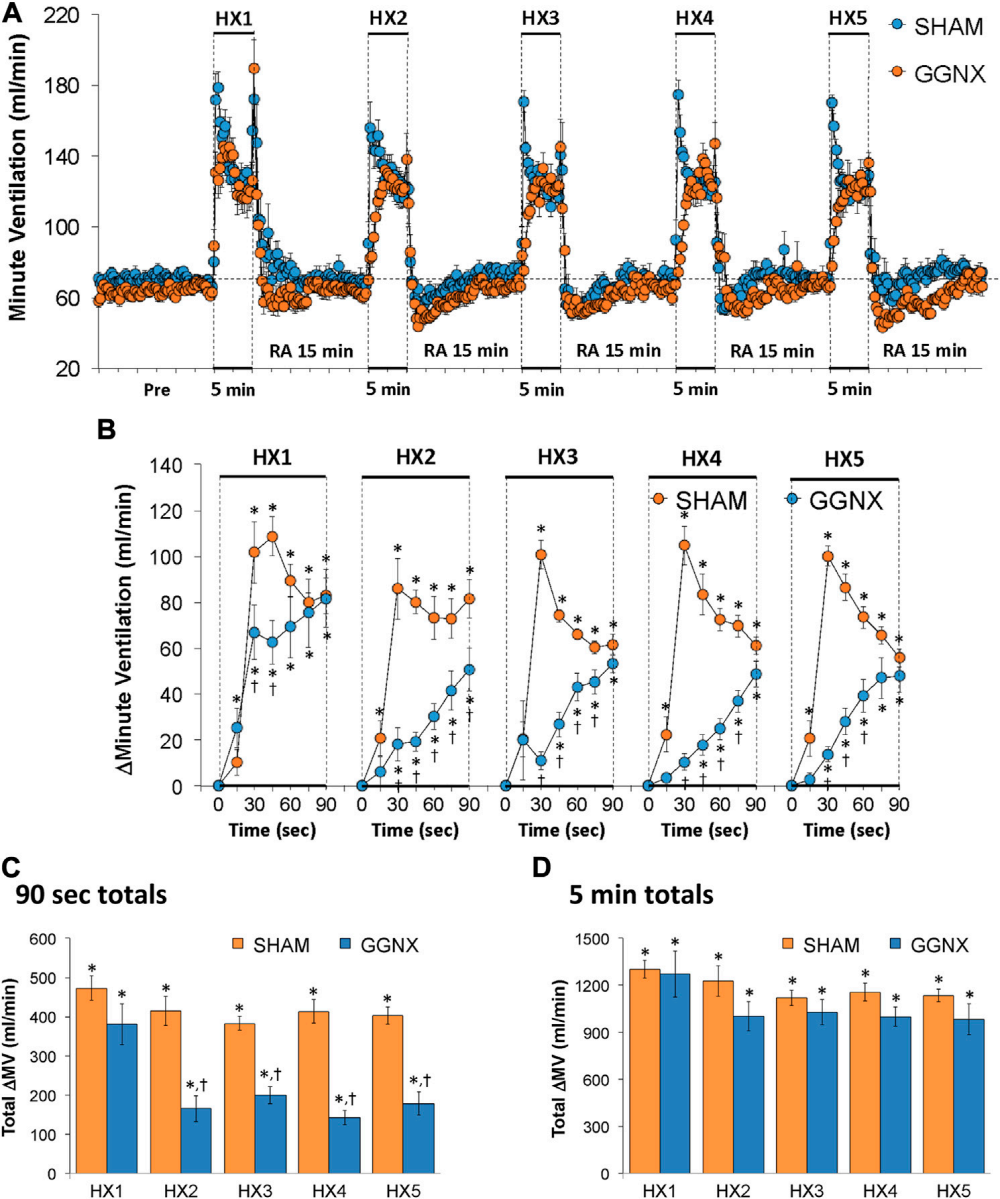
**(A)** Minute ventilation in sham-operated (SHAM) rats and in rats with bilateral ganglioglomerular nerve transection (GGNX) before (Pre) and during five hypoxic (HX, 10% O_2_, 90% N_2_) gas challenges, each separated by 15 min of room-air (RA). **(B)** Arithmetic changes in minute ventilation during the first 90 s of HX gas challenge. **(C)** Total changes in minute ventilation during the first 90 s of HX gas challenge. **(D)** Total changes in minute ventilation during the entire 5 min of HX gas challenge. The SHAM group had 10 rats. The GGNX group had 12 rats. The data are presented as mean ± SEM. *p < 0.05, significant response. ^†^ *p* < 0.05, GGNX rats *versus* SHAM rats.

Freq values in SHAM and GGNX rats before and during five HXC (10% O_2_, 90% N_2_) each separated by 15 min of room-air are shown in Figure 2A. Each HXC elicited robust increases in Freq that rapidly declined upon return to room-air in the SHAM and GGNX rats. The initial (1–90 s) responses to the first HXC (HXC 1) were similar in both groups Figure 2B whereas the initial responses to HXC 2-5 were markedly smaller in GGNX rats. The total responses recorded over the first 90 s Figure 2C and the entire 5 min Figure 2D of HXC 2-5 were smaller in GGNX rats than SHAM rats. TV responses: TV values in SHAM and GGNX rats before and during five HXC (10% O_2_, 90% N_2_) each separated by 15 min of room-air are shown in of Figure 3A. Each HXC elicited robust increases in TV that rapidly declined to below baseline levels upon return to room-air in SHAM and GGNX rats. It was evident that the increases in TV in GGNX rats were higher than SHAM rats at the later phases of HXC 1-5. The initial responses (1–90 s) during HXC 1-5 were smaller in GGNX rats than SHAM rats Figure 3B. The total responses recorded over the first 90 s of HXC 1-5 were smaller in GGNX rats Figure 3C whereas the responses recorded over the entire 5 min of HXC 2-5 were similar in the GGNX and SHAM rats Figure 3D. MV responses: MV values in SHAM and GGNX rats before and during five HXC (10% O_2_, 90% N_2_) each separated by 15 min of room-air are shown in of Figure 4A. Each HXC elicited robust increases in MV that rapidly declined to and often below baseline levels upon return to room-air in the SHAM and GGNX rats. The initial (1–90 s) responses during HXC 1-5 were smaller in GGNX rats than in SHAM rats Figure 4B. The total responses recorded over the first 90 s of HXC 2-5 were markedly smaller in GGNX rats than in SHAM rats Figure 4C. The total responses recorded over the entire 5 min of HXC 1-5 were similar in the GGNX and SHAM rats Figure 4D.

#### Freq, TV and MV responses in the later period of HXC 1-5

**FIGURE 5:**
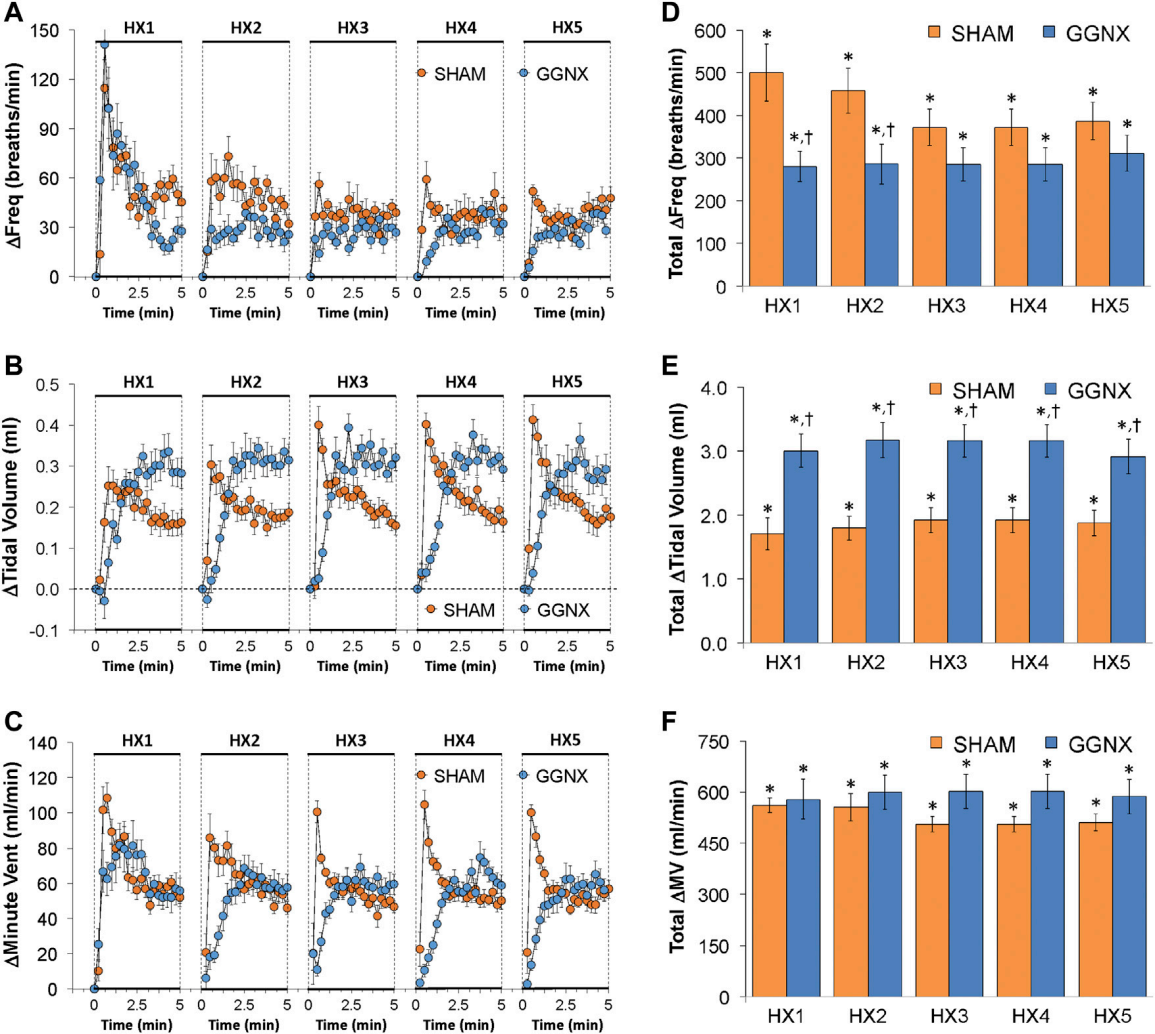
Arithmetic changes in the frequency of breathing **(A)**, tidal volume **(B)** and minute ventilation **(C)** in sham-operated (SHAM) rats and in rats with bilateral ganglioglomerular nerve transection (GGNX) during the five hypoxic (HX, 10% O_2_, 90% N_2_) gas challenges. Total changes in frequency of breathing **(D)**, tidal volume **(E)** and minute ventilation **(F)** during the final 2.5 min of HX gas challenge. The SHAM group had 10 rats. The GGNX group had 12 rats. The data are presented as mean ± SEM. *p < 0.05, significant response. ^†^ *p* < 0.05, GGNX rats *versus* SHAM rats.

The arithmetic changes in Freq, TV and MV during the later period of HXC 1-5 are presented in Figures 5A–C, respectively). The data confirm that the initial increases in Freq, TV and MV were smaller in GGNX rats than in SHAM rats and that the increases in TV (but not Freq or MV) were greater at the later stages of HXC 1-5 in GGNX rats than in SHAM rats. As such, the total increases in Freq between 2.5 and 5 min of HXC 1-2 were smaller in GGNX rats than in SHAM rats Figure 5D, whereas the increases in TV recorded between 2.5 and 5 min of HXC 1-5 were greater in GGNX rats than in SHAM rats Figure 5E. As a result, the total increases in MV recorded between 2.5 and 5 min of HXC 1-5 were similar in GGNX and SHAM rats Figure 5F.

#### Ti responses

**FIGURE 6:**
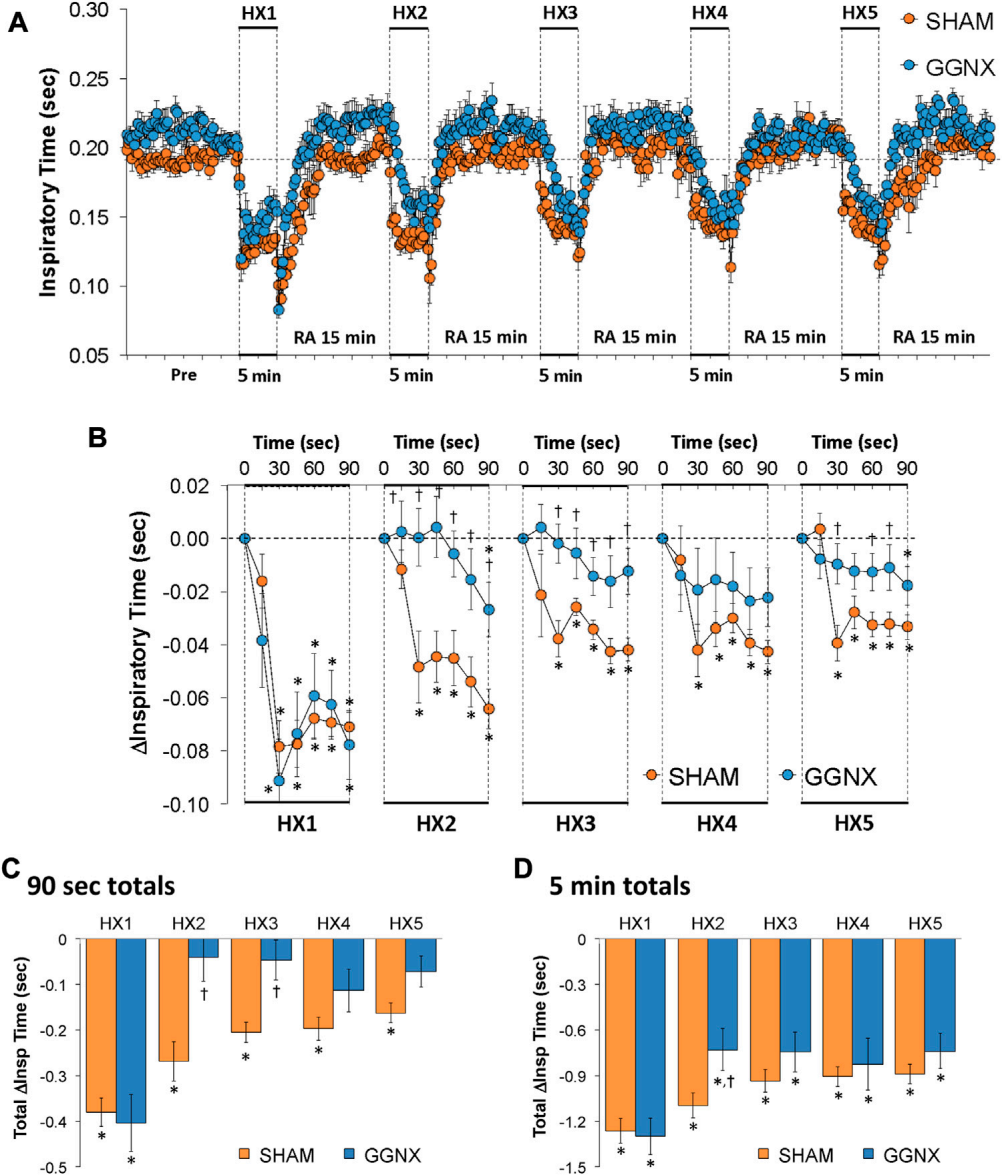
**(A)** Inspiratory time in sham-operated (SHAM) rats and in rats with bilateral ganglioglomerular nerve transection (GGNX) before (Pre) and during five hypoxic (HX, 10% O_2_, 90% N_2_) gas challenges, each separated by 15 min of room-air (RA). **(B)** Arithmetic changes in inspiratory time during the first 90 s of HX gas challenge. **(C)** Total changes in inspiratory time during the first 90 s of HX gas challenge. **(D)** Total changes in inspiratory time during the entire 5 min of HX gas challenge. The SHAM group had 10 rats. The GGNX group had 12 rats. The data are presented as mean ± SEM. *p < 0.05, significant response. ^†^ *p* < 0.05, GGNX rats *versus* SHAM rats.

Ti values in SHAM and GGNX rats before and during five HXC (10% O_2_, 90% N_2_) each separated by 15 min of room-air are shown in Figure 6A. Each HXC elicited robust decreases in Ti that rapidly returned to baseline levels upon return to room-air in SHAM and GGNX rats. The initial (1–90 s) responses during HXC 2-5 were smaller in GGNX rats than in SHAM rats Figure 6B. The total responses recorded over the first 90 s of HXC 2-3 were markedly smaller in the GGNX rats than SHAM rats Figure 6C, whereas the total responses recorded over the entire 5 min of HXC 1-5 were smaller in GGNX rats than SHAM rats for HXC 2 only Figure 6D.

#### Te responses

**FIGURE 7:**
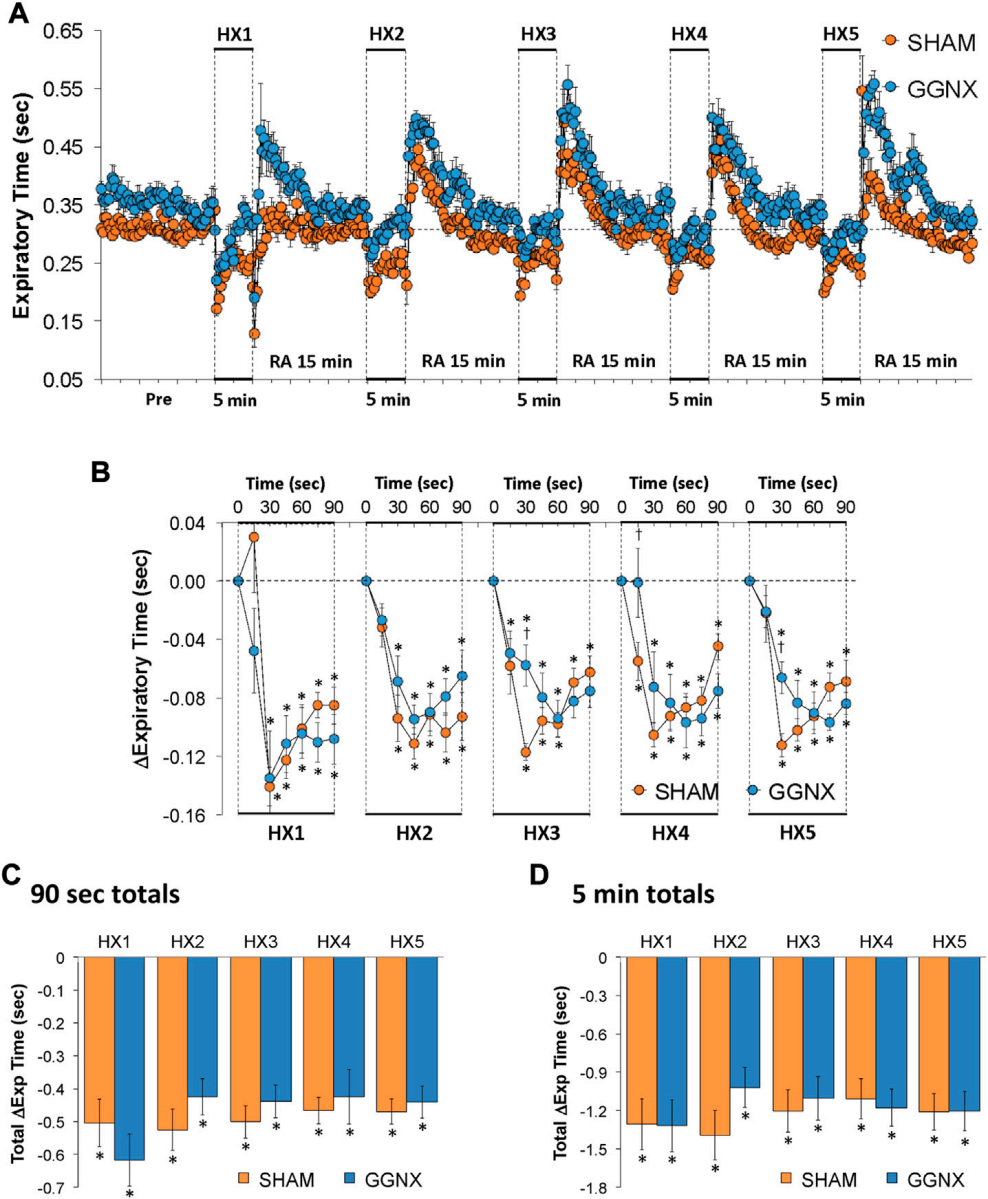
**(A)** Expiratory time in sham-operated (SHAM) rats and in rats with bilateral ganglioglomerular nerve transection (GGNX) before (Pre) and during five hypoxic (HX, 10% O_2_, 90% N_2_) gas challenges, each separated by 15 min of room-air (RA). **(B)** Arithmetic changes in expiratory time during the first 90 s of HX gas challenge. **(C)** Total changes in expiratory time during the first 90 s of HX gas challenge. **(D)** Total changes in expiratory time during the entire 5 min of HX gas challenge. The SHAM group had 10 rats. The GGNX group had 12 rats. The data are presented as mean ± SEM. *p < 0.05, significant response. ^†^ *p* < 0.05, GGNX rats *versus* SHAM rats.

Te values in SHAM and GGNX rats before and during five HXC (10% O_2_, 90% N_2_) each separated by 15 min of room-air are shown in of Figure 7A. Each HXC elicited robust decreases in Te Figure 7A that rapidly returned to and greatly exceeded baseline levels upon return to room-air in SHAM and GGNX rats. The initial (1–90 s) responses during HXC 1-5 were similar in SHAM and GGNX rats. The total responses recorded over the first 90 s Figure 7C or the entire 5 min Figure 7D of HXC 1-5 were similar in SHAM and GGNX rats.

#### Te/Ti responses

Te/Ti values in SHAM and GGNX rats before and during five HXC (10% O_2_, 90% N_2_) each separated by 15 min of room-air are shown in of Supplementary Figure S2A. Each HXC elicited robust decreases in Te/Ti that rapidly returned to and greatly exceeded baseline upon return to room-air in SHAM and in GGNX rats. The initial (1–90 s) responses during HXC 1-5 were similar in SHAM and GGNX rats Supplementary Figure S2B. The total decrease in Te/Ti over the first 90 s Supplementary Figure S2C was markedly greater in GGNX rats compared to SHAM rats in HXC 2 and similar in HXC 1, 3, 4 and 5. The total responses over the entire 5 min Supplementary Figure S2D of HXC 1-5 were similar in SHAM and GGNX rats.

#### EIP responses

EIP values in SHAM and GGNX rats before and during five HXC (10% O_2_, 90% N_2_) each separated by 15 min of room-air are shown in Supplementary Figure S3A. Each HXC elicited robust decreases in EIP in the SHAM and GGNX rats that rapidly returned to baseline levels upon return to room-air. The initial (1–90 s) responses during HXC 1-5 were similar in SHAM and GGNX rats (Supplementary Figure S3B). The total responses recorded over the first 90 s of HXC 1-5 were similar in SHAM and GGNX rats Supplementary Figure S3C. The total decreases in EIP recorded over the entire 5 min of HXC 1, 3 and 4 were greater in GGNX than SHAM rats Supplementary Figure S3D.

#### EEP responses

**FIGURE 8:**
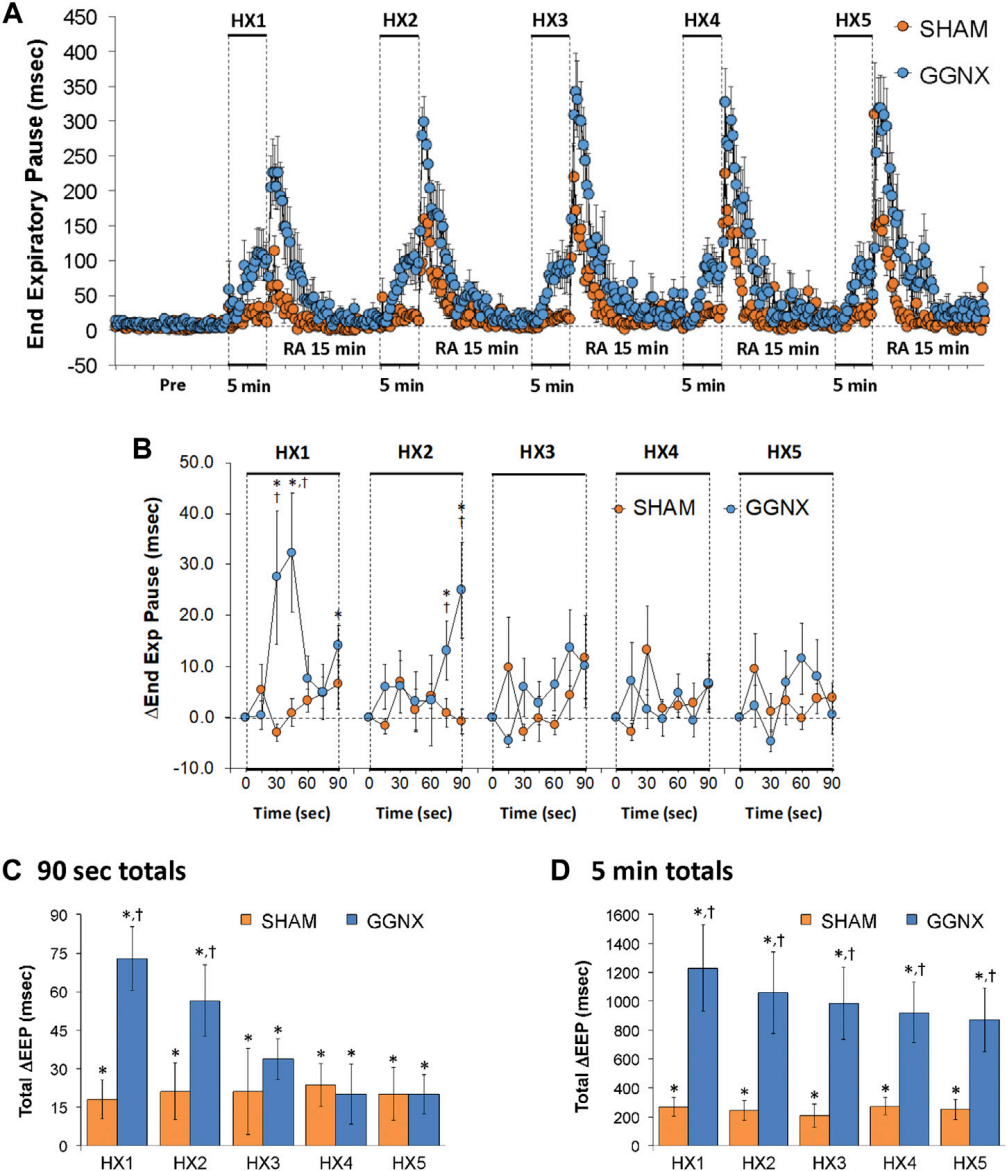
**(A)** End Expiratory Pause (EEP) values in sham-operated (SHAM) rats and in rats with bilateral ganglioglomerular nerve transection (GGNX) before (Pre) and during five hypoxic (HX, 10% O_2_, 90% N_2_) gas challenges, each separated by 15 min of room-air (RA). **(B)** Arithmetic changes in EEP during the first 90 s of HX gas challenge. **(C)** Total changes in EEP during the first 90 s of HX gas challenge. **(D)** Total changes in EEP during the entire 5 min of HX gas challenge. The SHAM group had 10 rats. The GGNX group had 12 rats. The data are presented as mean ± SEM. *p < 0.05, significant response. ^†^ *p* < 0.05, GGNX rats *versus* SHAM rats.

EEP values in SHAM and GGNX rats before and during five HXC (10% O_2_, 90% N_2_) each separated by 15 min of room-air are shown in of Figure 8A. Each HXC elicited minimal changes in EEP in SHAM rats, but sustained increases in GGNX rats. These responses were followed by rapid increases in EEP upon return to room-air in SHAM and GGNX rats. The initial (1–90 s) responses during HXC 1-5 were similar in SHAM and GGNX rats Figure 8B. The total increases recorded over the first 90 s of HXC 1-2 were greater in GGNX than SHAM rats Figure 8C. The total increases recorded over the entire 5 min of HXC 1-5 were greater in GGNX rats than SHAM rats Figure 8D.

#### PIF responses

**FIGURE 9:**
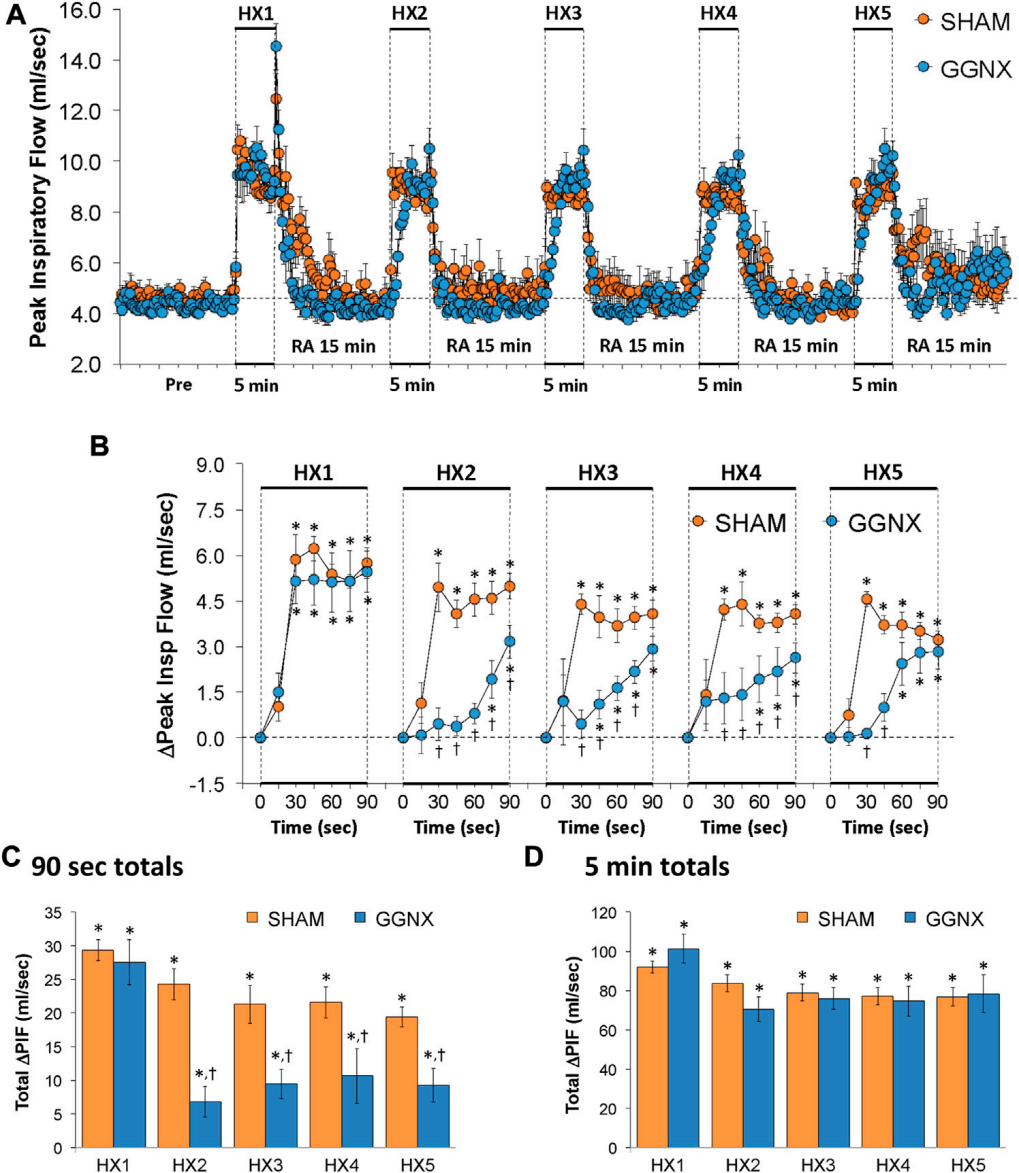
**(A)** Peak Inspiratory Flow (Peak Insp Flow; PIF) values in sham-operated (SHAM) rats and in rats with bilateral ganglioglomerular nerve transection (GGNX) before (Pre) and during five hypoxic (HX, 10% O_2_, 90% N_2_) gas challenges, each separated by 15 min of room-air (RA). **(B)** Arithmetic changes in PIF during the first 90 s of HX gas challenge. **(C)** Total changes in PIF during the first 90 s of HX gas challenge. **(D)** Total changes in PIF during the entire 5 min of HX gas challenge. The SHAM group had 10 rats. The GGNX group had 12 rats. The data are presented as mean ± SEM. *p < 0.05, significant response. ^†^ *p* < 0.05, GGNX rats *versus* SHAM rats.

PIF values in SHAM and GGNX rats before and during five HXC (10% O_2_, 90% N_2_) each separated by 15 min of room-air are shown in of Figure 9A. Each HXC elicited pronounced increases in PIF in the SHAM and GGNX rats that rapidly returned to baseline levels upon return to room-air. The initial (1–90 s) responses during HXC 2-5 were similar markedly smaller in GGNX rats as compared to SHAM rats Figure 9B. The total responses recorded over the first 90 s of HXC 2-5 were smaller in SHAM rats compared to GGNX rats Figure 9C, whereas the total increases recorded over the entire 5 min of HXC 1-5 were similar in SHAM and GGNX Figure 9D.

#### PEF responses

**FIGURE 10:**
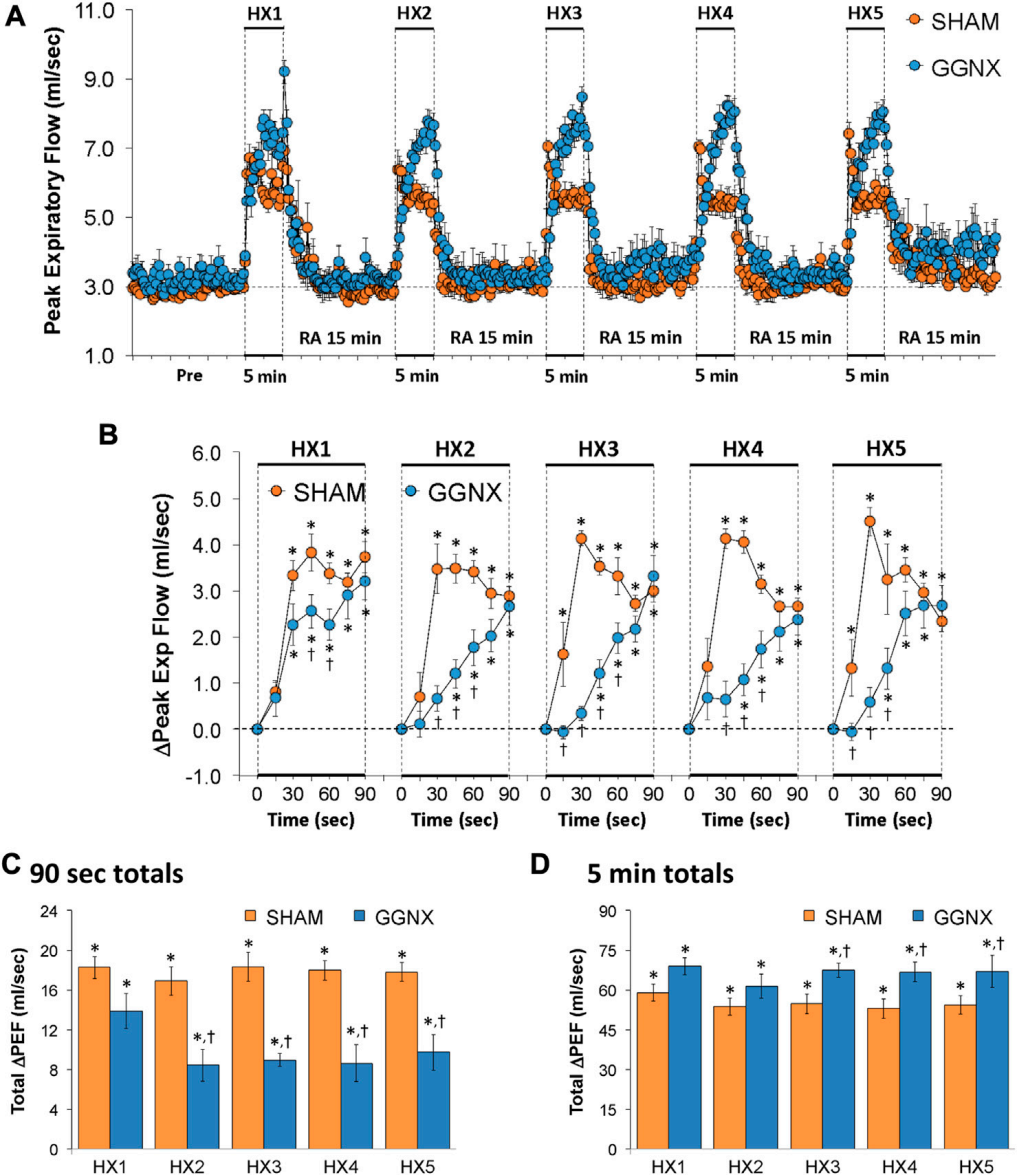
**(A)** Peak Expiratory Flow (Peak Exp Flow, PEF) values in sham-operated (SHAM) rats and in rats with bilateral ganglioglomerular nerve transection (GGNX) before (Pre) and during five hypoxic (HX, 10% O_2_, 90% N_2_) gas challenges, each separated by 15 min of room-air (RA). **(B)** Arithmetic changes in PEF during the first 90 s of HX gas challenge. **(C)** Total changes in PEF during the first 90 s of HX gas challenge. **(D)** Total changes in PEF during the entire 5 min of HX gas challenge. The SHAM group had 10 rats. The GGNX group had 12 rats. The data are presented as mean ± SEM. *p < 0.05, significant response. ^†^ *p* < 0.05, GGNX rats *versus* SHAM rats.

PEF values in SHAM and GGNX rats before and during five HXC (10% O_2_, 90% N_2_) each separated by 15 min of room-air are shown in of Figure 10A. Each HXC elicited pronounced increases in PEF in the SHAM and GGNX rats that rapidly returned to baseline levels upon return to room-air. The initial (1–90 s) responses during HXC 1-5 were smaller in the GGNX rats than in the SHAM rats Figure 10B. The total responses recorded over the first 90 s of HXC 2-5 were smaller in GGNX than SHAM rats Figure 10C. The total increases recorded over the entire 5 min of HXC 3-5 were larger in GGNX and SHAM rats Figure 10D, with the differences being particularly pronounced during the second half of each 5 min HXC as seen in Figure 10A.

#### PEF/PIF responses

PEF/PIF values in the SHAM and GGNX rats before and during five HXC (10% O_2_, 90% N_2_) each separated by 15 min of room-air are shown in Supplementary Figure S4A. Each HXC elicited pronounced increases in PEF/PIF in SHAM and GGNX rats that were far larger in GGNX rats from about 2 min onward, and which rapidly returned to baseline levels upon return to room-air. PEF/PIF changed minimally over the initial (1–90 s) phase of HXC 1 in the SHAM rats, whereas it trended below baseline in the GGNX rats Supplementary Figure S4B. The initial increases in PEF/PIF during HXC 2 tended to be greater in the GGNX rats, whereas the PEF/PIF ratio tended to be smaller in in the GGNX rats for HXC 3–5. The total responses recorded over the first 90 s of HXC 1 were smaller in the GGNX rats than the SHAM rats, whereas the responses were greater in the GGNX rats than in the SHAM rats for HXC 2 Supplementary Figure S4C. The total increases recorded over the entire 5 min of HXC 2-5 were substantially larger in the GGNX rats as compared to the SHAM rats Supplementary Figure S4D.

#### EF_50_ responses

**FIGURE 11:**
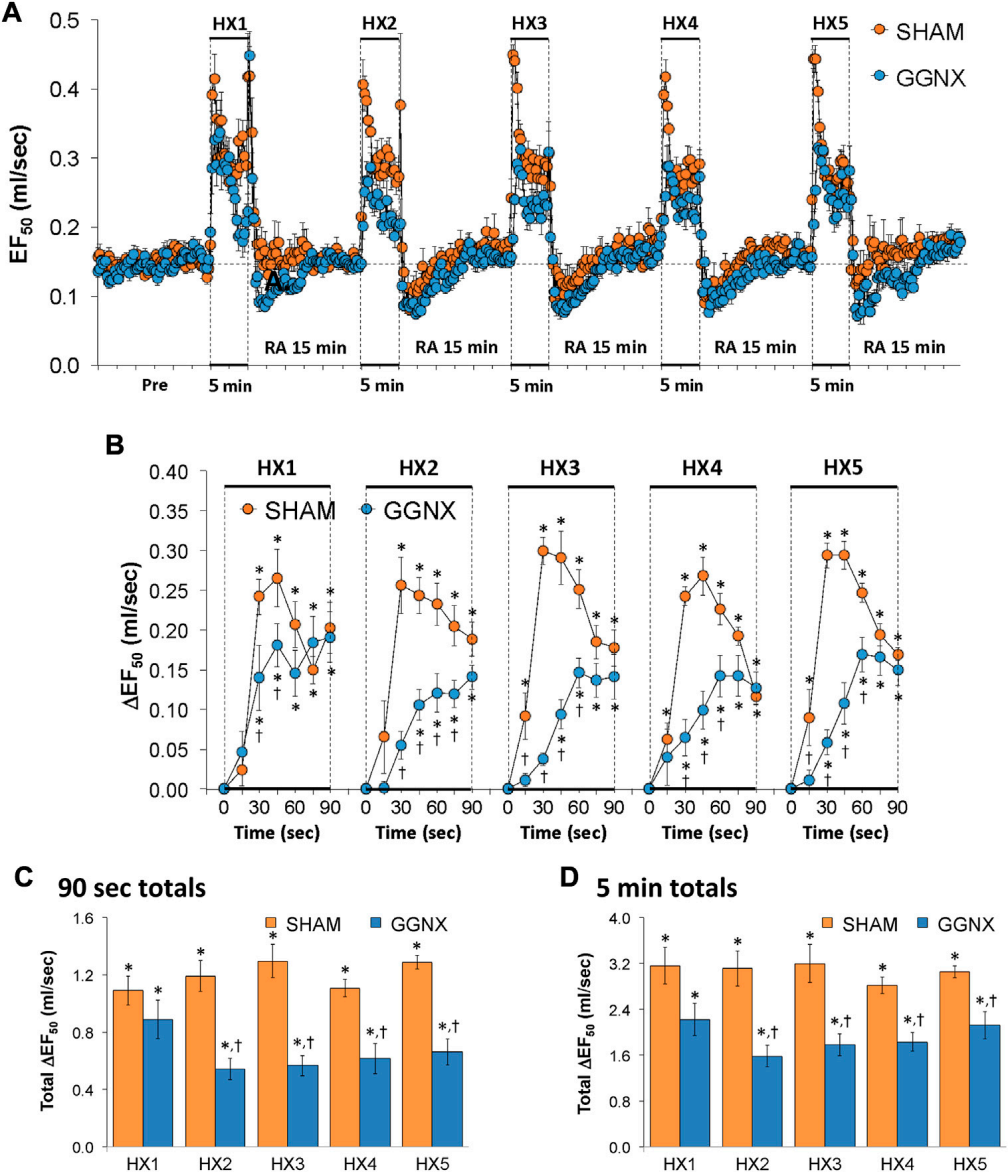
**(A)** EF_50_ values in sham-operated (SHAM) rats and in rats with bilateral ganglioglomerular nerve transection (GGNX) before (Pre) and during five hypoxic (HX, 10% O_2_, 90% N_2_) gas challenges, each separated by 15 min of room-air (RA). **(B)** Arithmetic changes in EF_50_ during the first 90 s of HX gas challenge. Total changes in EF_50_ during the first 90 s **(C)** and over the entire 5 min **(D)** of HX gas challenge. The SHAM group had 10 rats. The GGNX group had 12 rats. The data are presented as mean ± SEM. *p < 0.05, significant response. ^†^ *p* < 0.05, GGNX rats *versus* SHAM rats.

EF_50_ values in SHAM and GGNX rats before and during five HXC (10% O_2_, 90% N_2_) each separated by 15 min of room-air are shown in of Figure 11A. Each HXC elicited pronounced increases in EF_50_ in the SHAM and GGNX rats that rapidly declined toward and often below baseline levels upon return to room-air in the SHAM and GGNX rats. The initial (1–90 s) responses during HXC 1-5 were smaller in GGNX rats than in SHAM rats Figure 11B. The total responses recorded over the first 90 s of HXC 2-5 were smaller in GGNX than SHAM rats Figure 11C. The total increases recorded over the entire 5 min of HXC 2-5 were smaller in GGNX rats than in SHAM rats Figure 11D.

#### Relaxation Time responses

Relaxation time values in the SHAM and GGNX rats before and during five HXC (10% O_2_, 90% N_2_) each separated by 15 min of room-air are shown in of Supplementary Figure S5A. Each HXC elicited transient decreases in relaxation time in the SHAM and GGNX rats, whereas the return to room-air resulted in dramatic increases in relaxation time in SHAM and GGNX rats. The initial (1–90 s) decreases in relaxation times during HXC 1-5 were similar in the SHAM and GGNX rats with a few time-points at which the responses were greater in the GGNX rats Supplementary Figure S5B. The total responses recorded over the first 90 s Supplementary Figure S5C and during the entire 5 min of HXC 1-5 were similar in the SHAM and GGNX rats Supplementary Figure S5D.

#### Apneic pause responses

Apneic pause values in SHAM and GGNX rats before and during five HXC (10% O_2_, 90% N_2_) each separated by 15 min of room-air are shown in Supplementary Figure S6A. Each HXC elicited minimal changes in apneic pause in the SHAM and GGNX rats, whereas the return to room-air caused transient increases in apneic pause in SHAM and GGNX rats that were especially evident following HXC 4 and HXC 5. The initial (1–90 s) changes in apneic pause during HXC 1-5 were similar in the SHAM and GGNX rats with a few time-points at which the responses were smaller in the GGNX rats Supplementary Figure S6B. The total responses recorded over the first 90 s Supplementary Figure S6C and during the entire 5 min of HXC 1-5 were similar in the SHAM and GGNX rats Supplementary Figure S6D.

#### Inspiratory Drive responses

**FIGURE 12:**
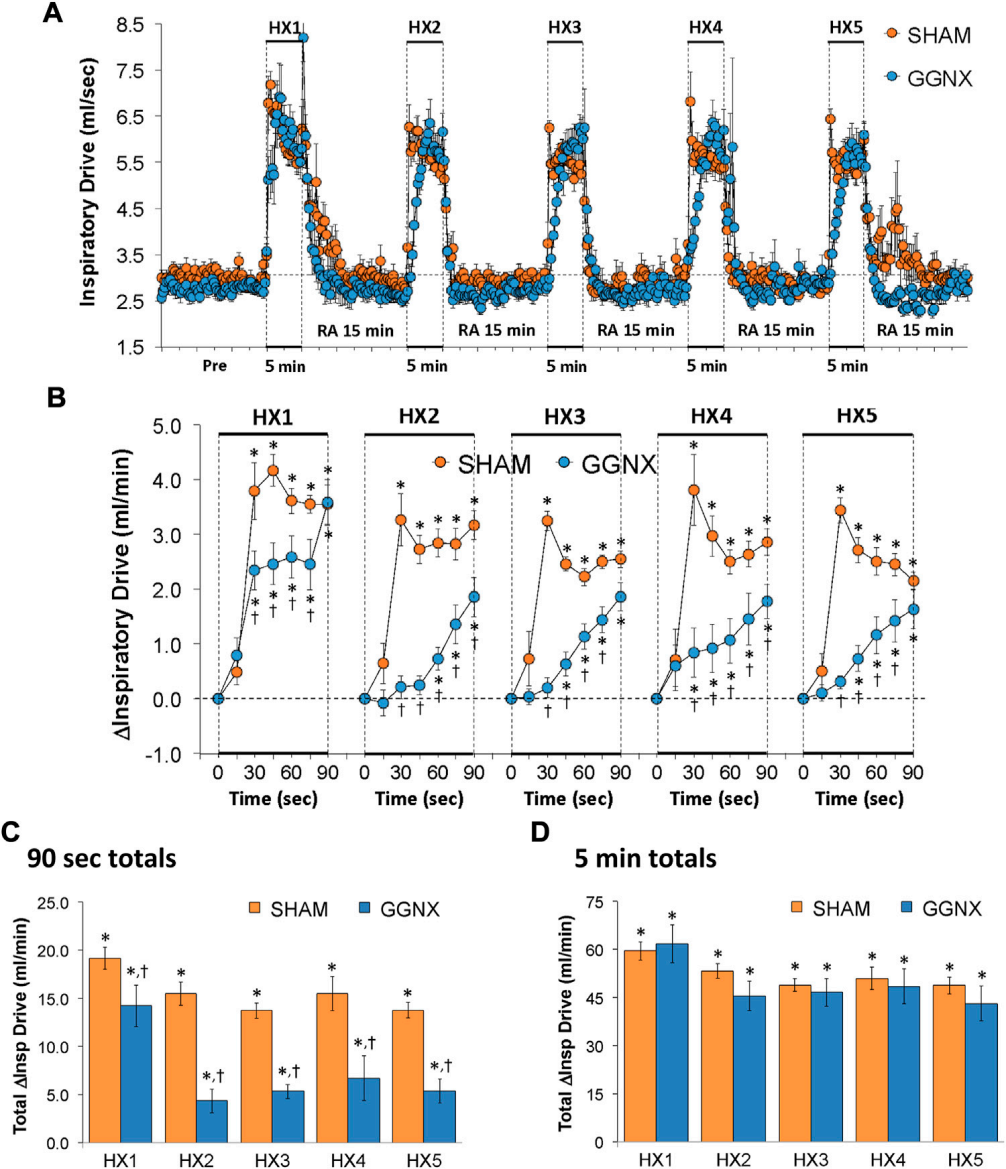
**(A)** Inspiratory Drive (Insp Drive) values in sham-operated (SHAM) rats and in rats with bilateral ganglioglomerular nerve transection (GGNX) before (Pre) and during five hypoxic (HX, 10% O_2_, 90% N_2_) gas challenges, each separated by 15 min of room-air (RA). **(B)** Arithmetic changes in Inspiratory Drive during the first 90 s of HX gas challenge. Total changes in Inspiratory Drive during the first 90 s **(C)** and over the entire 5 min **(D)** of HX gas challenge. The SHAM group had 10 rats. The GGNX group had 12 rats. The data are presented as mean ± SEM. *p < 0.05, significant response. ^†^ *p* < 0.05, GGNX rats *versus* SHAM rats.

Inspiratory Drive values in SHAM and GGNX rats before and during five HXC (10% O_2_, 90% N_2_) each separated by 15 min of room-air are shown in Figure 12A. Each HXC elicited pronounced increases in Inspiratory Drive in SHAM and GGNX rats that rapidly returned to baseline values upon return to room-air. The initial (1–90 s) increases in Inspiratory Drive during HXC 1-5 were markedly smaller in the GGNX rats than in the SHAM rats Figure 12B. The total responses recorded over the first 90 s of HXC 1-5 were smaller in the GGNX than SHAM rats Figure 12C. The total responses recorded during the entire 5 min of HXC 1-5 were similar in the SHAM and GGNX rats Figure 12D.

#### Expiratory drive responses

**FIGURE 13:**
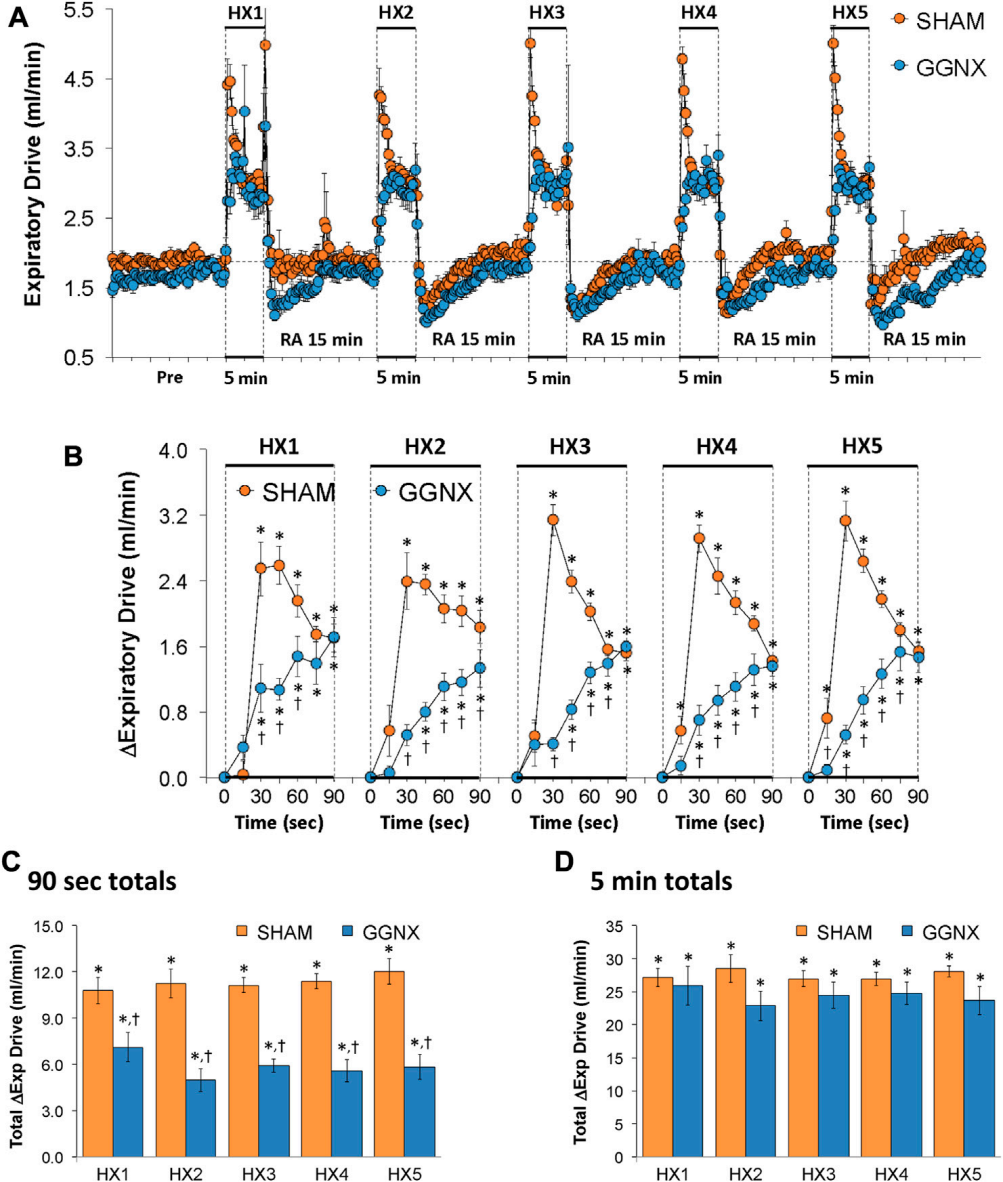
**(A)** Expiratory Drive (Exp Drive) values in sham-operated (SHAM) rats and in rats with bilateral ganglioglomerular nerve transection (GGNX) before (Pre) and during five hypoxic (HX, 10% O_2_, 90% N_2_) gas challenges, each separated by 15 min of room-air (RA). **(B)** Arithmetic changes in Expiratory Drive during the first 90 s of HX gas challenge. Total changes in Expiratory Drive during the first 90 s **(C)** and over the entire 5 min **(D)** of HX gas challenge. The SHAM group had 10 rats. The GGNX group had 12 rats. The data are presented as mean ± SEM. *p < 0.05, significant response. ^†^ *p* < 0.05, GGNX rats *versus* SHAM rats.

Expiratory Drive values in SHAM and GGNX rats before and during five HXC (10% O_2_, 90% N_2_) each separated by 15 min of room-air are shown in Figure 13A. Each HXC elicited pronounced increases in Expiratory Drive in SHAM and GGNX rats that rapidly fell at or below baseline values upon return to room-air. The initial (1–90 s) increases in Expiratory Drive during HXC 1-5 were markedly smaller in GGNX rats than in SHAM rats Figure 13B. The total responses recorded over the first 90 s of HXC 1-5 were smaller in GGNX than SHAM rats Figure 13C. The total responses recorded during the entire 5 min of HXC 1-5 were similar in the SHAM and GGNX rats Figure 13D.

#### NEBI responses

**FIGURE 14:**
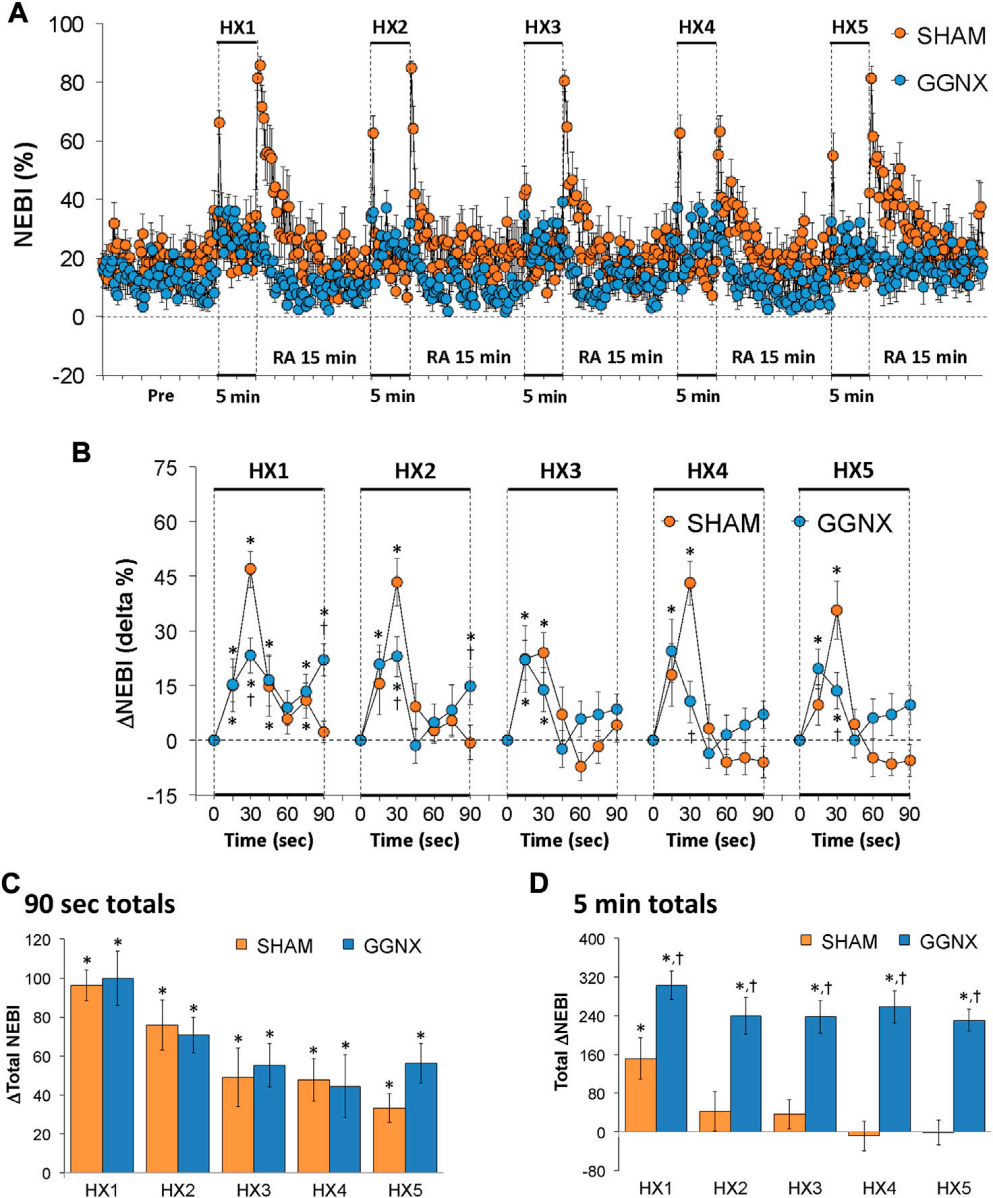
**(A)** Non-eupneic breathing index (NEBI) values in sham-operated (SHAM) rats and in rats with bilateral ganglioglomerular nerve transection (GGNX) before (Pre) and during five hypoxic (HX, 10% O_2_, 90% N_2_) gas challenges, each separated by 15 min of room-air (RA). **(B)** Arithmetic changes in NEBI during the first 90 s of HX gas challenge. Total changes in NEBI during the first 90 s **(C)** and over the entire 5 min **(D)** of HX gas challenge. The SHAM group had 10 rats. The GGNX group had 12 rats. Data are presented as mean ± SEM. *p < 0.05, significant response. ^†^ *p* < 0.05, GGNX rats *versus* SHAM rats.

NEBI values in SHAM and GGNX rats before and during five HXC (10% O_2_, 90% N_2_) each separated by 15 min of room-air are shown in Figure 14A. Each HXC elicited relatively minor increases in NEBI in SHAM and GGNX rats that transiently increased upon return to room-air in the SHAM rats, but not in the GGNX rats. The initial (1–90 s) increases in NEBI during HXC 1-5 were in general similar in SHAM and GGNX rats with a few time-points in which the responses were smaller in the GGNX rats Figure 14B. The total increases recorded over the first 90 s of HXC 1-5 were similar in the SHAM and GGNX rats Figure 14C. The total increases recorded during the entire 5 min of HXC 1-5 were greater in the GGNX rats than SHAM rats Figure 14D.

#### NEBI/Freq responses

**FIGURE 15:**
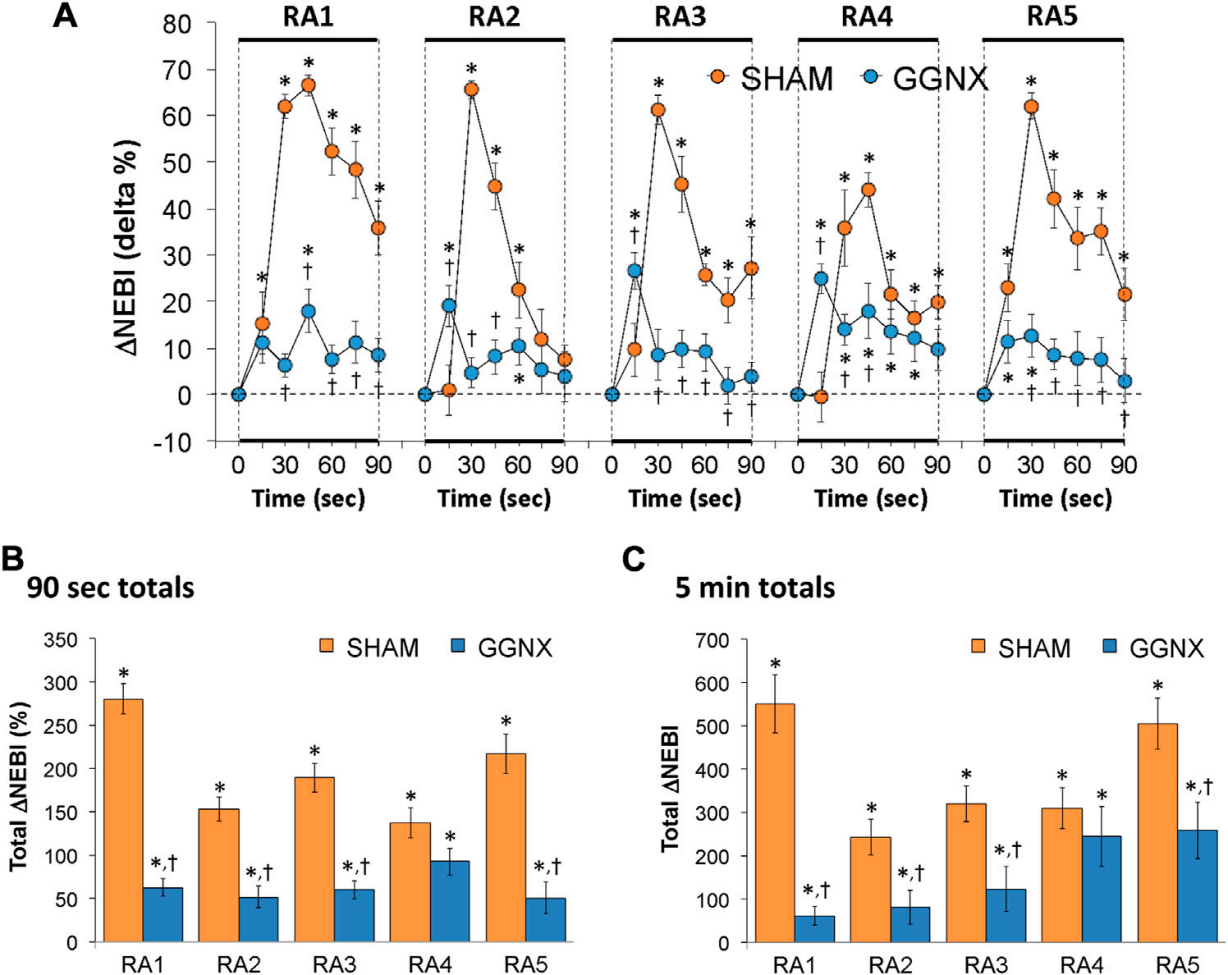
**(A)** Arithmetic changes in non-eupneic breathing index (NEBI) from Pre-values in sham-operated (SHAM) rats and in rats with bilateral ganglioglomerular nerve transection (GGNX) during the first 90 s upon return to room-air (RA1-RA5), following the 5 hypoxic (HX, 10% O_2_, 90% N_2_) gas challenges. **(B)** Total arithmetic changes in NEBI during the first 90 s of the return to room-air phases (RA1-RA5). **(C)** Total arithmetic changes in NEBI during the first 5 min of the return to room-air phases (RA1-RA5). The SHAM group had 10 rats. The GGNX group had 12 rats. The data are presented as mean ± SEM. *p < 0.05, significant response. ^†^ *p* < 0.05, GGNX rats *versus* SHAM rats.

NEBI/Freq values in SHAM and GGNX rats before and during five HXC (10% O_2_, 90% N_2_) each separated by 15 min of room-air are presented in Supplementary Figure S7A. Each HXC elicited minor increases in NEBI/Freq in SHAM and GGNX rats that rose upon return to room-air in SHAM rats, but not in GGNX rats. The initial (1–90 s) increase in NEBI/Freq during HXC 2-5 were greater in GGNX than in SHAM rats Supplementary Figure S7B, C. The total increase in NEBI/Freq recorded during the entire 5 min of HXC 1-5 in GGNX rats contrasted to the decrease in NEBI/Freq (HXC 1 to HXC 5) seen in the SHAM rats Supplementary Figure S7D. The ventilatory responses that occurred upon return to room-air will be detailed in the next section, but special mention is made of the changes in NEBI. The initial (1–90 s) arithmetic increases in NEBI upon return to room-air following HXC 1–5 (RA1-RA5) are shown in Figure 15A. The rapid and substantial increase in NEBI observed in SHAM rats was largely absent in the GGNX rats. The total increase in NEBI that occurred during the initial 90 s Figure 15B and first 5 min Figure 15C of RA-1-RA5 were markedly smaller in GGNX rats than in SHAM rats.

### Return to room-air responses (RA1-RA5)

#### Freq, TV and MV responses

The arithmetic changes Freq, TV and MV that occurred in the first 90 s following return to room-air after each HXC (i.e., RA1–RA5) are summarized in each of Supplementary Figures S8A, S9A, S10A, respectively. The return to room-air elicited generally transient increases in Freq, TV and MV. The increases in Freq tended to be smaller in GGNX rats, whereas the increases in TV were larger in the GGNX rats than the SHAM rats, which together resulted in similar increases in MV in the two groups. As shown in each of Supplementary Figures S8B, S9B, S10B, the total increases in Freq that occurred over the first 90 s of RA1 and RA2 were smaller in GGNX rats, whereas the total increases in TV in GGNX rats for RA1-RA5 were markedly different from the decreased responses seen in SHAM rats. As a result, the increases in MV were similar between SHAM and GGNX rats except for RA4 in which the increase in MV was greater in GGNX rats.

#### Ti, Te and Te/Ti responses

The arithmetic changes Ti, Te and Te/Ti that occurred over the first 90 s upon return to room air after each HXC (RA1–RA5) are summarized in each of Supplementary Figures S11A, S12A, S13A **,** respectively. The return to room-air elicited decreases in Ti, initial decreases followed by increases in Te, and increases in Te/Ti. The changes in Ti, Te and Te/Ti were most often similar in the SHAM and GGNX rats and this was reflected in the total changes in Ti, Te and Te/Ti during the first 90 s Supplementary Figures S11B, S12B, S13B or first 5 min of return to room-air Supplementary Figures S11C, S12C, S13C.

#### EIP and EEP responses

The arithmetic changes in EIP and EEP that occurred over the first 90 s following return to room-air after each HXC (RA1-RA5) are summarized in each of Supplementary Figures S14A and S15A, respectively. The return to room-air elicited decreases in EIP that tended to be greater in the GGNX rats, and increases in EEP that were substantially greater in the GGNX rats. The total decreases in EIP during the first 90 s Supplementary Figure S14B or first 5 min Supplementary Figure S14C were substantially greater in the GGNX rats than in the SHAM rats. The total increases in EEP during the first 90 s Supplementary Figures S15B or first 5 min Supplementary Figures S15C were substantially greater in the GGNX rats than in the SHAM rats.

#### PIF, PEF and PEF/PIF responses

The arithmetic changes in PIF, PEF and PEF/PIF ratio that occurred over the first 90 s following return to room-air after each HXC (RA1-RA5) are shown in each Supplementary Figures S16, S17, S18 **,** respectively. As can be seen in Supplementary Figure S16, the return to room-air elicited increases in PIF that were similar in magnitude in SHAM and GGNX rats Supplementary Figure S16A, and the total responses recorded over 90 s and 5 min were also overall similar between the groups Supplementary Figure S16B, C. As seen in Supplementary Figure S17, the return to room-air elicited increases in PEF that were greater in GGNX rats compared to SHAM rats recorded over 90 s and 5 min Supplementary Figures S17A–C. As a result, the increases in PEF/PIF (Supplementary Figure S18) were larger in the GGNX rats compared to the SHAM rats for RA2-RA5 recorded over 90 s and 5 min (Supplementary Figures S18B, C).

#### EF_50_ responses

The arithmetic changes in EF_50_ that occurred over the first 90 s following return to room-air after each HXC (RA1-RA5) are shown in Supplementary Figure S19. Upon return to room-air, EF_50_ values rose initially and then fell below baseline values in a similar trend in both the SHAM and GGNX groups Supplementary Figure S19A such that the total changes during the first 90 s Supplementary Figure S19B and 5 min Supplementary Figure S19C were in general similar in both groups.

#### Relaxation time responses

The arithmetic changes in relaxation time that occurred over the first 90 s after return to room-air after each HXC (RA1-RA5) are shown in Supplementary Figure S20. Upon return to room-air, relaxation time values fell initially and then often rose above baseline values in a similar trend in the SHAM and GGNX groups Supplementary Figure S20A such that the total changes during the first 90 s Supplementary Figure S20B and 5 min Supplementary Figure S20C were in general similar in the two groups, except for those of RA1, in which the total decreases in relaxation time seen in the SHAM rats were markedly less over the first 90 s and converted to a net increase over the first 5 min in the GGNX rats.

#### Apneic pause responses

The arithmetic changes in apneic pause values that occurred over the first 90 s following return to room-air after each HXC (RA1-RA5) are shown in Supplementary Figure S21. Upon return to room-air, apneic pause values gradually rose above baseline values in a similar trend in the SHAM and GGNX rats Supplementary Figure S21A such that the total changes during the first 90 s Supplementary Figure S21B and the first 5 min Supplementary Figure S21C were in general similar in the two groups.

#### Inspiratory drive responses

Arithmetic changes in inspiratory drive values that occurred over the first 90 s upon return to room-air after each HXC (RA1-RA5) are shown in Supplementary Figure S22. Upon return to room-air, inspiratory drive values initially rose above baseline values and then mostly returned toward baseline values in a similar fashion in the SHAM and GGNX rats Supplementary Figure S22A such that the total changes during the first 90 s Supplementary Figure S22B and the first 5 min Supplementary Figure S22C were in general similar in the two groups, except for RA3 and RA4 during which the increases were greater in the GGNX rats. It is noted the the increases from baseline in inspiratory drive observed during the 5 min period for RA5 being significantly smaller in the GGNX rats compared to the SHAM rats (Supplementary Figure S22C).

#### Expiratory drive responses

The arithmetic changes in expiratory drive values that occurred over the first 90 s following return to room-air after each HXC (RA1-RA5) are shown in Supplementary Figure S23. Upon return to room-air, expiratory drive values initially rose above baseline values and then returned toward and sometimes below baseline values in a similar trend in the SHAM and GGNX rats Supplementary Figure S23A such that the total changes during the first 90 s Supplementary Figure S23B and the first 5 min Supplementary Figure S23C were in general similar in the two groups, except for RA1 during which the increases were significantly smaller in GGNX rats.

#### NEBI/Freq responses

The arithmetic changes in NEBI/Freq values that occurred over the first 90 s following return to room-air after each HXC (RA1-RA5) are shown in Supplementary Figure S24. Upon return to room-air, NEBI/Freq values gradually rose to levels substantially above baseline values in SHAM rats, whereas these increases were in general smaller in GGNX rats Supplementary Figure S24A. As such, the total changes during the first 90 s Supplementary Figure S24B and the first 5 min Supplementary Figure S24C were smaller in GGNX than in SHAM rats.

## Discussion

There is wide-spread agreement that post-ganglionic sympathetic nerves provide extensive innervation of the vasculature within the carotid body (McDonald and Mitchell, 1975; Ichikawa, 2002). The majority of evidence suggests that there are 2 subtypes of primary glomus cells in rats, known as type 1A and type 1B. Type 1A and type 1B primary glomus cells do not receive post-ganglionic sympathetic innervation, however, type 1A, but not type 1B glomus cells, receive innervation from petrosal ganglion chemoafferents (McDonald and Mitchell, 1975; McDonald, 1983a; McDonald, 1983b). There is no evidence that sustentacular (type 2) glomus cells receive afferent or efferent innervation (McDonald and Mitchell, 1975; McDonald, 1983a; McDonald, 1983b; Ichikawa, 2002). On the basis of the substantial evidence that GGNs innervate their ipsilateral carotid bodies (Biscoe and Purves, 1967; Zapata et al., 1969; Bowers and Zigmond, 1979; Brattström, 1981a; McDonald and Mitchell, 1981; McDonald, 1983a; McDonald, 1983b; Verna et al., 1984; Torrealba and Claps, 1988; Ichikawa, 2002; Asamoto, 2004; Savastano et al., 2010), we are working on the key assumption that bilateral transection of the GGN (GGNX) leads to alterations in the functions of primary (type 1) glomus cells and vasculature, and perhaps sustenacular (type 2) glomus cells and/or chemoafferent nerve terminals. A decrease in GGN input to the carotid bodies may result from a loss of function of pre-ganglion neurons and/or post-ganglionic cell bodies in the SCG as a result of physical insults and/or disease processes including inflammatory diseases (Rudik, 1969; Camargos and Machado, 1988; Laudanna et al., 1998; Hanani et al., 2010), prion diseases (Liberski, 2019), herpes simplex virus infection (Price and Schmitz, 1979), acquired immunodeficiency syndrome (Chimelli et al., 2002), metastatic states (Moubayed et al., 2017), hyperthyroidism (Matano et al., 2014); amyotrophic lateral sclerosis (Kandinov et al., 2013), neuro-endocrine disorders in females (Pirard, 1954); myocardial ischemia (Liu et al., 2013; Cheng et al., 2018), obstructive jaundice (Chen et al., 2021); Duchenne muscular dystrophy (De Stefano et al., 2005), Alzheimer’s disease (Jengeleski et al., 1989; Alzoubi et al., 2011), amyloid precursor protein deficiency (Cai et al., 2016), Lewy body disease and Parkinson’s disease (Del Tredici et al., 2010), Mecp2 deficiency (Roux et al., 2008), lead exposure (Zhu et al., 2019), hypercholesterolemia Chumasov et al., 1994), hypertension (Tang et al., 1995a; Tang et al., 1995b; Tang et al., 1995c), direct ischemic challenge (Kilic et al., 2019), multiple systems neuropathy (McGorum et al., 2015), diabetic neuropathy (Minker et al., 1978; Bitar et al., 1997; Cameron and Cotter, 2001; Li et al., 2017), small fiber neuropathies (Han et al., 2012) and damage to peripheral axons in the CSC (Shin et al., 2014; Niemi et al., 2017). Moreover, a loss of activity of post-ganglionic SCG neurons could results from damage to T1-T4 regions of the spinal cord which contain pre-ganglionic cell bodies that innervate the SCG (Rando et al., 1981; McDonald, 1983a; McDonald, 1983b; Tang et al., 1995a; Tang et al., 1995b; Tang et al., 1995c; Llewellyn-Smith et al., 1998). Such thoracic spinal cord damage causes an array of ventilatory impairments (Takeda et al., 1977; Sugarman, 1985; Oku et al., 1997; Roth et al., 1997; Ayas et al., 1999; Forster, 2003; Bolser et al., 2009; Schilero et al., 2009; Bascom et al., 2016; Berlowitz, et al., 2016; Hachmann et al., 2017), as well as sleep disordered breathing (Braun et al., 1982; Sankari et al., 2014a; Sankari et al., 2014b; Bascom et al., 2015; Sankari et al., 2019).

It may be expected that removal of the GGNX input to the carotid bodies will lead to changes in glomus cell-chemoafferent nerve activity that result in altered baseline ventilatory status. Previous evidence has shown that an increase in activity of the carotid body-carotid sinus nerve complex leads to increases in Freq, TV and MV (Eldridge, 1972; Eldridge, 1976; Eldridge, 1978; Marek et al., 1985; Engwall et al., 1991; Katayama et al., 2019). In contrast, silencing of the carotid body complex, such as under hyperoxic challenge, has a relatively minor but distinct decrease in Freq, although variable effects on TV and therefore MV have been reported (Palecek and Chválová, 1976; Cardenas and Zapata, 1983; Arieli, 1994; Strohl et al., 1997; Nakano et al., 2002; Souza et al., 2018). As such, key findings of the present study were that although most resting ventilatory values was similar in SHAM and GGNX rats, resting Freq was lower in GGNX rats than in the SHAM rats and this was associated with higher Te and EEP values in GGNX rats than in SHAM rats. These findings certainly suggest that the loss of GGNX input to the carotid body complex diminished carotid body activity. Since this decrease in Freq was expressed under room-air in quietly resting rats, it is unlikely that the decrease in Freq was caused indirectly by increases in blood flow to the carotid body microvasculature since an increase in blood flow as a result of vasodilation due to the loss of GGN vasoconstrictor input is unlikely to change oxygenation status under resting/normoxic states. Contrarily, a decrease in blood flow would indirectly activate carotid body glomus cells (Llados and Zapata, 1978; Majcherczyk et al., 1980; Matsumoto et al., 1981; Yokoyama et al., 2015) to promote Freq. As such, it would be tempting to assume that the loss of GGNX input to carotid body has altered signaling processes in primary glomus cells that leads to diminished neurotransmitter release and less activation of chemoafferent nerve terminals. Thus, the presumed decrease in carotid body function should mimic the changes in resting ventilatory parameters seen in rats with bilateral carotid sinus nerve (CSN) transection (CSNX). Supplementary Table S3 provides a qualitative assessment of the status of resting (Pre-challenge) ventilatory parameters in GGNX and CSNX rats compared to their respective sham-operated (SHAM) controls with the original Freq, TV and MV data for the CSNX rats coming from Getsy et al. (2020) and the remaining original unpublished data for the variables described for the CSNX rats. As can be seen, resting Freq was lower than the SHAM in GGNX and CSNX rats. Although resting TV was not diminished in GGNX or CSNX rats, the combined changes in Freq and TV resulted in a decrease in resting MV in CSNX, but not GGNX rats. The decreases in resting Freq in GGNX and CSNX rats were associated with increases in Te, but not changes in Ti. Other notable differences between GGNX and CSNX rats were that resting EEP, PEF/PIF were elevated, whereas NEBI was decreased in GGNX but not CSNX rats, and PEF and inspiratory drive were decreased, whereas NEBI/Freq was elevated in CSNX but not GGNX rats. It should be noted that resting values for Te, Te/Ti, EF_50_, relaxation time, apneic pause and expiratory drive were similar in GGNX and CSNX rats to those of their respective SHAM controls. Overall, it appears that GGNX elicits changes in carotid body function that mimic some but not all aspects of CSNX on resting ventilatory parameters. We are currently performing RNAseq and other studies to uncover the molecular changes that may result from GGNX.

It is important to note that whereas the increases in Freq, TV and MV elicited by a 5 min HX challenge were certainly diminished in juvenile (P25) with bilateral CSNX, substantial responses still remained. Accordingly, other yet to be determined HX-sensitive, but carotid body-independent processes, that would not necessarily be under the control of the GGN input to the carotid bodies must exist (Getsy et al., 2020; Getsy et al., 2021a; Getsy et al., 2021b). This is important information that will hopefully help to frame our understanding of how GGNX modulates the HX-induced changes in ventilation. Despite being separated by 15 min of room-air, there were reactively modest, but clear, gradual diminutions in the total changes in many of the ventilatory parameters during the later HX challenges compared to the initial HX challenges. Whether this diminution in the later HX response reflects a true adaptation of carotid body-central signaling pathways or is due to some other internal (e.g., behavioral) adaptation remains to be determined. The ventilatory responses elicited by the five HX challenges in juvenile (P25) Sprague-Dawley SHAM rats included increases in Freq in which a substantial roll-off during the 5 min challenges only occurred during HX1. These increases in Freq were associated with decreases in Ti, Te and EIP, but minor changes in EEP. Why HX challenges were able to decrease the pause at the end of inspiration so that expiration started more rapidly instead of decreasing the pause at the end of expiration so that inspiration began as it would under normoxic states, is an unresolved question, but suggests that carotid body chemoafferent input to the brain preferentially controls the processes that regulate EIP. The HX1-HX5 challenges were associated with substantial increases in TV each of which displayed distinct roll-off during the 5 min HX challenges. Taken together, these changes in Freq and TV resulted in robust increases in MV. The HX challenges elicited robust increases in PIF, PEF, PEF/PIF and EF_50_ of which all except PIF displayed roll-off. The HX challenges also elicited robust increases in both inspiratory drive and expiratory drive with the increases in expiratory drive only displaying substantial roll-off. The above responses were associated with decreases in relaxation time that did not show roll-off and minor decreases in apneic pause that did not display roll-off. Importantly, we observed a substantial increase in NEBI during HX1, but minor changes in NEBI during HX2-HX5 and a minor increase in NEBI/Freq during HX1, but substantial decreases in NEBI/Freq during HX2-HX5. The later observations related to NEBI and Freq clearly suggest that the rats processed the first HX challenge differently from other challenges. Again, whether this represents adaptations in ventilatory signaling, changes in internal (behavioral) response to the HX challenges or interactions between these processes is an interesting unresolved issue. Moreover, we have no explanations as to why some of the ventilatory responses show roll-off, whereas others do not. However, it would seem that adaptations within the carotid bodies and resulting chemoafferent signals to the brain would result in the presence of or the lack of roll-off in every parameter. It would therefore seem that the presence or absence of roll-off is due to central processing unique to each parameter.

With respect to the ventilatory responses elicited by the five HX challenges, it is clear that the differences in responses between the GGNX and SHAM rats were much more evident with the second through fifth HX challenges (HX2-HX5) than with the first HX challenge (HX1). As summarized in Supplementary Table S4, this group difference was most evident for Freq, MV, Ti, PIF, PEF, PEF/PIF, EF_50_ and inspiratory drive whereas it was not as noticeable for TV, Te, Te/Ti, EIP, EEP, relaxation time, expiratory drive, apneic pause, NEBI or NEBI/Freq. We have no definitive explanation for these findings, but believe it possible that the first HX challenge may be able to release and deplete residual catecholamine stores in sympathetic terminals of transected GGN nerves such that the ventilatory responses elicited by HX2-HX5 in GGNX rats represent the actual status of the carotid body complex. Such residual stores have been found in a variety of adult animals (Edvinsson et al., 1975; Dowell, 1976; Priola et al., 1981; Eisenach et al., 2002; Imrich et al., 2009) and in juvenile (P32) rats (Smith, 1986). Why the differences in HX-induced responses between SHAM and GGNX, such as the increases in Freq, are clearly more evident for episodes HX2-HX5 than HX1, whereas the differences in other responses (e.g., TV) between GGNX and SHAM rats during HX1-HX5 challenges are similar to one another is another perplexing question. A consistent observation and most noticeable during HX2-HX5 was that the initial rates of response during the HX challenges were often slower in GGNX rats than in SHAM rats (e.g., Freq, TV, MV, PIF, PEF, EF_50_, and inspiratory and expiratory drives), although similar plateau levels were usually obtained in both groups. This key finding tentatively suggests that the mechanisms responsible for the initial ventilatory responses to HX challenge are different to those that maintain these responses and that the mechanisms responsible for the initial responses are substantially downregulated in the absence of GGN input. Obviously a plethora of known and suspected functional proteins/signaling pathways within the carotid bodies and brain could be involved (Lahiri et al., 2006; Teppema and Dahan, 2010; Prabhakar, 2013; Semenza and Prabhakar, 2015). A rather unique observation was that EEP rose gradually and substantially during each HX challenge in the GGNX rats, whereas EEP did not change during HX1-HX5 in the SHAM rats. As such, we can conclude that under normal circumstances, input to the brainstem from the carotid body chemofferents prevents the delay in switching from expiration to inspiration.

### Room-air responses

The return to room-air following the HX challenges resulted in a series of ventilatory responses in the juvenile rats. The substantial changes in breathing that occurs upon return to room-air is consistent with our findings that returning to room-air following HX, hypercapnic or hypoxic hypercapnic-gas challenges results in a series of ventilatory responses in juvenile Sprague-Dawly rats (Getsy et al., 2020), adult Sprague-Dawley rats (May et al., 2013a; May et al., 2013b; Gaston et al., 2020) and adult C57BL6 mice (Palmer et al., 2013a; Palmer et al., 2013b; Gaston et al., 2014; Getsy et al., 2014; Getsy et al., 2021a; Getsy et al., 2021b; Getsy et al., 2021c; Getsy et al., 2021d; Getsy et al., 2021e). Moreover, the return to room-air responses were virtually absent in juvenile rats (Getsy et al., 2020), adult rats (Gaston et al., 2020) and in adult mice (Getsy et al., 2021c) with bilateral CSN transection and were markedly smaller in adult mice lacking endothelial nitric oxide synthase (Getsy et al., 2021d; Getsy et al., 2021e) or hemoglobin beta-93-cysteine (Gaston et al., 2014), whereas the ventilatory responses were greater in adult mice lacking S-nitrosoglutathione reductase (Palmer et al., 2013b). Our working hypotheses are that the return to room-air responses are vitally dependent upon the generation of S-nitrosothiols in red blood cells that act in the carotid body complex, and that activation of chemosensory afferents is the vital signaling mechanism in the expression of the return to room-air ventilatory responses (Gaston et al., 2014, 2020; Getsy et al., 2020; Getsy et al., 2021c). Since the qualitative and/or quantitative nature of many of the return to room-air responses after HX challenges were markedly different in mice with bilateral CSCX (Getsy et al., 2021a) or bilateral SCGX (Getsy et al., 2021b) compared to their SHAM controls, we hypothesize that GGN input to the carotid body is essential for maintaining signaling mechanisms responsible for the return to room-air responses.

In SHAM rats, Freq rose rapidly upon return to room-air and returned to baseline levels within 2 min during RA1. The elevations in Freq upon return to room-air were progressively smaller for RA2-RA5 and returned much more quickly to baseline levels during RA2-RA5. TV rose immediately upon return to room-air during RA1-RA5 and then dropped rapidly toward baseline. As such MV rose initially upon return to room-air and returned to baseline levels within 2–3 min during RA1 and more rapidly during RA2-RA5. Ti fell during RA1 and gradually returned to baseline levels within 2 min. However, after a brief fall, Te rose to well above baseline before recovering by 5 min. Te/Ti rose remarkably during RA1-RA5 before returning to baseline within 3–4 min. PIF and PEF rose during RA1-RA5 and returned toward baseline within 90–120 s, such that PEF/PIF values did not change appreciably overall although there were several instances when PEF/PIF rose to about the 60–90 s time-points. EF_50_ rose noticeably during RA1, but rose progressively less during RA2-RA5 before returning to or falling below baseline levels. Relaxation time fell substantially during RA1 before recovering to baseline by 120 s. Relaxation time during RA2-RA5 fell only minimally before rising above baseline levels. Apneic pause values changed minimally during RA1-RA5. Inspiratory drive values increased during RA1-RA5 before returning to baseline values within 90–120 s. Expiratory drive values increased during RA1-RA5 before returning to or falling baseline (RA2-RA5) within 30–60 s. NEBI and NEBI/Freq rose markedly during RA1-RA5 and fell gradually toward baseline within 90–150 s. This remarkable set of responses was changed substantially in GGNX rats. The qualitative changes in ventilatory responses during RA1-RA5 in GGNX rats compared to SHAM rats are summarized in Supplementary Table S5. The increase in Freq seen during RA1 and RA2 in SHAM rats was diminished in GGNX rats. The decreases in TV observed during the first 90–120 s of RA1-RA5 in SHAM rats were converted to increases in TV in the GGNX rats. As such, the increase in MV during RA1 was smaller in GGNX rats compared to SHAM rats, whereas the increase in MV during RA4 was greater in GGNX rats. The decreases in Ti in GGNX rats were largely similar to those in SHAM rats, however the decreases in Te in RA1 and RA2 in SHAM rats were absent or converted to an increase in Te, respectively, in the GGNX rats. As such, the increase in Te/Ti during RA1 was substantially greater in the GGNX rats. The substantial falls in EIP seen during RA1-RA5 in SHAM rats were greater in GGNX rats, whereas the substantial rises in EEP seen during RA1-RA5 in SHAM rats were greater in GGNX rats. The increases in PIF seen in SHAM rats were somewhat augmented in GGNX rats, whereas the increases in PEF observed in SHAM rats were substantially augmented in GGNX rats, such that the changes in PEF/PIF were greatly enhanced in the GGNX rats. The changes in EF_50_ observed in the SHAM rats during episodes RA1-RA3 were dramatically altered in GGNX rats, whereas the pronounced decrease in relaxation time seen in the SHAM rats during HX1 was reversed to an increase in GGNX rats. The increases in apneic pause, inspiratory drive and expiratory drive observed in the SHAM rats were similar in the GGNX rats except for a notably smaller increase in expiratory drive in the GGNX rats during RA1. Of major interest with respect to the status of breathing patterns were the findings that the RA1-RA5 increases in NEBI and NEBI/Freq were remarkably reduced in the GGNX rats. Taken together, these data strongly suggest that GGNX induces functional changes within the carotid body complex. The precise nature of these changes and the subtypes of structures within the carotid body in which these changes occur remain to be determined. We have reported that return to room-air following HX challenge elicits pronounced changes in ventilatory parameters in naïve C57BL6 mice that are associated with only minor behavioral responses (Getsy et al., 2014). Similarly, the SHAM and GGNX rats did not display any overt changes in behavior (e.g., movement about the chamber, grooming, rearing, paw licking) upon return to room-air. As such, it is possible that the differences in ventilatory responses seen upon return to room-air in the present study reflects true differences in ventilatory signaling between the SHAM and GGNX rats.

## Study limitations

There are several important limitations of this study. The first was that this study was performed in juvenile (P25) male rats only and studies in juvenile (P25) female rats and in adult (e.g., P100) male and female rats are now certainly warranted. Juvenile rats at P21 were chosen for these studies because the survival surgeries can be successfully performed and then after full recovery, the rats at age P25 can be studied. Additionally P25 is the optimal age that allows electrophysiological studies to be done in brainstems from these rats (Getsy et al., 2019). Another important limitation is that the testing/recording sessions were performed 4 days post-surgery and obviously it will be important to test the SHAM and GGNX rats at later time-points to see whether the presumed changes in signaling mechanisms within the carotid bodies changes over time. Moreover, we need to gather information as to the precise nature of the structural, biochemical and cell-signaling changes that may occur in the subtypes of structures (e.g., primary glomus cells, sustenacular cells, nerve terminals of chemoafferents and vasculature) in the carotid bodies. A final limitation is the lack of evidence as to whether the changes to hypercapnic or hypoxic-hypercapnic gas challenges that we have established to elicit robust ventilatory responses in juvenile P25 rats (Getsy et al., 2020) are modulated in male and female GGNX rats. Since resting ventilatory parameters were similar in the SHAM and GGNX mice, we expect that resting arterial blood chemistry (ABG) values (e.g., pH, pCO_2_, pO_2_ and sO_2_) would have been similar in the two groups. We are preparing to address the vital question as to how the ABG chemistry values change during HX challenge in these rats to gain a better understanding of how GGNX affects ventilatory performance. Moreover, the key issue as to whether ventilatory responses to hypercapnic gas challenges will be different in GGNX rats will add greatly to our understanding of how the loss of GGN input to the carotid bodies affects ventilatory signaling. Additionally, a more in-depth evaluation of the true effects of GGNX on ventilatory signaling must await studies in which the rats are challenged with progressively greater HX challenges (e.g., 18%–15% to 12%–10% O_2_), and the data pertinent to carotid body function analyzed by exponential curve analyses. Furthermore, the precise effects of GGNX on carotid body function would be further clarified in studies in which the changes in magnitude and gain of the carotid body-mediated chemoreflex during HX challenge were evaluated.

## Conclusion

Our data demonstrate that bilateral removal of the GGN post-ganglionic sympathetic input to the carotid bodies has a dramatic effect on the changes in ventilatory function that occur during and following HX gas challenges in juvenile rats. There is substantial evidence that the GGN project to various structures in the carotid bodies and to the carotid sinus to regulate the functions of primary glomus cells, chemoafferents, vasculature (Biscoe and Purves, 1967; Zapata et al., 1969; Bowers and Zigmond, 1979; Brattström, 1981a; McDonald and Mitchell, 1981; McDonald, 1983a; McDonald, 1983b; Verna et al., 1984; Torrealba and Claps, 1988; Ichikawa, 2002; Asamoto, 2004; Savastano et al., 2010) and baroreceptor afferents (Floyd and Neil, 1952; Rees, 1967; Bolter and Ledsome, 1976; Brattström, 1981b; Felder et al., 1983; Buller and Bolter, 1993). It is tempting to assume that most of the changes in ventilatory function in the GGNX rats are therefore due to adaptive changes in the carotid bodies, although changes in baroreceptor afferent input to the brainstem may also play a role. The rather startling changes in the responses of particular ventilatory parameters in GGNX rats, such as EEP and NEBI, both during the HX challenges and upon return to room-air, provide us with deeper insights into the possible mechanisms by which carotid body chemoafferent input regulates ventilatory parameters, and how aberrant changes in this input affects breathing. Additionally, it is important to note that the SCG contains small intensely fluorescent (SIF) cells that are innervated by spinal pre-ganglionic sympathetic nerves and glossopharyngeal sensory nerves endings whose cell bodies reside in the petrosal ganglia (Takaki et al., 2015). Neurotransmitters/neuromodulators released from SIF cells modulate the activity of pre-and post-ganglionic neurons within the SCG (Tanaka and Chiba, 1996) and appear to play a role in the upregulation of norepinephrine synthesis in SCG post-ganglionic neurons in response to hypoxia (Brokaw and Hansen, 1987). This raises the intriguing possibility that SIF cells play an important role in regulating the activity of SCG cells that project through the GGN to innervate the carotid bodies (Verna et al., 1984; Brognara et al., 2021). Our data provides a basis for planning studies in which implantation of stimulating devices in the spinal cord or upon the CSC-SCG complex may be of therapeutic benefit. The potential challenges of spinal cord and autonomic nerve stimulation approaches to restore ventilatory function has been addressed and many hurdles still remain (Hachmann et al., 2017).

The fundamental question arising from this study pertains to how the loss of GGN input to CB structures, such as primary glomus cells, satellite cells, chemoafferent nerve terminals and vasculature (Brognara et al., 2021), results in altered functional responses of the carotid body to HX challenges. We hypothesize that the loss of GGN input alters the expression of functional proteins (Mulligan et al., 1981; Prabhakar, 1999; Lahiri et al., 2006; Prabhakar, 2013; Prabhakar and Semenza, 2015) including those that generate catecholamines (Mir et al., 1982; Pequignot et al., 1991), which play vital roles in mediating the ventilatory responses to HX challenges. While it is evident the early ventilatory responses to HX challenge were markedly different in GGNX rats than in SHAM rats, it was obvious that the maximal responses of the two groups were similar after about 90–120 s. As such, it is evident that compensatory mechanisms within the carotid body chemosensitive glomus cells, and perhaps efferent and afferent neurons associated with the carotid bodies, come into play as the HX challenge progresses. We can only speculate as to what these mechanisms are, but it is intriguing to consider that they may involve alterations in the expression of functional plasma membrane proteins and/or gradual rises in the influence of mitochondrial-dependent mechanisms as ATP levels are gradually depleted during hypoxia exposure (Mulligan et al., 1981; Prabhakar, 1999; Lahiri et al., 2006; Prabhakar, 2013; Prabhakar and Semenza, 2015).

## Data Availability

The raw data supporting the conclusion of this article will be made available by the authors, without undue reservation.

## Supplementary material

SUPPLEMENTARY FIGURE S1 Relationships between peak inspiratory flow (PIF), peak expiratory flow (PEF), relaxation time (RT) and expiratory time (Te).

SUPPLEMENTARY FIGURE S2
**(A)** Expiratory time/Inspiratory time (Exp time/Insp time or Expir Time/Inspir Time) in sham-operated (SHAM) rats and in rats with bilateral ganglioglomerular nerve transection (GGNX) before (Pre) and during five hypoxic (HX, 10% O_2_, 90% N_2_) gas challenges, each separated by 15 min of room-air (RA). **(B)** Arithmetic changes in expiratory time/Arithmetic changes in inspiratory time ((ΔTe (sec)/(ΔTi (sec)) during the first 90 s of HX gas challenge. **(C)** Total changes in expiratory time/total changes in inspiratory time ((Total ΔTe (sec)/(Total ΔTi (sec)) during the first 90 s of the HX gas challenge. **(D)** Total changes in expiratory time/total changes in inspiratory time ((Total ΔTe (sec)/(Total ΔTi (sec)) during the entire 5 min of the HX gas challenge. The SHAM group had 10 rats. The GGNX group had 12 rats. Data are presented as mean ± SEM. **p* < 0.05, significant response. ^†^ *p* < 0.05, GGNX rats versus SHAM rats.

SUPPLEMENTARY FIGURE S3
**(A)** End Inspiratory Pause (EIP or End Insp Pause) in sham-operated (SHAM) rats and in rats with bilateral ganglioglomerular nerve transection (GGNX) before (Pre) and during five hypoxic (HX, 10% O_2_, 90% N_2_) gas challenges, each separated by 15 min of room-air (RA). **(B)** Arithmetic changes in EIP (ΔEnd Insp Pause) during the first 90 s of HX gas challenge. **(C)** Total changes in end inspiratory pause (Total ΔEIP) during the first 90 s of HX gas challenge. **(D)** Total changes in end inspiratory pause (Total ΔEIP) during the entire 5 min of HX gas challenge. The SHAM group had 10 rats. The GGNX group had 12 rats. The data are presented as mean ± SEM. **p* < 0.05, significant response. ^†^ *p* < 0.05, GGNX rats versus SHAM rats.

SUPPLEMENTARY FIGURE S4
**(A)** Peak expiratory flow/peak inspiratory flow (PEF/PIF) ratios in sham-operated (SHAM) rats and in rats with bilateral ganglioglomerular nerve transection (GGNX) before (Pre) and during five hypoxic (HX, 10% O_2_, 90% N_2_) gas challenges, each separated by 15 min room-air (RA). **(B)** Arithmetic changes in PEF/PIF during the first 90 s of HX gas challenge. **(C)** Total changes in PEF/PIF during the first 90 s of HX gas challenge. **(D)** Total changes in PEF/PIF during the entire 5 min HX gas challenge. The SHAM group had 10 rats. The GGNX group had 12 rats. Data are presented as mean ± SEM. **p* < 0.05, significant response. ^†^ *p* < 0.05, GGNX rats versus SHAM rats.

SUPPLEMENTARY FIGURE S5
**(A)** Relaxation time in sham-operated (SHAM) rats and in rats with bilateral ganglioglomerular nerve transection (GGNX) before (Pre) and during five hypoxic (HX, 10% O_2_, 90% N_2_) gas challenges, each separated by 15 min of room-air (RA). **(B)** Arithmetic changes in relaxation time during the first 90 s of HX gas challenge. **(C)** Total changes in relaxation time during the first 90 s and **(D)** over the entire 5 min of HX gas challenge. The SHAM group had 10 rats. The GGNX group had 12 rats. The data are presented as mean ± SEM. **p* < 0.05, significant response. ^†^ *p* < 0.05, GGNX rats versus SHAM rats.

SUPPLEMENTARY FIGURE S6
**(A)** Apneic pause values in sham-operated (SHAM) rats and in rats with bilateral ganglioglomerular nerve transection (GGNX) before (Pre) and during five hypoxic (HX, 10% O_2_, 90% N_2_) gas challenges, each separated by 15 min of room-air (RA). **(B)** Arithmetic changes in apneic pause during the first 90 s of HX gas challenge. Total changes in apneic pause during the first 90 s **(C)** and over the entire 5 min **(D)** of HX gas challenge. The SHAM group had 10 rats. The GGNX group had 12 rats. The data are presented as mean ± SEM. **p* < 0.05, significant response. ^†^ *p* < 0.05, GGNX rats versus SHAM rats.

SUPPLEMENTARY FIGURE S7
**(A)** Non-eupneic breathing index (NEBI)/Frequency of breathing ratios in sham-operated (SHAM) rats and in rats with bilateral ganglioglomerular nerve transection (GGNX) before (Pre) and during five hypoxic (HX, 10% O_2_, 90% N_2_) gas challenges, each separated by 15 min of room-air (RA). **(B)** Arithmetic changes in frequency during the first 90 s of HX gas challenge. Total changes in frequency during the first 90 s **(C)** and over the entire 5 min **(D)** of HX gas challenge. The SHAM group had 10 rats. The GGNX group had 12 rats. The data are presented as mean ± SEM. **p* < 0.05, significant response. ^†^ *p* < 0.05, GGNX rats versus SHAM rats.

SUPPLEMENTARY FIGURE S8
**(A)** Arithmetic changes in frequency (Freq) of breathing from Pre-values in sham-operated (SHAM) rats and in rats with bilateral ganglioglomerular nerve transection (GGNX) during the first 90 s upon return to room-air (RA1-RA5), following the five hypoxic (HX, 10% O_2_, 90% N_2_) gas challenges. **(B)** Total arithmetic changes in Freq (ΔFreq) during the first 90 s of the return to room-air phases (RA1-RA5). **(C)** Total arithmetic changes in Freq (ΔFreq) during the first 5 min of the return to room-air phases (RA1-RA5). The SHAM group had 10 rats. The GGNX group had 12 rats. The data are presented as mean ± SEM. **p* < 0.05, significant response. ^†^ *p* < 0.05, GGNX rats versus SHAM rats.

SUPPLEMENTARY FIGURE S9
**(A)** Arithmetic changes in tidal volume from Pre-values in sham-operated (SHAM) rats and in rats with bilateral ganglioglomerular nerve transection (GGNX) during the first 90 s upon return to room-air (RA1-RA5), following the five hypoxic (HX, 10% O_2_, 90% N_2_) gas challenges. **(B)** Total arithmetic changes in tidal volume during the first 90 s of the return to room-air phases (RA1-RA5). **(C)** Total arithmetic changes in tidal volume during the first 5 min of the return to room-air phases (RA1-RA5). The SHAM group had 10 rats. The GGNX group had 12 rats. The data are presented as mean ± SEM. **p* < 0.05, significant response. ^†^ *p* < 0.05, GGNX rats versus SHAM rats.

SUPPLEMENTARY FIGURE S10
**(A)** Arithmetic changes in minute ventilation (MV) from Pre-values in sham-operated (SHAM) rats and in rats with bilateral ganglioglomerular nerve transection (GGNX) during the first 90 s upon return to room-air (RA1-RA5), following the five hypoxic (HX, 10% O_2_, 90% N_2_) gas challenges. **(B)** Total arithmetic changes in MV (ΔMV) during the first 90 s of the return to room-air phases (RA1-RA5). **(C)** Total arithmetic changes in MV (ΔMV) during the first 5 min of the return to room-air phases (RA1-RA5). The SHAM group had 10 rats. The GGNX group had 12 rats. The data are presented as mean ± SEM. **p* < 0.05, significant response. ^†^ *p* < 0.05, GGNX rats versus SHAM rats.

SUPPLEMENTARY FIGURE S11
**(A)** Arithmetic changes in Inspiratory Time (Insp Time) from Pre-values in sham-operated (SHAM) rats and in rats with bilateral ganglioglomerular nerve transection (GGNX) during the first 90 s upon return to room-air (RA1-RA5), following the five hypoxic (HX, 10% O_2_, 90% N_2_) gas challenges. **(B)** Total arithmetic changes in Insp Time (ΔInsp Time) during the first 90 s of the return to room-air phases (RA1-RA5). **(C)** Total arithmetic changes in Insp Time (ΔInsp Time) during the first 5 min of the return to room-air phases (RA1-RA5). The SHAM group had 10 rats. The GGNX group had 12 rats. The data are presented as mean ± SEM. **p* < 0.05, significant response. ^†^ *p* < 0.05, GGNX rats versus SHAM rats.

SUPPLEMENTARY FIGURE S12
**(A)** Arithmetic changes in Expiratory Time (Exp Time) from Pre-values in sham-operated (SHAM) rats and in rats with bilateral ganglioglomerular nerve transection (GGNX) during the first 90 s upon return to room-air (RA1-RA5), following the five hypoxic (HX, 10% O_2_, 90% N_2_) gas challenges. **(B)** Total arithmetic changes in Exp Time (ΔExp Time) during the first 90 s of the return to room-air phases (RA1-RA5). **(C)** Total arithmetic changes in Exp Time (ΔExp Time) during the first 5 min of the return to room-air phases (RA1-RA5). The SHAM group had 10 rats. The GGNX group had 12 rats. The data are presented as mean ± SEM. **p* < 0.05, significant response. ^†^ *p* < 0.05, GGNX rats versus SHAM rats.

SUPPLEMENTARY FIGURE S13
**(A)** Arithmetic changes in Expiratory Time/Inspiratory Time (Exp Time/Insp Time, Te/Ti) from Pre-values in sham-operated (SHAM) rats and in rats with bilateral ganglioglomerular nerve transection (GGNX) during the first 90 s upon return to room-air (RA1-RA5), following the five hypoxic (HX, 10% O_2_, 90% N_2_) gas challenges. **(B)** Total arithmetic changes in Te/Ti (ΔTe/ΔTi) during the first 90 s of the return to room-air phases (RA1-RA5). **(C)** Total arithmetic changes in Te/Ti (ΔTe/ΔTi) during the first 5 min of the return to room-air phases (RA1-RA5). The SHAM group had 10 rats. The GGNX group had 12 rats. The data are presented as mean ± SEM. **p* < 0.05, significant response. ^†^ *p* < 0.05, GGNX rats versus SHAM rats.

SUPPLEMENTARY FIGURE S14
**(A)** Arithmetic changes in End Inspiratory Pause (End Insp Pause, EIP) from Pre-values in sham-operated (SHAM) rats and in rats with bilateral ganglioglomerular nerve transection (GGNX) during the first 90 s upon return to room-air (RA1-RA5), following the five hypoxic (HX, 10% O_2_, 90% N_2_) gas challenges. **(B)** Total arithmetic changes in EIP (ΔEIP) during the first 90 s of the return to room-air phases (RA1-RA5). **(C)** Total arithmetic changes in EIP (ΔEIP) during the first 5 min of the return to room-air phases (RA1-RA5). The SHAM group had 10 rats. The GGNX group had 12 rats. The data are presented as mean ± SEM. **p* < 0.05, significant response. ^†^ *p* < 0.05, GGNX rats versus SHAM rats.

SUPPLEMENTARY FIGURE S15
**(A)** Arithmetic changes in End Expiratory Pause (End Exp Pause, EEP) from Pre-values in sham-operated (SHAM) rats and in rats with bilateral ganglioglomerular nerve transection (GGNX) during the first 90 s upon return to room-air (RA1-RA5), following the five hypoxic (HX, 10% O_2_, 90% N_2_) gas challenges. **(B)** Total arithmetic changes in EEP (ΔEEP) during the first 90 s of the return to room-air phases (RA1-RA5). **(C)** Total arithmetic changes in EEP(ΔEEP) during the first 5 min of the return to room-air phases (RA1-RA5). The SHAM group had 10 rats. The GGNX group had 12 rats. The data are presented as mean ± SEM. **p* < 0.05, significant response. ^†^ *p* < 0.05, GGNX rats versus SHAM rats.

SUPPLEMENTARY FIGURE S16
**(A)** Arithmetic changes in Peak Inspiratory Flow (Peak Insp Flow, PIF) from Pre-values in sham-operated (SHAM) rats and in rats with bilateral ganglioglomerular nerve transection (GGNX) during the first 90 s upon return to room-air (RA1-RA5), following the five hypoxic (HX, 10% O_2_, 90% N_2_) gas challenges. **(B)** Total arithmetic changes in PIF (ΔPIF) during the first 90 s of the return to room-air phases (RA1-RA5). **(C)** Total arithmetic changes in PIF (ΔPIF) during the first 5 min of the return to room-air phases (RA1-RA5). The SHAM group had 10 rats. The GGNX group had 12 rats. The data are presented as mean ± SEM. **p* < 0.05, significant response. ^†^ *p* < 0.05, GGNX rats versus SHAM rats.

SUPPLEMENTARY FIGURE S17
**(A)** Arithmetic changes in Peak Expiratory Flow (Peak Exp Flow, PEF) from Pre-values in sham-operated (SHAM) rats and in rats with bilateral ganglioglomerular nerve transection (GGNX) during the first 90 s upon return to room-air (RA1-RA5), following the five hypoxic (HX, 10% O_2_, 90% N_2_) gas challenges. **(B)** Total arithmetic changes in PEF (ΔPEF) during the first 90 s of the return to room-air phases (RA1-RA5). **(C)** Total arithmetic changes in PEF (ΔPEF) during the first 5 min of the return to room-air phases (RA1-RA5). The SHAM group had 10 rats. The GGNX group had 12 rats. The data are presented as mean ± SEM. **p* < 0.05, significant response. ^†^ *p* < 0.05, GGNX rats versus SHAM rats.

SUPPLEMENTARY FIGURE S18
**(A)** Arithmetic changes in Peak Expiratory Flow/Peak Inspiratory Flow (PEF/PIF) from Pre-values in sham-operated (SHAM) rats and in rats with bilateral ganglioglomerular nerve transection (GGNX) during the first 90 s upon return to room-air (RA1-RA5), following the five hypoxic (HX, 10% O_2_, 90% N_2_) gas challenges. **(B)** Total arithmetic changes in PEF/PIF (ΔPEF/(ΔPIF) during the first 90 s of the return to room-air phases (RA1-RA5). **(C)** Total arithmetic changes in PEF/PIF (ΔPEF/(ΔPIF) during the first 5 min of the return to room-air phases (RA1-RA5). The SHAM group had 10 rats. The GGNX group had 12 rats. The data are presented as mean ± SEM. **p* < 0.05, significant response. ^†^ *p* < 0.05, GGNX rats versus SHAM rats.

SUPPLEMENTARY FIGURE S19
**(A)** Arithmetic changes in Expiratory Flow at 50% expired tidal volume (EF_50_) from Pre-values in sham-operated (SHAM) rats and in rats with bilateral ganglioglomerular nerve transection (GGNX) during the first 90 s upon return to room-air (RA1-RA5), following the five hypoxic (HX, 10% O_2_, 90% N_2_) gas challenges. **(B)** Total arithmetic changes in EF_50_ (ΔEF_50_) during the first 90 s of the return to room-air phases (RA1-RA5). **(C)** Total arithmetic changes in EF_50_ (ΔEF_50_) during the first 5 min of the return to room-air phases (RA1-RA5). The SHAM group had 10 rats. The GGNX group had 12 rats. The data are presented as mean ± SEM. **p* < 0.05, significant response. ^†^ *p* < 0.05, GGNX rats versus SHAM rats.

SUPPLEMENTARY FIGURE S20
**(A)** Arithmetic changes in Relaxation Time (RT) from Pre-values in sham-operated (SHAM) rats and in rats with bilateral ganglioglomerular nerve transection (GGNX) during the first 90 s upon return to room-air (RA1-RA5), following the five hypoxic (HX, 10% O_2_, 90% N_2_) gas challenges. **(B)** Total arithmetic changes in RT (ΔRT) during the first 90 s of the return to room-air phases (RA1-RA5). **(C)** Total arithmetic changes in RT (ΔRT) during the first 5 min of the return to room-air phases (RA1-RA5). The SHAM group had 10 rats. The GGNX group had 12 rats. The data are presented as mean ± SEM. **p* < 0.05, significant response. ^†^ *p* < 0.05, GGNX rats versus SHAM rats.

SUPPLEMENTARY FIGURE S21
**(A)** Arithmetic changes in Apneic Pause from Pre-values in sham-operated (SHAM) rats and in rats with bilateral ganglioglomerular nerve transection (GGNX) during the first 90 s upon return to room-air (RA1-RA5), following the five hypoxic (HX, 10% O_2_, 90% N_2_) gas challenges. **(B)** Total arithmetic changes in Apneic Pause during the first 90 s of the return to room-air phases (RA1-RA5). **(C)** Total arithmetic changes in Apneic Pause during the first 5 min of the return to room-air phases (RA1-RA5). The SHAM group had 10 rats. The GGNX group had 12 rats. The data are presented as mean ± SEM. **p* < 0.05, significant response.

SUPPLEMENTARY FIGURE S22
**(A)** Arithmetic changes in Inspiratory Drive (Insp Drive) from Pre-values in sham-operated (SHAM) rats and in rats with bilateral ganglioglomerular nerve transection (GGNX) during the first 90 s upon return to room-air (RA1-RA5), following the five hypoxic (HX, 10% O_2_, 90% N_2_) gas challenges. **(B)** Total arithmetic changes in Inspiratory Drive (ΔInsp Drive) during the first 90 s of the return to room-air phases (RA1-RA5). **(C)** Total arithmetic changes in Inspiratory Drive (ΔInsp Drive) during the first 5 min of the return to room-air phases (RA1-RA5). The SHAM group had 10 rats. The GGNX group had 12 rats. The data are presented as mean ± SEM. **p* < 0.05, significant response. ^†^ *p* < 0.05, GGNX rats versus SHAM rats.

SUPPLEMENTARY FIGURE S23
**(A)** Arithmetic changes in Expiratory Drive (Exp Drive) from Pre-values in sham-operated (SHAM) rats and in rats with bilateral ganglioglomerular nerve transection (GGNX) during the first 90 s upon return to room-air (RA1-RA5), following the five hypoxic (HX, 10% O_2_, 90% N_2_) gas challenges. **(B)** Total arithmetic changes in Exp Drive (ΔExp Drive) during the first 90 s of the return to room-air phases (RA1-RA5). **(C)** Total arithmetic changes in Exp Drive (ΔExp Drive) during the first 5 min of the return to room-air phases (RA1-RA5). The SHAM group had 10 rats. The GGNX group had 12 rats. The data are presented as mean ± SEM. **p* < 0.05, significant response. ^†^ *p* < 0.05, GGNX rats versus SHAM rats.

SUPPLEMENTARY FIGURE S24
**(A)** Arithmetic changes in Non-Eupneic Breathing Index/Frequency of breathing index (NEBI/Freq) from Pre-values in sham-operated (SHAM) rats and in rats with bilateral ganglioglomerular nerve transection (GGNX) during the first 90 s upon return to room-air (RA1-RA5), following the five hypoxic (HX, 10% O_2_, 90% N_2_) gas challenges. **(B)** Total arithmetic changes in NEBI/Freq during the first 90 s of the return to room-air phases (RA1-RA5). **(C)** Total arithmetic changes in NEBI/Freq during the first 5 min of the return to room-air phases (RA1-RA5). The SHAM group had 10 rats. The GGNX group had 12 rats. The data are presented as mean ± SEM. **p* < 0.05, significant response. ^†^ *p* < 0.05, GGNX rats versus SHAM rats.

SUPPLEMENTARY TABLE S1 Definition of ventilatory parameters described in this study.

SUPPLEMENTARY TABLE S2 Information and resting (Pre HX challenge) ventilatory parameters.

SUPPLEMENTARY TABLE S3 Qualitative status of resting (Pre-challenge) ventilatory parameters in GGNX and CSNX rats compared to their respective sham-operated (SHAM) controls.

SUPPLEMENTARY TABLE S4 Qualitative status of ventilatory responses during HX1-HX5 in GGNX rats compared to SHAM rats.

SUPPLEMENTARY TABLE S5 Qualitative status of ventilatory responses during RA1-RA5 in GGNX rats compared to SHAM rats.

## Abbreviations

CSC, cervical sympathetic chain; CSCX, cervical sympathetic chain transection; CSN, carotid sinus nerve; CSNX, carotid sinus nerve transection; ECN, external carotid nerve; EF_50_, expiratory flow at 50% expired tidal volume; GGN, ganglioglomerular nerve; GGNX, ganglioglomerular nerve transection; HXC, hypoxic gas challenge; ICN, internal carotid nerve; SCG, superior cervical ganglion; SCGX, superior cervical ganglionectomy.
