# Supplementary material for: Loss of ganglioglomerular nerve input to the carotid body impacts the hypoxic ventilatory response in freely-moving rats
Source: Front Physiol. 2023 Mar 16;14:1007043. doi: 10.3389/fphys.2023.1007043 (PMC10060956; doi:10.3389/fphys.2023.1007043)
Supplement: Supplementary file 1 [file DataSheet1.docx]

**Supplementary File**

**Loss of ganglioglomerular nerve input to the carotid body impacts**

**the hypoxic ventilatory response in freely-moving rats**

Paulina M. Getsy^1,^*

Gregory A. Coffee^1^

Stephen J. Lewis^1,2,3^

*^1^Department of Pediatrics, Division of Pulmonology, Allergy and Immunology, Case Western Reserve University, Cleveland, Ohio, USA*

*^2^Department of Pharmacology, Case Western Reserve University, Cleveland, Ohio, USA*

*^3^Functional Electrical Stimulation Center, Case Western Reserve University, Cleveland, Ohio, USA*

***Address for Correspondence**

Paulina M. Getsy, PhD

Department of Pediatrics

School of Medicine

Biomedical Research Building, Room 839A

Case Western Reserve University

10900 Euclid Avenue

Cleveland, OH 44106-4984

Phone: 216-368-5115

Email: pxg55@case.edu

**Supplementary Table S1**

Definition of ventilatory parameters described in this study

| **Parameter** | **Abbreviation** | | **Units** | | **Definition** |
| --- | --- | --- | --- | --- | --- |
| **A. Directly recorded parameters** | | | | | |
| Frequency of breaths | | Freq | | breaths/min | Rate of breathing |
| Inspiratory Time | | Ti | | sec | Duration of inspiration |
| Expiratory Time | | Te | | sec | Duration of expiration |
| End Inspiratory Pause | | EIP | | msec | Pause between end of inspiration start of expiration |
| End Expiratory Pause | | EEP | | msec | Pause between end of expiration and start of inspiration |
| Relaxation time | | RT | | sec | Decay of expiration to 36% maximum |
| Tidal Volume | | TV | | ml | Volume of inspired air per breath |
| Minute Ventilation | | MV = freq x TV | | ml/min | Total volume of air inspired per min |
| Peak Inspiratory Flow | | PIF | | ml/sec | Maximum inspiratory flow |
| Peak Expiratory Flow | | PEF | | ml/sec | Maximum expiratory flow |
| Expiratory flow at 50% | | EF_50_ | | ml/sec | Expiratory flow at 50% expired TV |
| Non-eupneic breathing index | | NEBI | | % | % of non-eupneic breaths per epoch |
| **B. Derived parameters** | | | | | |
| Te/Ti | | Te/Ti | | none | respiratory quotient |
| PIF/PEF | |  | |  |  |
| Inspiratory Drive | | TV/Ti | | ml/sec | Central urge to inhale |
| Expiratory Drive | | TV/Te | | ml/sec | Central drive to exhale |
| NEBI/Frequency | | NEBI/Freq | | %/(b/min) | Balanced rejection index |
| Apneic Pause | | AP = (Te/RT)-1 | | No units | Elongated expiration |

**Supplementary Figure S1**


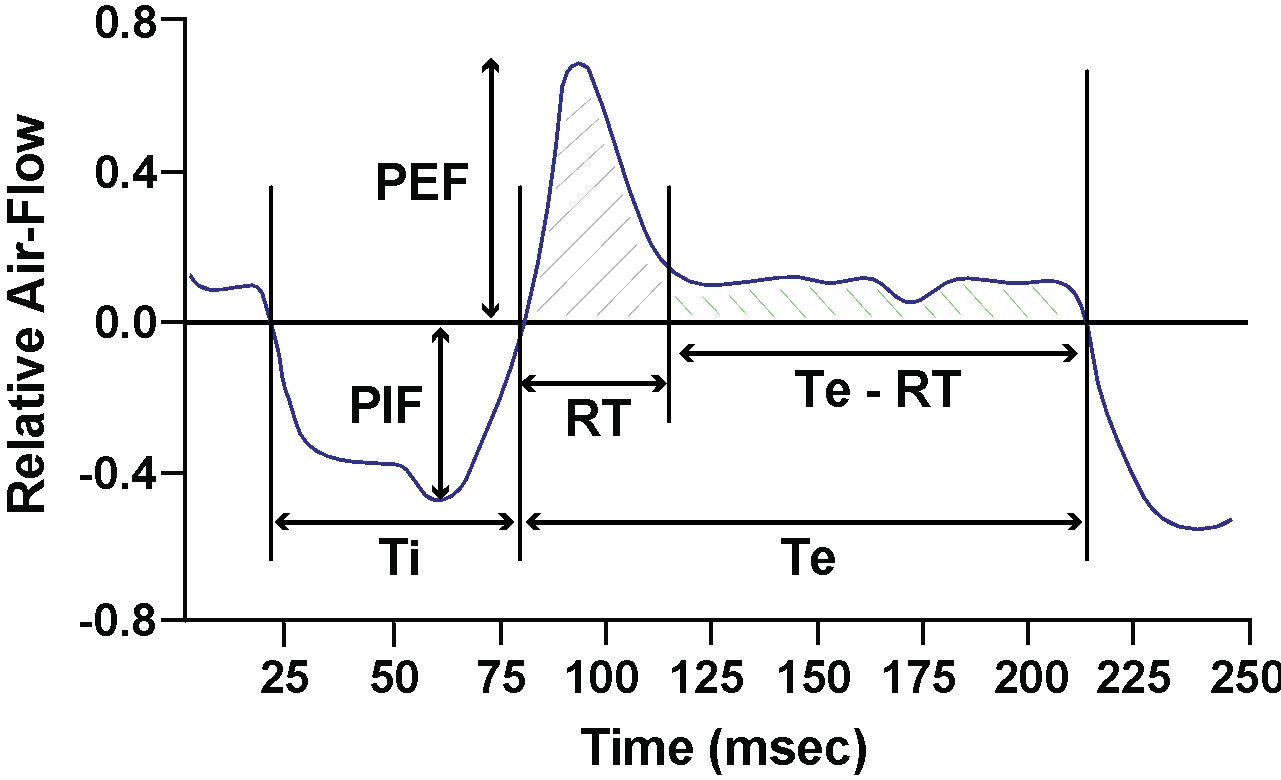


**Supplementary Figure S1.** Relationships between peak inspiratory flow (PIF), peak expiratory flow (PEF), relaxation time (RT) and expiratory time (Te).

**Supplementary Table S2**

Information and resting (Pre HX challenge) ventilatory parameters

| **Parameter** | **SHAM rats** | **GGNX rats** |
| --- | --- | --- |
| Number | 10 | 12 |
| Age, days | 25 ± 0 | 25 ± 0 |
| Body weight, grams | 50.6 ± 2.8 | 57.9 ± 4.3 |
| Frequency of breathing, breaths/min | 122 ± 2 | 111 ± 2* |
| Tidal Volume (TV), ml | 0.573 ± 0.016 | 0.578 ± 0.024 |
| Minute Ventilation, ml/min | 70.0 ± 2.4 | 63.7 ± 2.5 |
| Inspiratory time (Ti), sec | 0.194 ± 0.005 | 0.211 ± 0.005 |
| Expiratory time (Te), sec | 0.311 ± 0.005 | 0.354 ± 0.013* |
| Expiratory time/Inspiratory time | 1.63 ± 0.04 | 1.69 ± 0.06 |
| End inspiratory pause, msec | 4.17 ± 0.07 | 4.59 ± 0.17 |
| End expiratory pause, msec | 5.28 ± 0.58 | 8.79 ± 0.90* |
| Peak inspiratory flow (PIF), ml/sec | 4.6 ± 0.2 | 4.3 ± 0.1 |
| Peak expiratory flow (PEF), ml/sec | 2.9 ± 0.1 | 3.2 ± 0.2 |
| PEF/PIF | 0.64 ± 0.02 | 0.74 ± 0.02* |
| EF_50_, ml/sec | 0.149 ± 0.006 | 0.145 ± 0.006 |
| Relaxation time (RT), sec | 0.174 ± 0.005 | 0.191 ± 0.006 |
| Apneic pause, (Te/RT)-1 | 0.82 ± 0.04 | 0.88 ± 0.03 |
| Inspiratory Drive (TV/Ti, ml/sec | 3.00 ± 0.11 | 2.77 ± 0.11 |
| Expiratory Drive (TV/Te), ml/sec | 1.87 ± 0.06 | 1.66 ± 0.07 |
| NEBI, % | 19.3 ± 1.8 | 12.6 ± 1.6* |
| NEBI/frequency, %/(breaths/min) | 0.16 ± 0.01 | 0.12 ± 0.02 |

SHAM, sham-operated mice. GGNX, bilateral ganglioglomerular nerve transection. EF_50_, expiratory flow at 50% expired tidal volume. NEBI, non-eupneic breathing index. The data are presented as mean ± SEM. There were 10 rats in the SHAM group and 12 rats in the GGNX group. **p* < 0.05, GGNX *versus* SHAM.

**Supplementary Figure S2**

**A.**


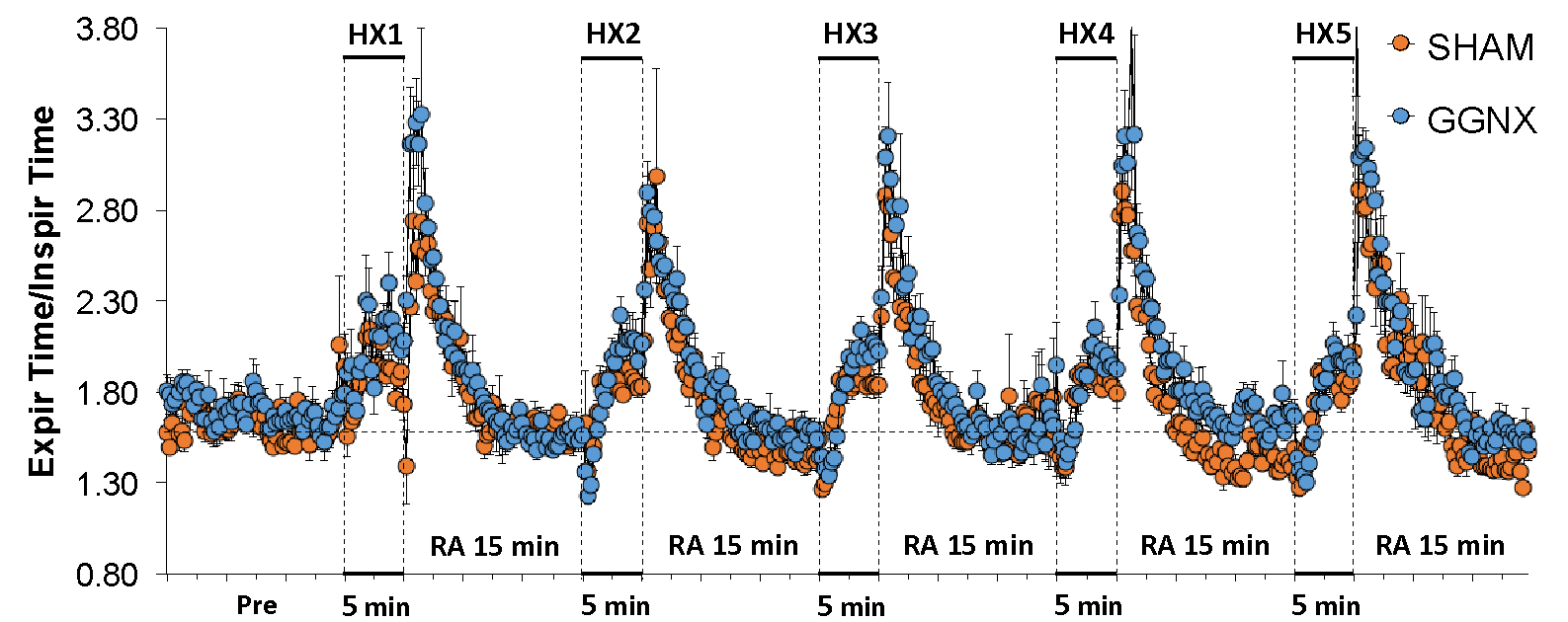


**C. 90 sec totals**

**D. 5 min totals**

**B.**


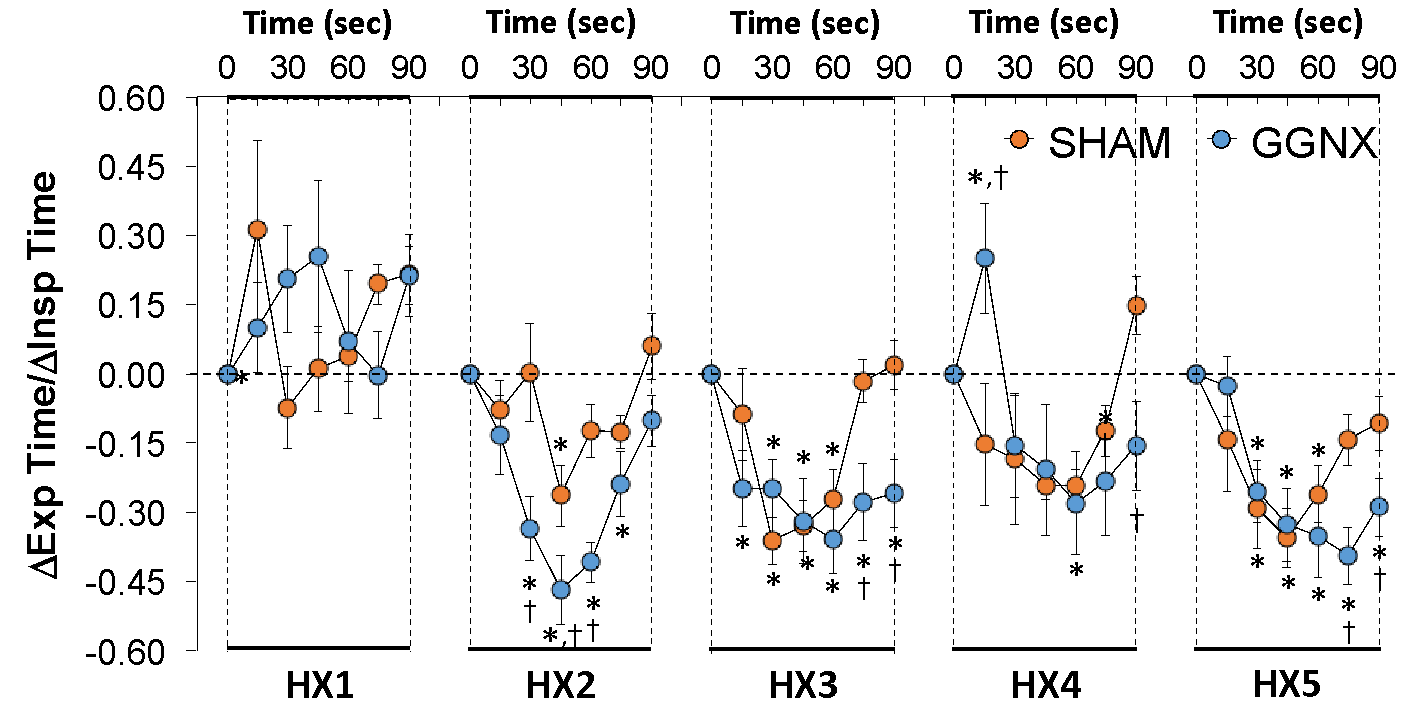


**C. 90 sec totals**

**D. 5 min totals**


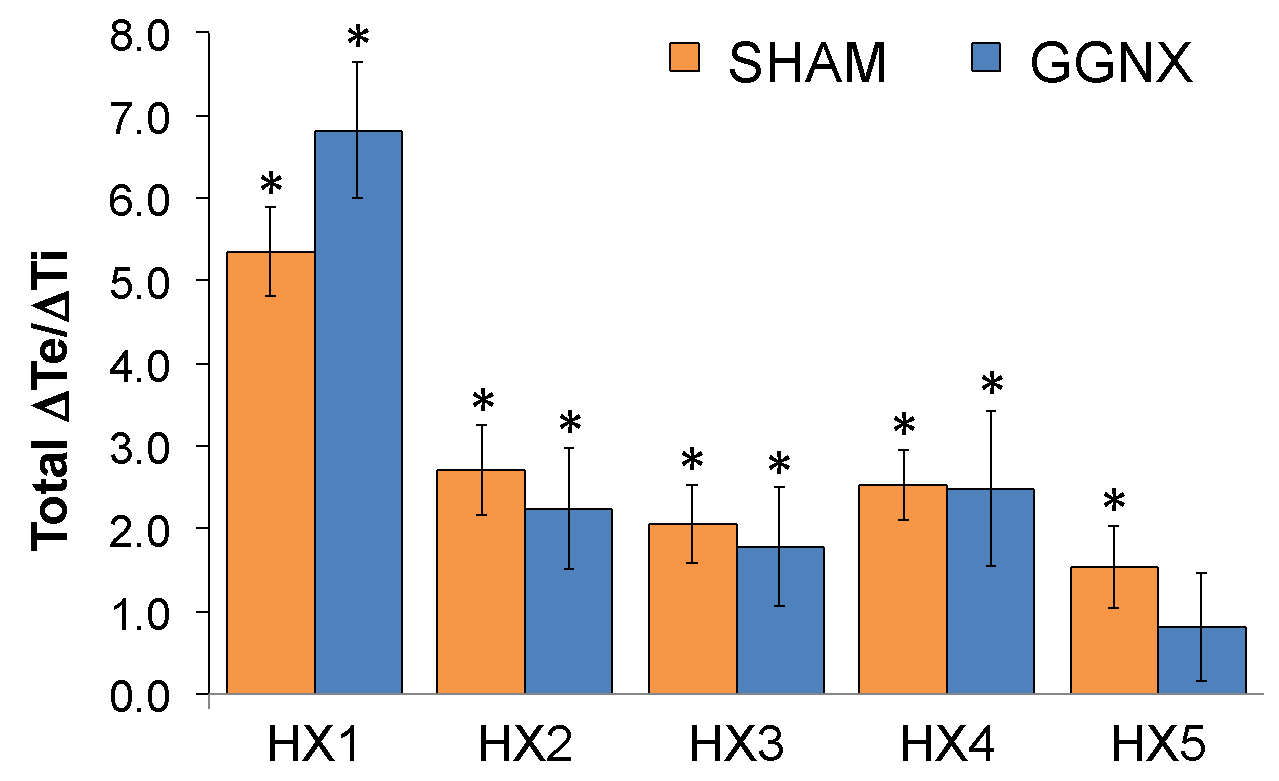

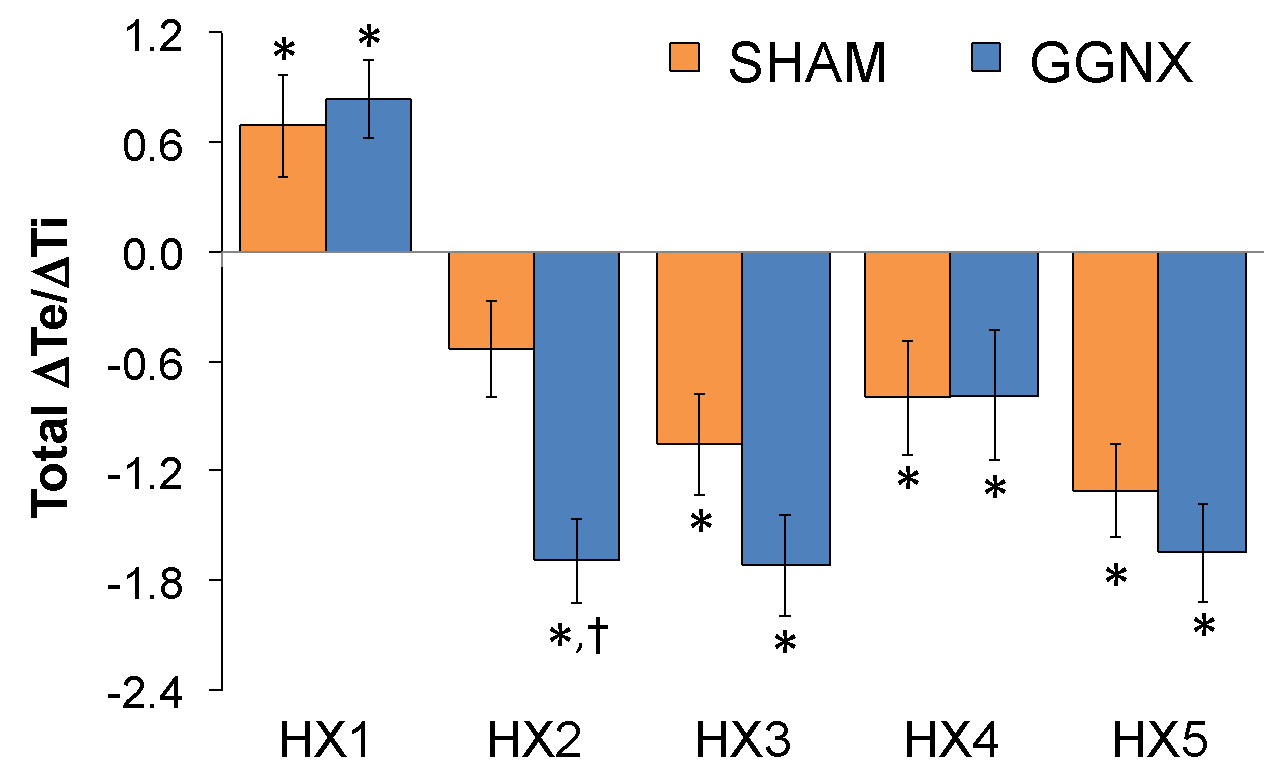


**Supplementary Figure S2. Panel A:** Expiratory time/Inspiratory time (Exp time/Insp time or Expir Time/ Inspir Time) in sham-operated (SHAM) rats and in rats with bilateral ganglioglomerular nerve transection (GGNX) before (Pre) and during five hypoxic (HX, 10% O_2_, 90% N_2_) gas challenges, each separated by 15 min of room-air (RA). **Panel B:** Arithmetic changes in expiratory time/Arithmetic changes in inspiratory time ((ΔTe (sec)/(ΔTi (sec)) during the first 90 sec of HX gas challenge. **Panel C:** Total changes in expiratory time/total changes in inspiratory time ((Total ΔTe (sec)/(Total ΔTi (sec)) during the first 90 sec of the HX gas challenge. **Panel D:** Total changes in expiratory time/total changes in inspiratory time ((Total ΔTe (sec)/(Total ΔTi (sec)) during the entire 5 min of the HX gas challenge. The SHAM group had 10 rats. The GGNX group had 12 rats. Data are presented as mean ± SEM. **p* < 0.05, significant response. ^†^*p* < 0.05, GGNX rats *versus* SHAM rats.

**Supplementary Figure S3**




**B.**


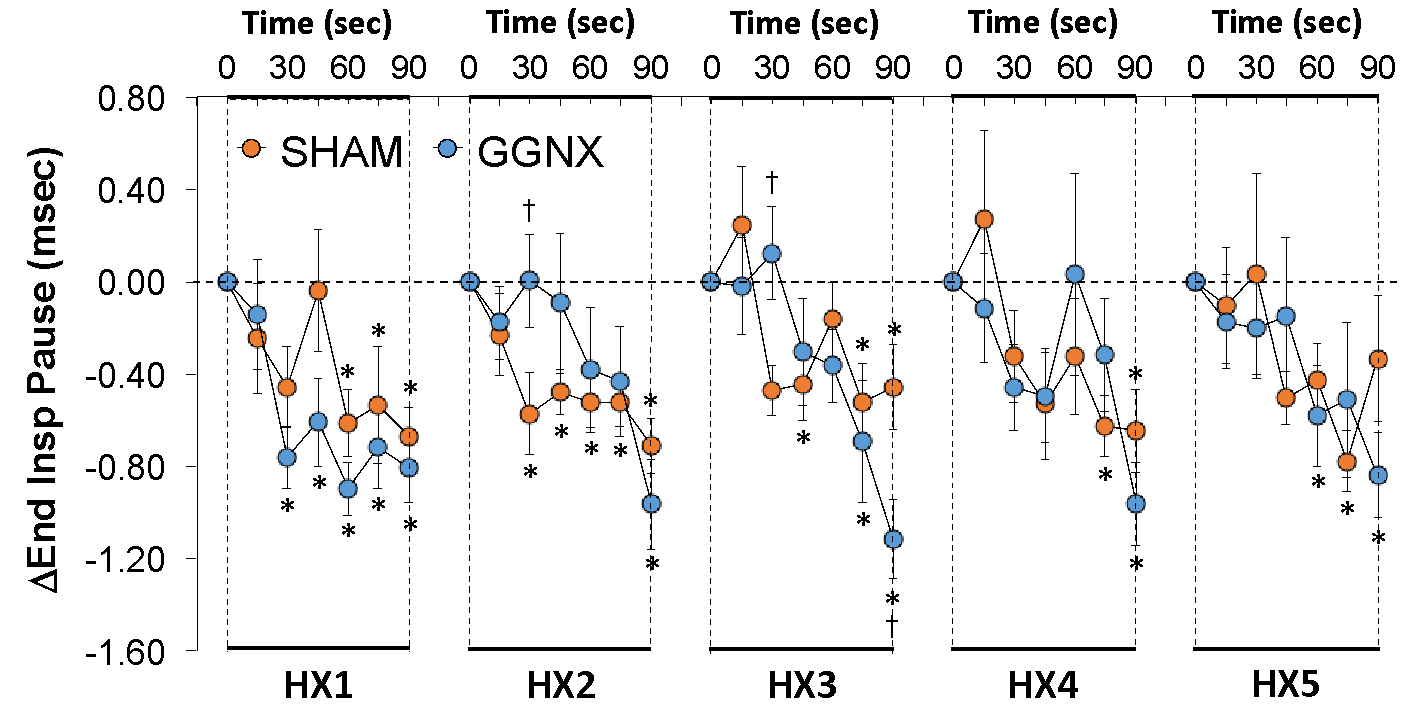


**C. 90 sec totals**

**D. 5 min totals**


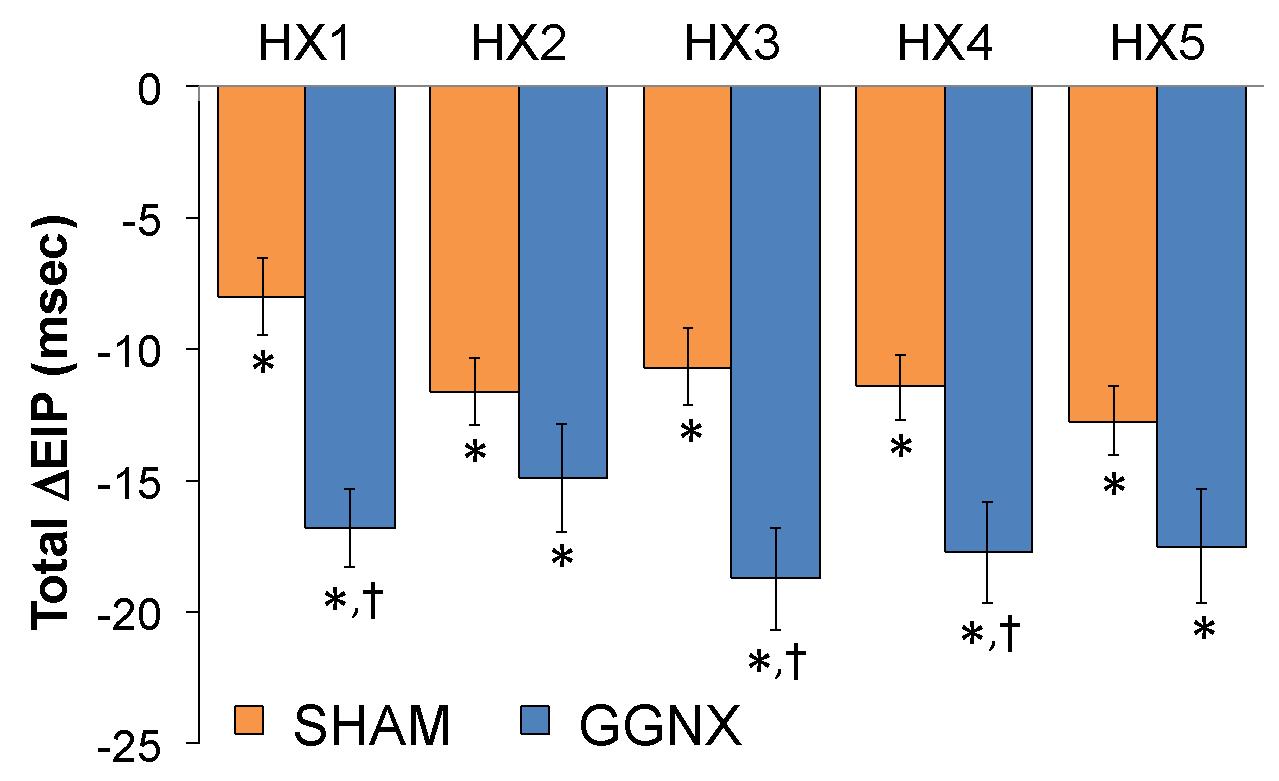

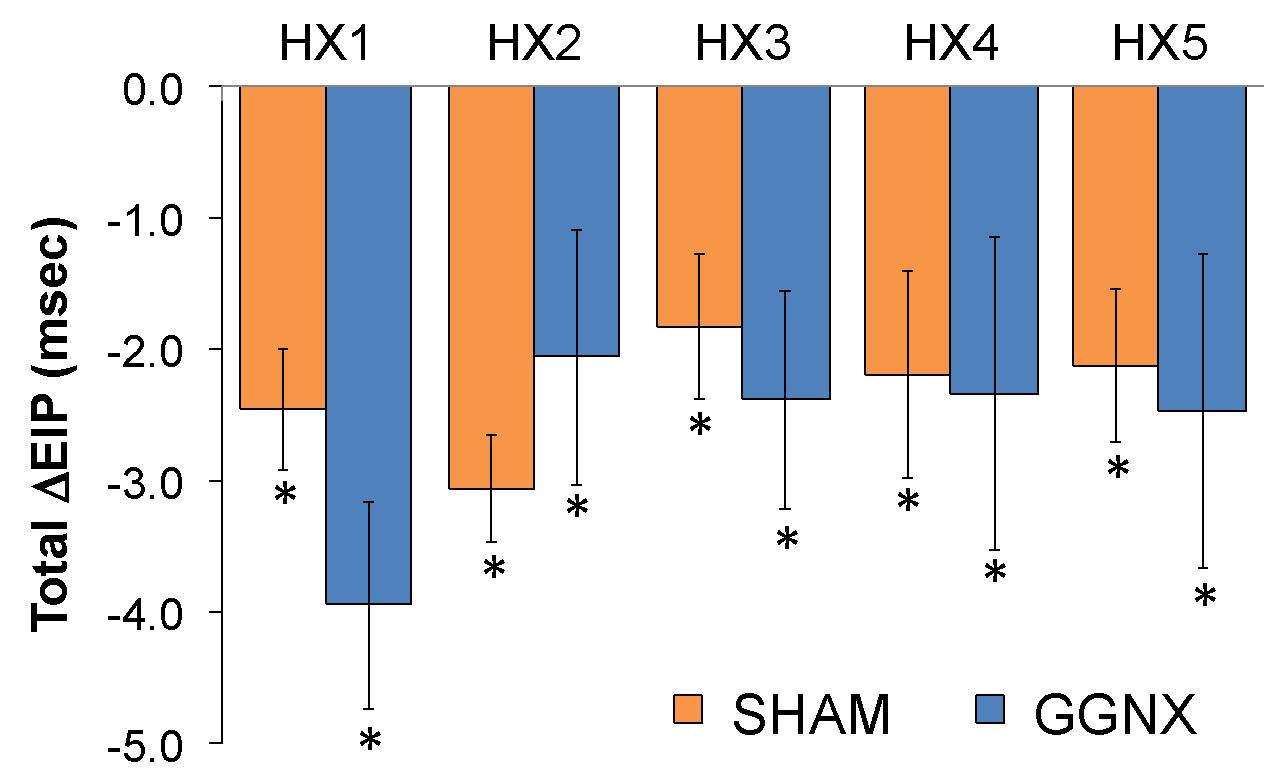


**Supplementary Figure S3. Panel A:** End Inspiratory Pause (EIP or End Insp Pause) in sham-operated (SHAM) rats and in rats with bilateral ganglioglomerular nerve transection (GGNX) before (Pre) and during five hypoxic (HX, 10% O_2_, 90% N_2_) gas challenges, each separated by 15 min of room-air (RA). **Panel B:** Arithmetic changes in EIP (ΔEnd Insp Pause) during the first 90 sec of HX gas challenge. **Panel C:** Total changes in end inspiratory pause (Total ΔEIP) during the first 90 sec of HX gas challenge. **Panel D:** Total changes in end inspiratory pause (Total ΔEIP) during the entire 5 min of HX gas challenge. The SHAM group had 10 rats. The GGNX group had 12 rats. The data are presented as mean ± SEM. **p* < 0.05, significant response. ^†^*p* < 0.05, GGNX rats *versus* SHAM rats.

**Supplementary Figure S4**

**A.**


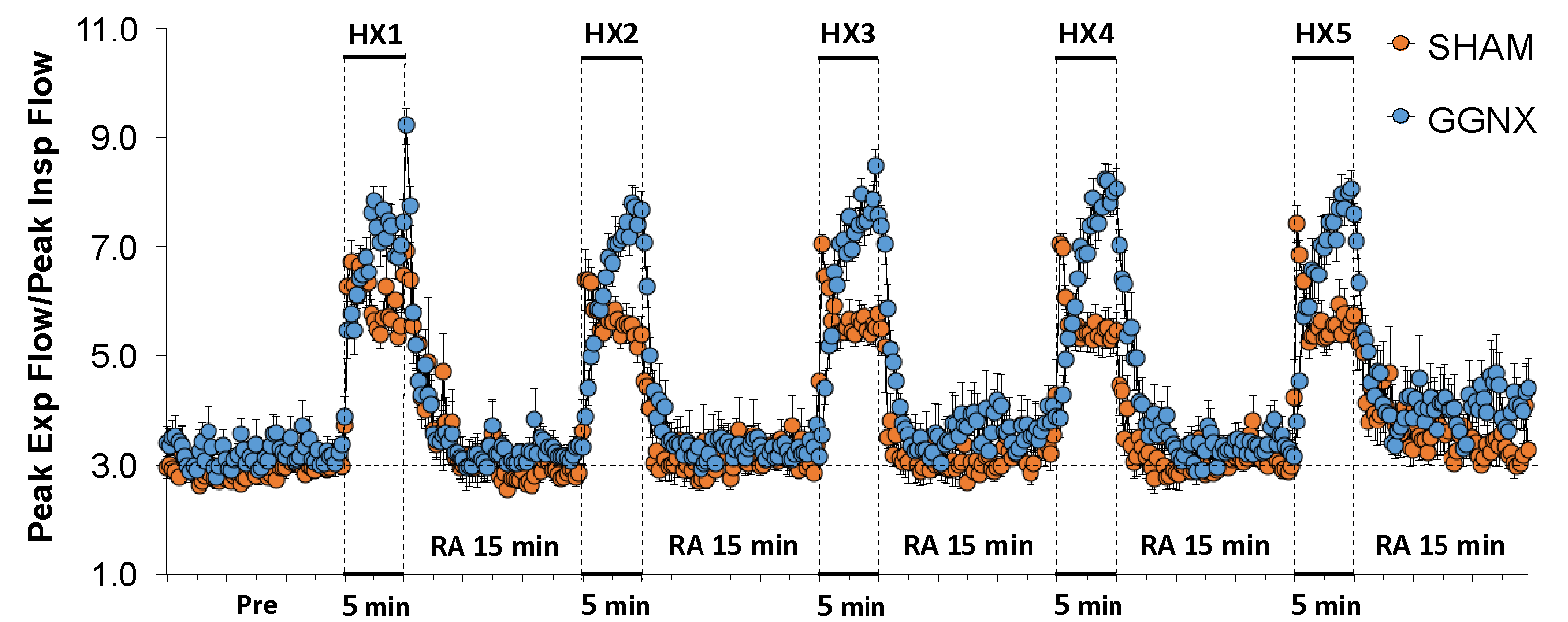


**B.**

**B.**


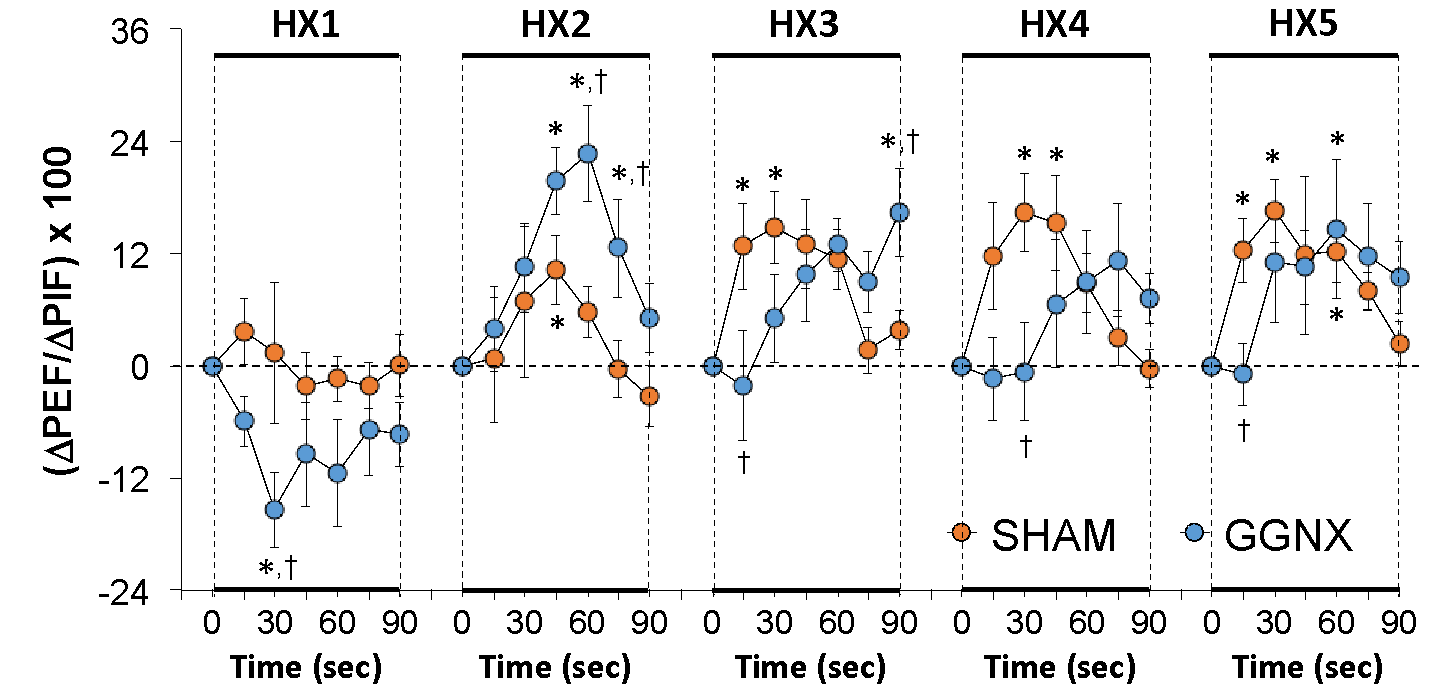


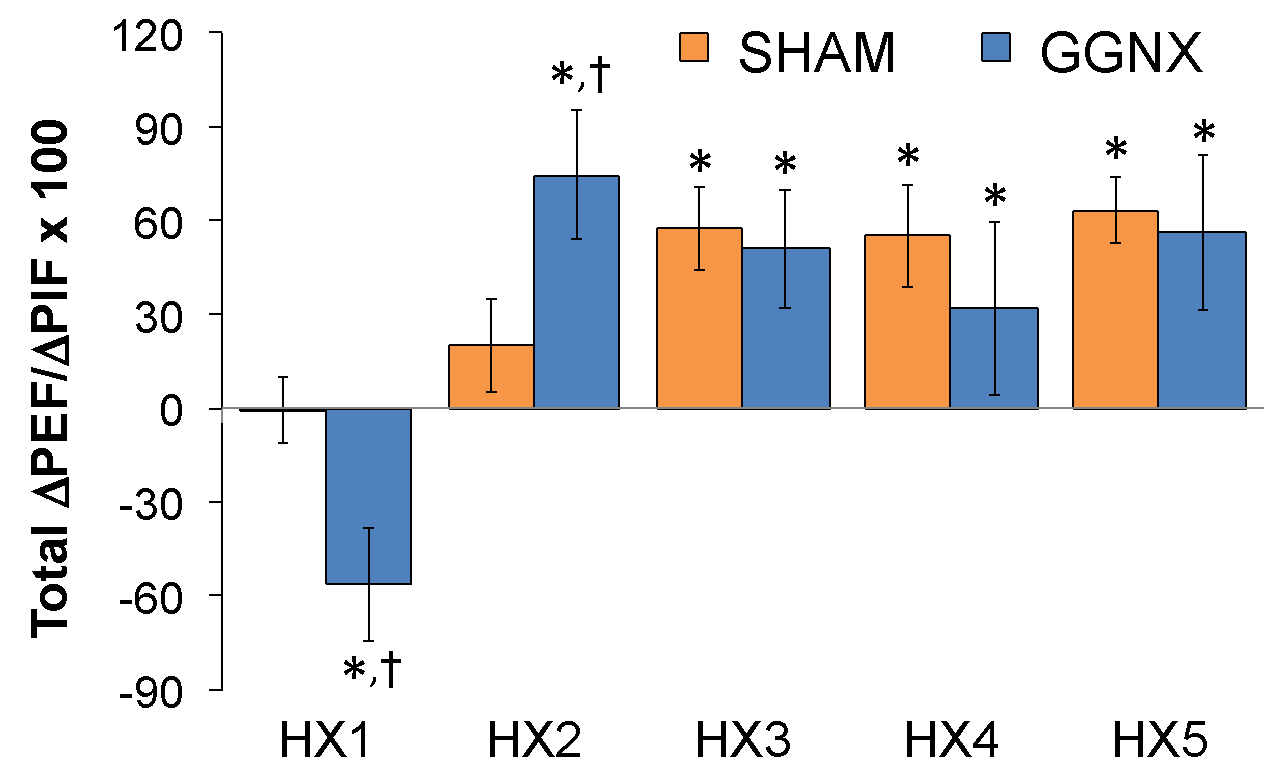

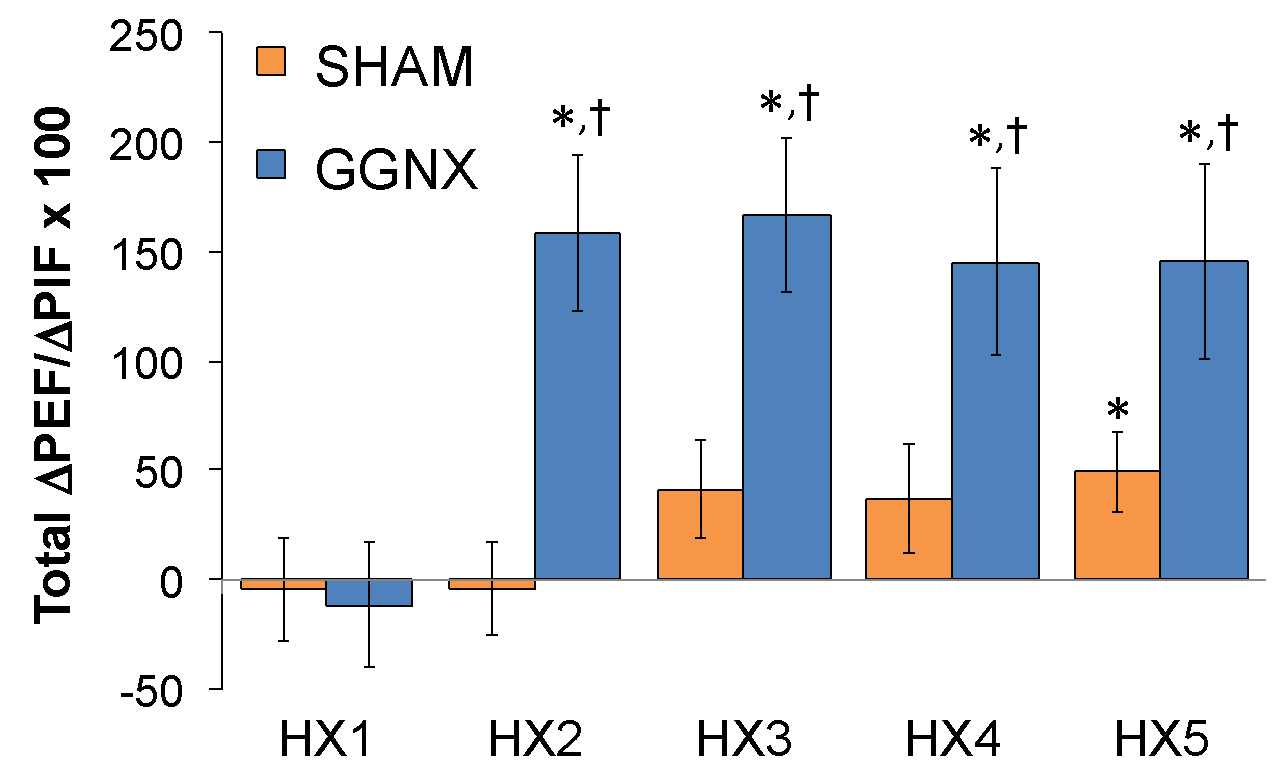


**C. 90 sec totals**

**D. 5 min totals**

**Supplementary Figure S4. Panel A:** Peak expiratory flow/peak inspiratory flow (PEF/PIF) ratios in sham-operated (SHAM) rats and in rats with bilateral ganglioglomerular nerve transection (GGNX) before (Pre) and during five hypoxic (HX, 10% O_2_, 90% N_2_) gas challenges, each separated by 15 min room-air (RA). **Panel B:** Arithmetic changes in PEF/PIF during the first 90 sec of HX gas challenge. **Panel C:** Total changes in PEF/PIF during the first 90 sec of HX gas challenge. **Panel D:** Total changes in PEF/PIF during the entire 5 min HX gas challenge. The SHAM group had 10 rats. The GGNX group had 12 rats. Data are presented as mean ± SEM. **p* < 0.05, significant response. ^†^*p* < 0.05, GGNX rats *versus* SHAM rats.

**Supplementary Figure S5**

**A.**


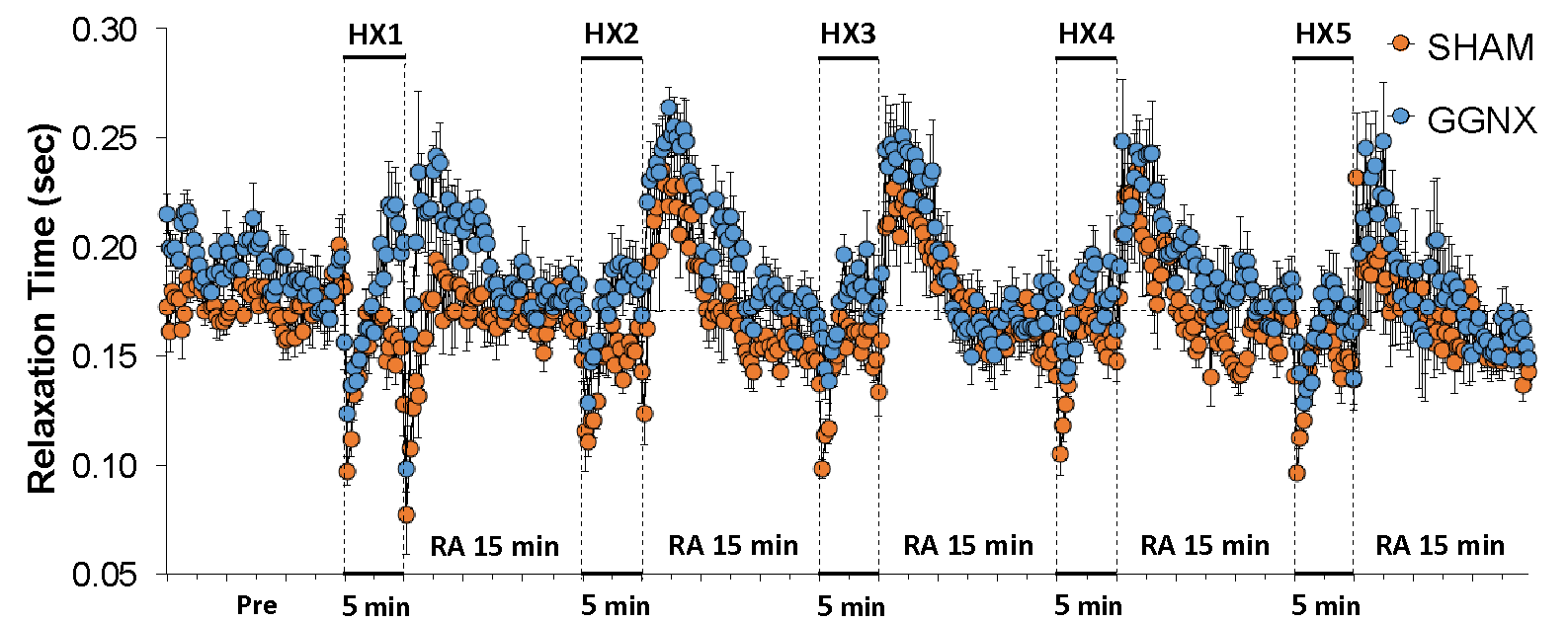


**A.**

**B.**


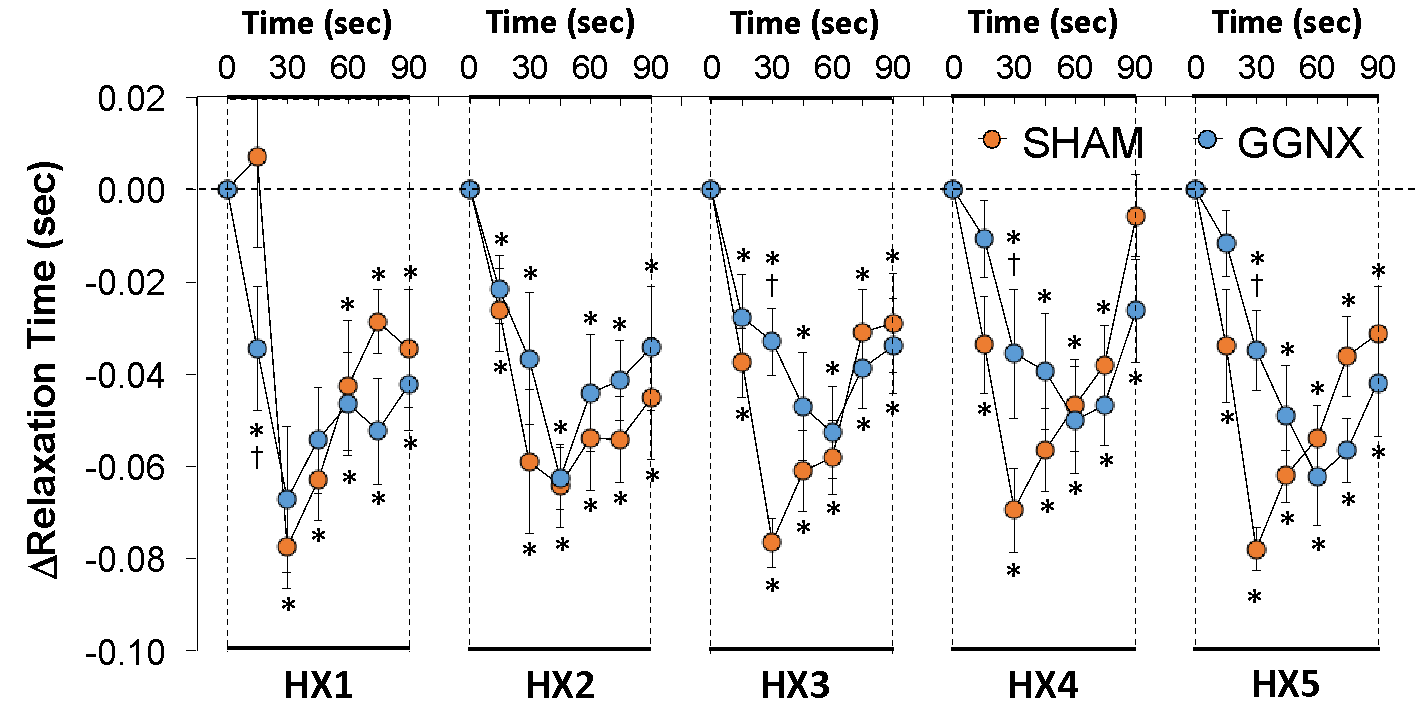


**D. 5 min totals**

**C. 90 sec totals**


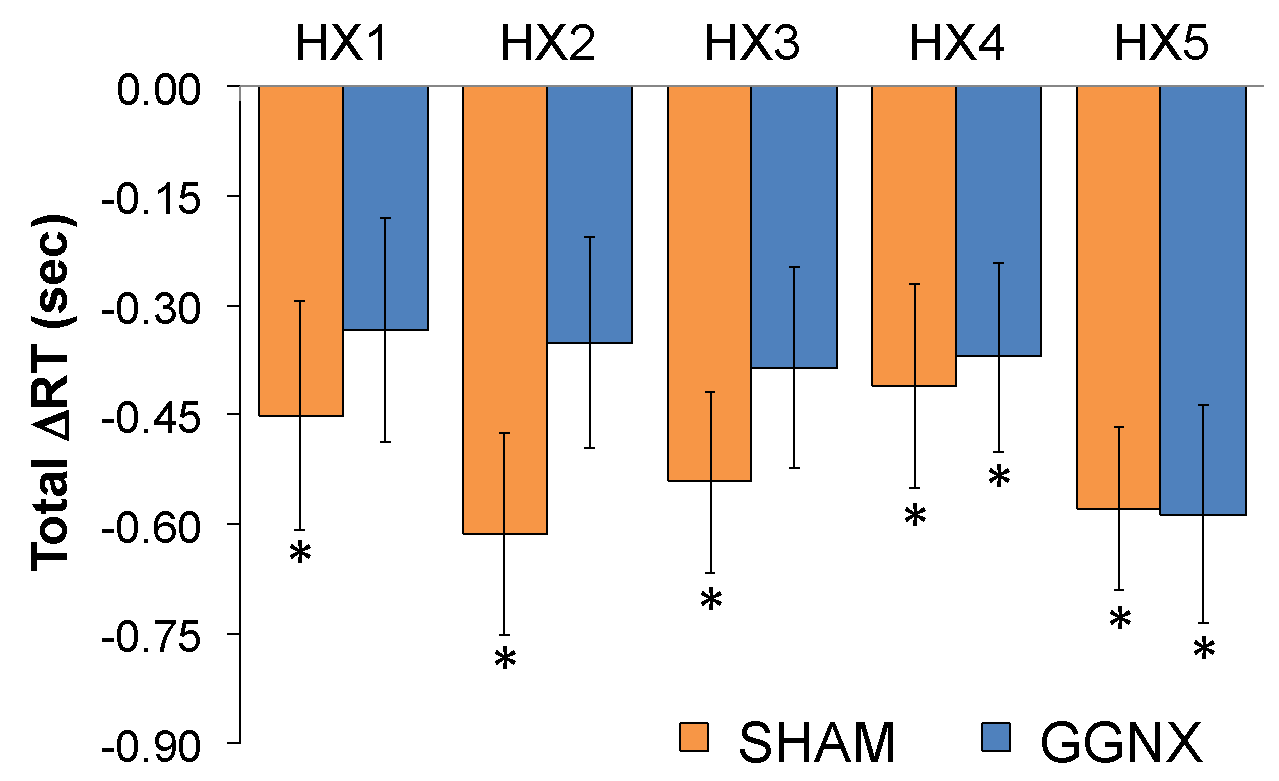

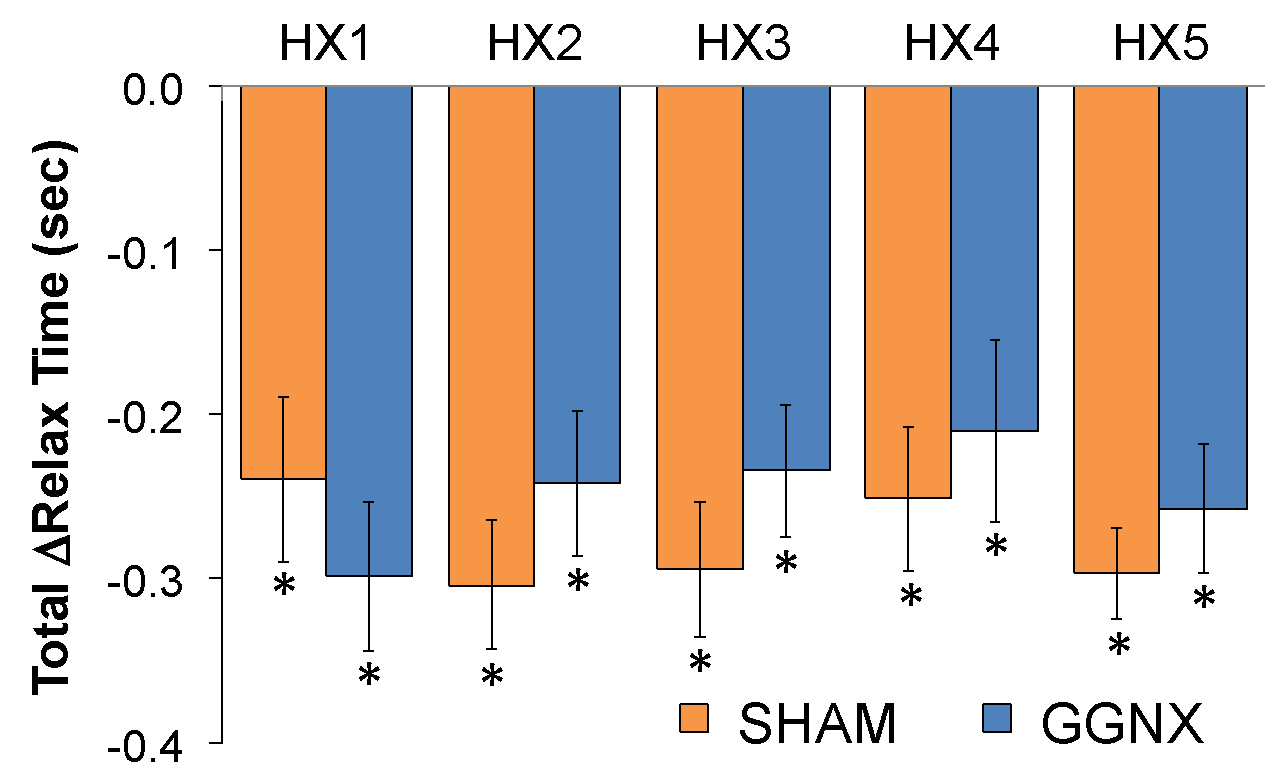


**Supplementary Figure S5. Panel A:** Relaxation time in sham-operated (SHAM) rats and in rats with bilateral ganglioglomerular nerve transection (GGNX) before (Pre) and during five hypoxic (HX, 10% O_2_, 90% N_2_) gas challenges, each separated by 15 min of room-air (RA). **Panel B:** Arithmetic changes in relaxation time during the first 90 sec of HX gas challenge. **Panel C:** Total changes in relaxation time during the first 90 sec and **Panel D** over the entire 5 min of HX gas challenge. The SHAM group had 10 rats. The GGNX group had 12 rats. The data are presented as mean ± SEM. **p* < 0.05, significant response. ^†^*p* < 0.05, GGNX rats *versus* SHAM rats.

**Supplementary Figure S6**

**A.**


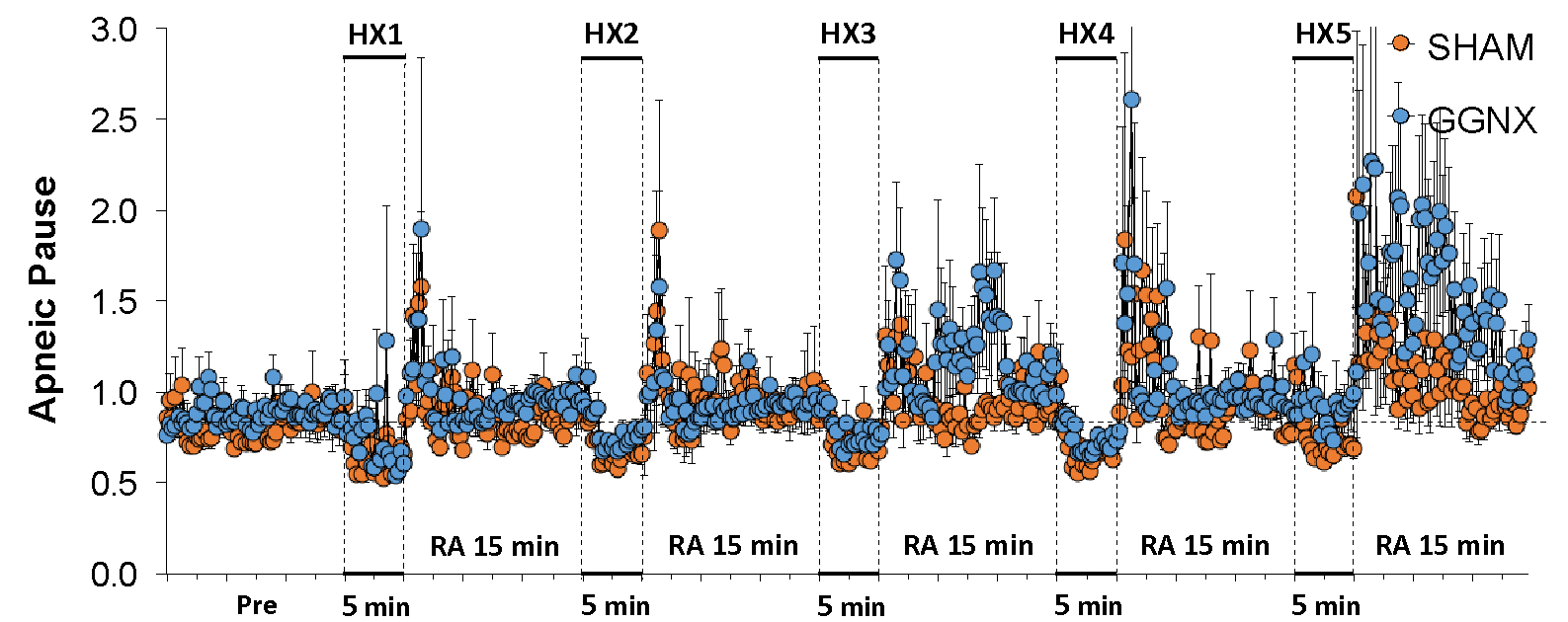


**B.**


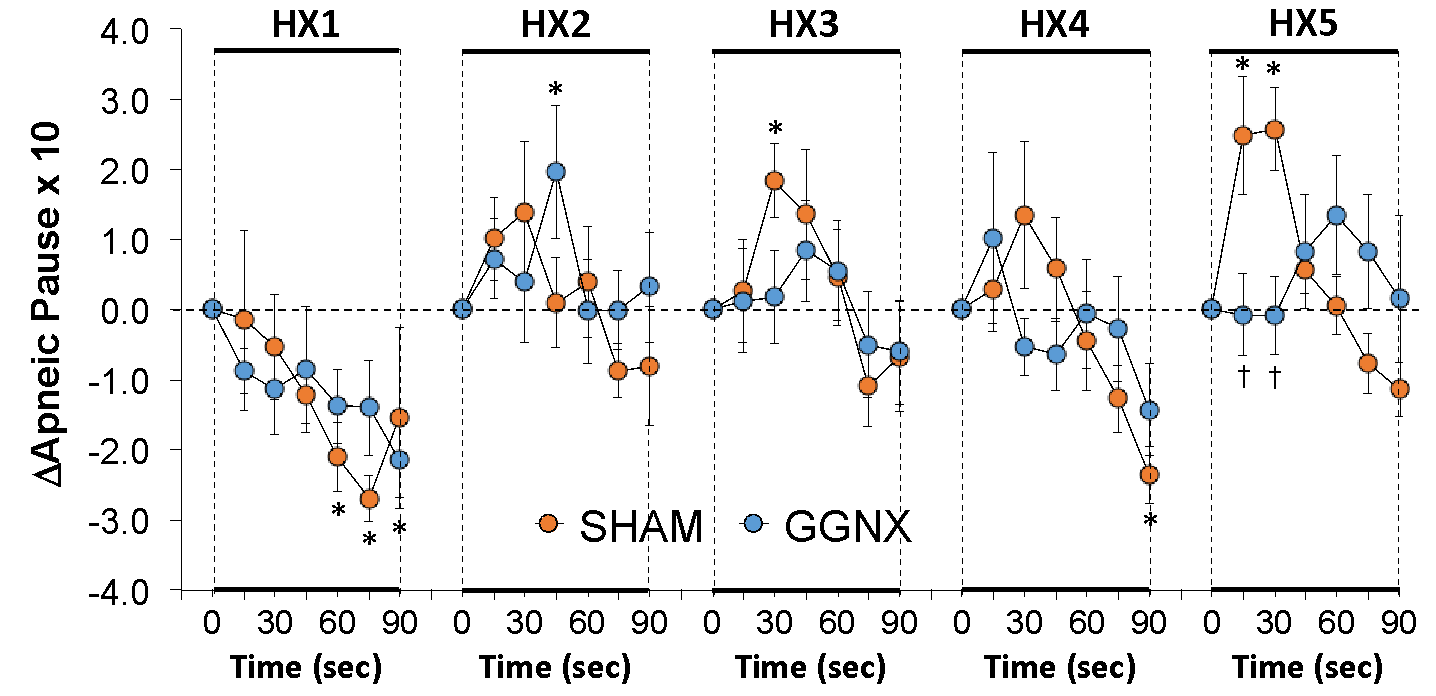


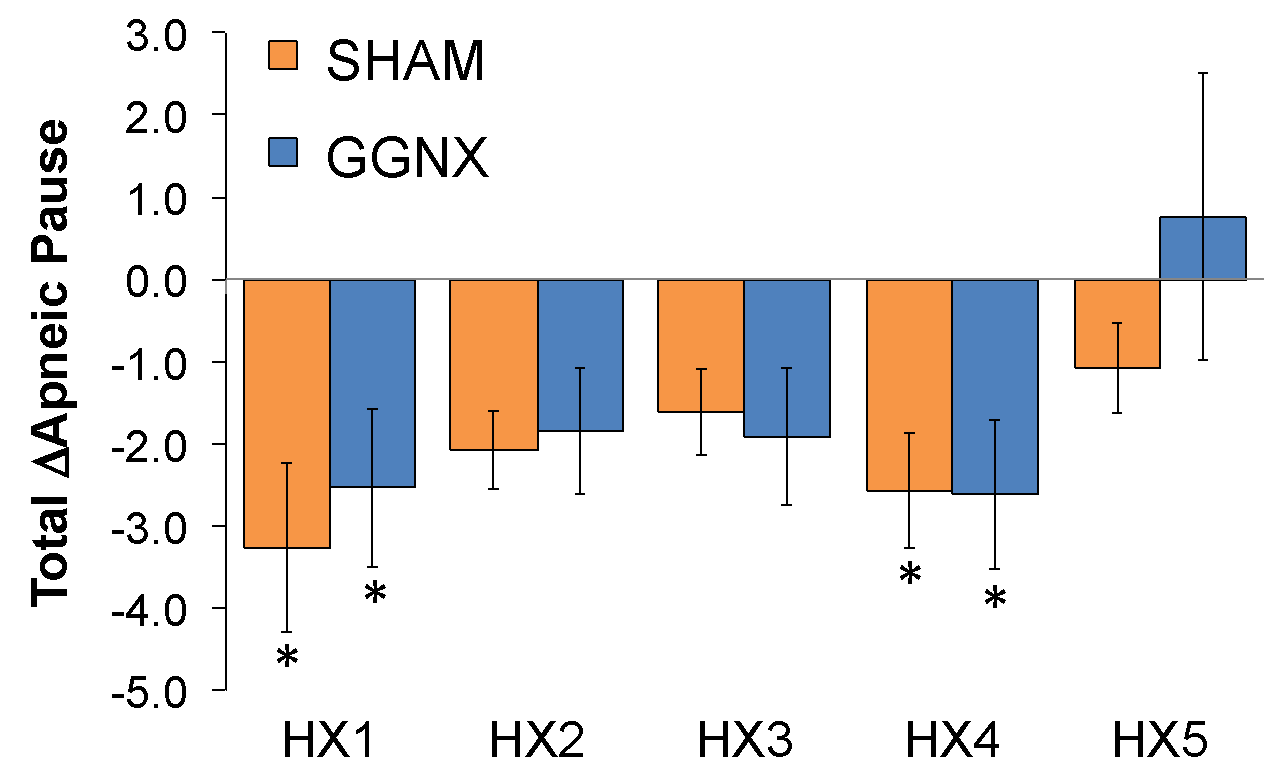

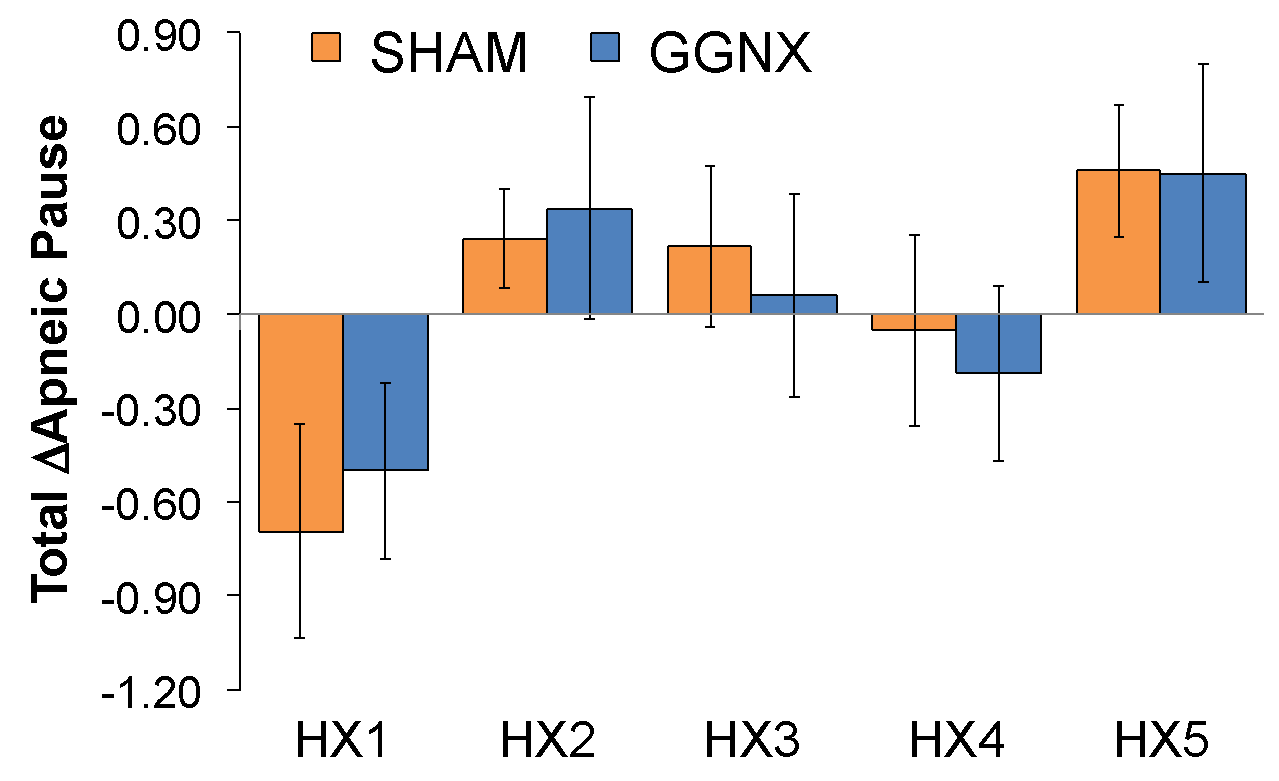


**D. 5 min totals**

**C. 90 sec totals**

**Supplementary Figure S6. Panel A:** Apneic pause values in sham-operated (SHAM) rats and in rats with bilateral ganglioglomerular nerve transection (GGNX) before (Pre) and during five hypoxic (HX, 10% O_2_, 90% N_2_) gas challenges, each separated by 15 min of room-air (RA). **Panel B:** Arithmetic changes in apneic pause during the first 90 sec of HX gas challenge. Total changes in apneic pause during the first 90 sec (**Panel C**) and over the entire 5 min (**Panel D**) of HX gas challenge. The SHAM group had 10 rats. The GGNX group had 12 rats. The data are presented as mean ± SEM. **p* < 0.05, significant response. ^†^*p* < 0.05, GGNX rats *versus* SHAM rats.

**Supplementary Figure S7**

**A.**


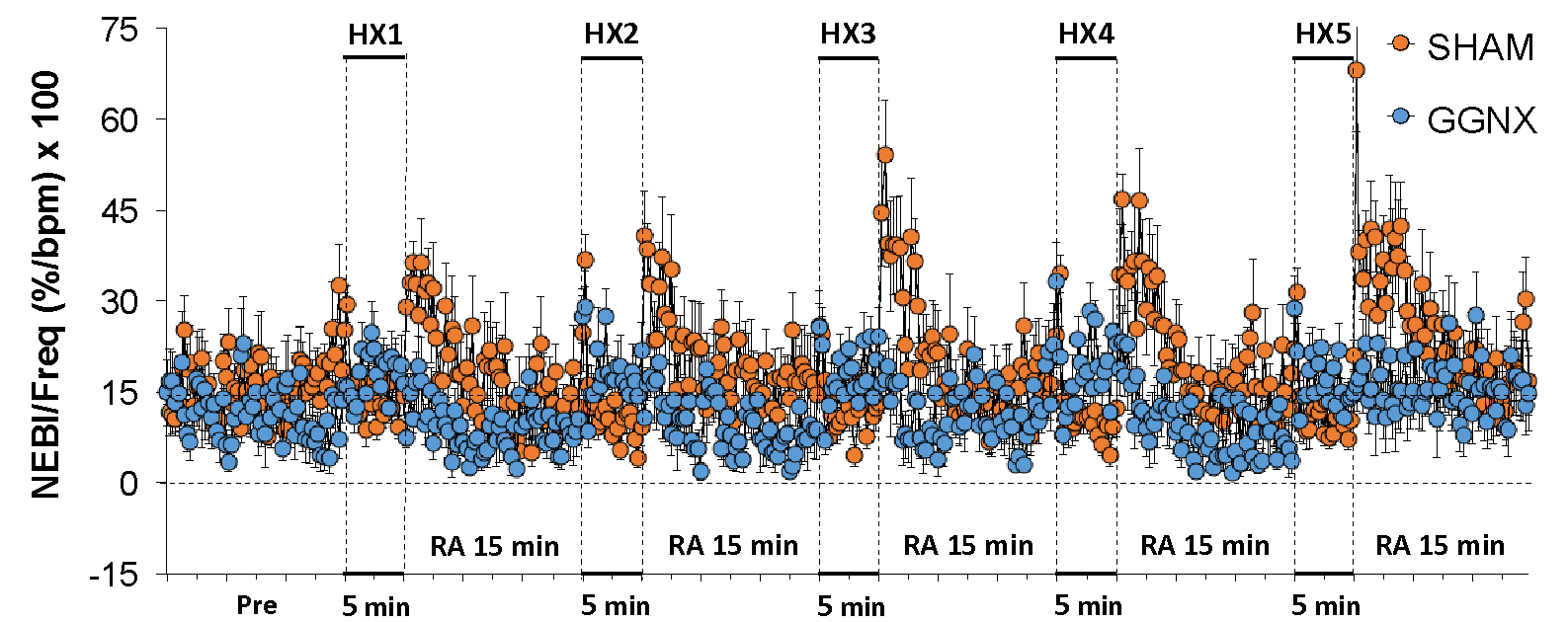


**B.**


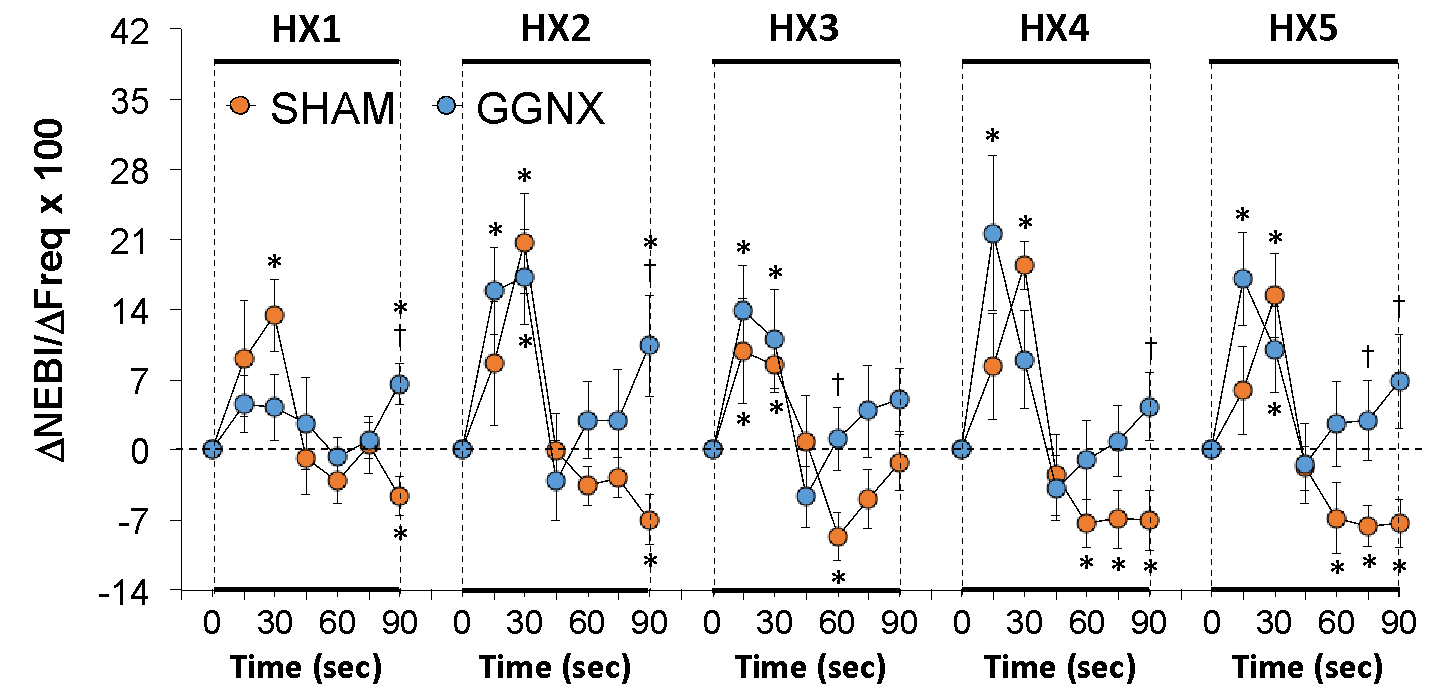


**D. 5 min totals**

**C. 90 sec totals**


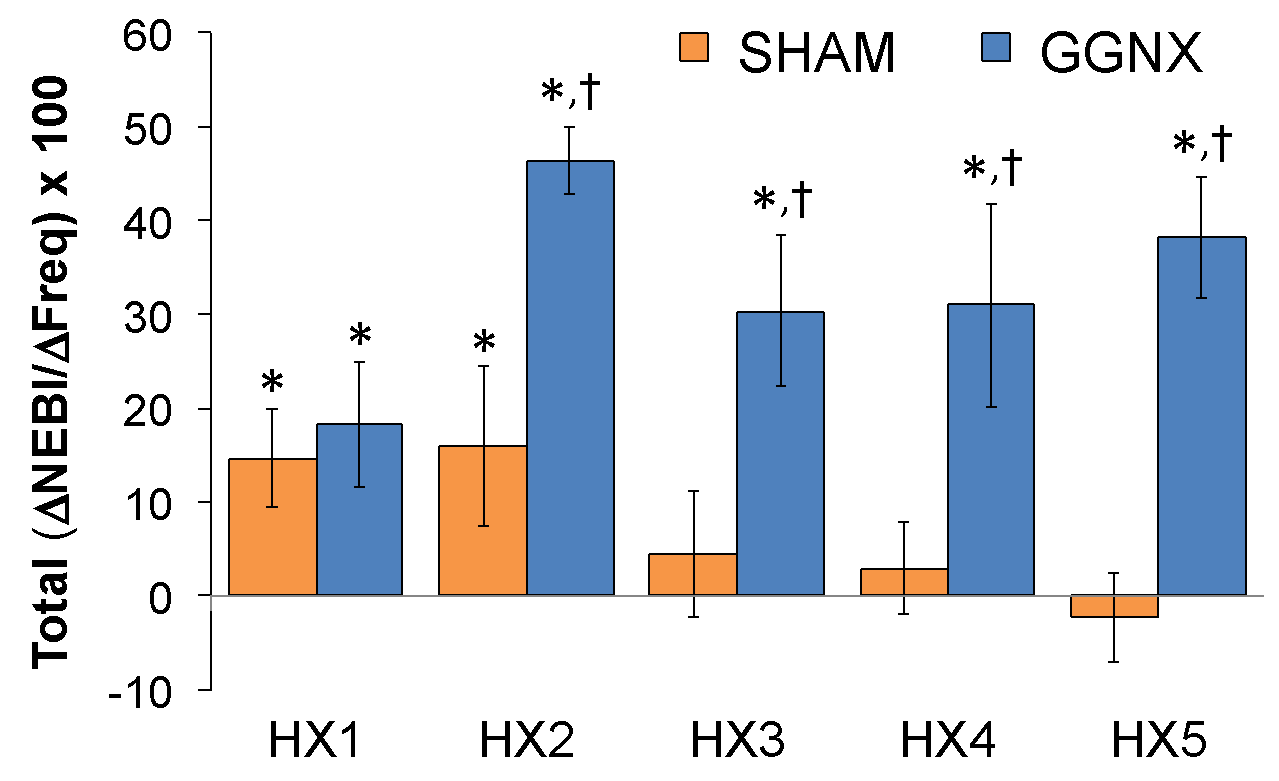

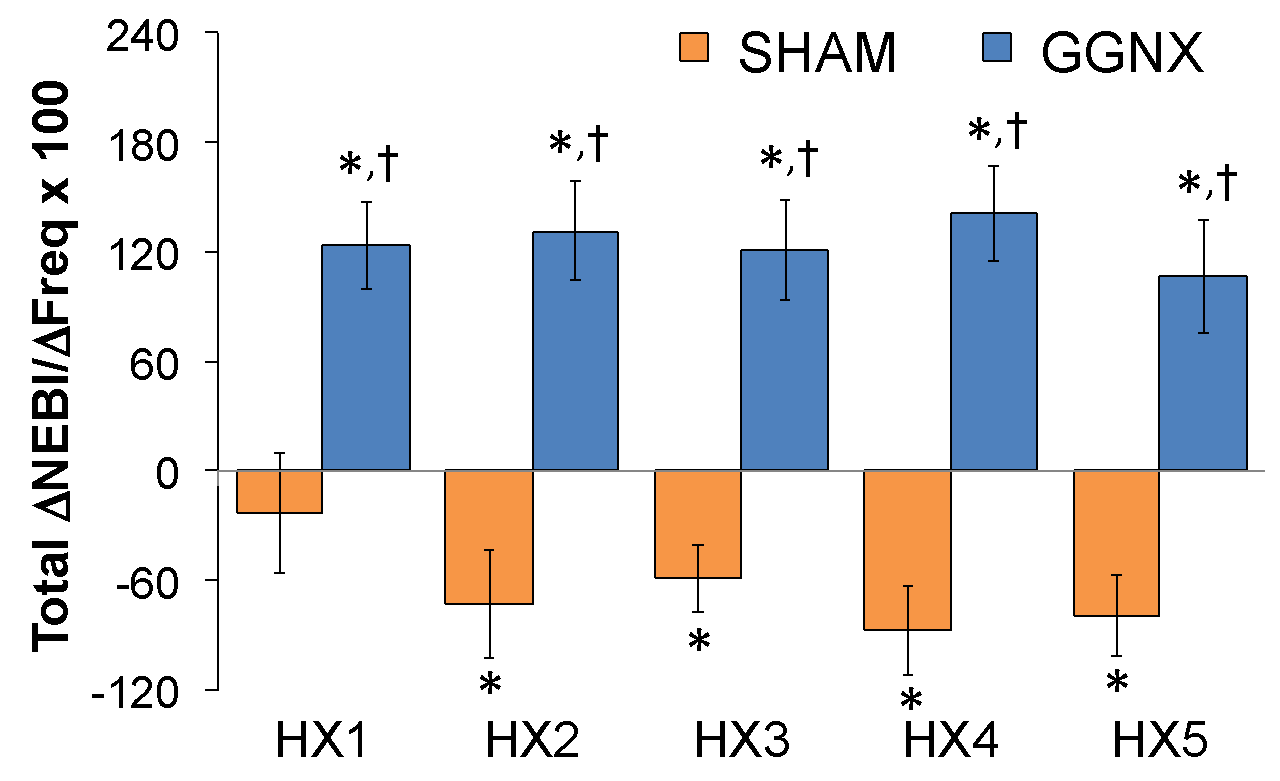


**Supplementary Figure S7. Panel A:** Non-eupneic breathing index (NEBI)/Frequency of breathing ratios in sham-operated (SHAM) rats and in rats with bilateral ganglioglomerular nerve transection (GGNX) before (Pre) and during five hypoxic (HX, 10% O_2_, 90% N_2_) gas challenges, each separated by 15 min of room-air (RA). **Panel B:** Arithmetic changes in frequency during the first 90 sec of HX gas challenge. Total changes in frequency during the first 90 sec (**Panel C**) and over the entire 5 min (**Panel D**) of HX gas challenge. The SHAM group had 10 rats. The GGNX group had 12 rats. The data are presented as mean ± SEM. **p* < 0.05, significant response. ^†^*p* < 0.05, GGNX rats *versus* SHAM rats.

**Supplementary Figure S8**



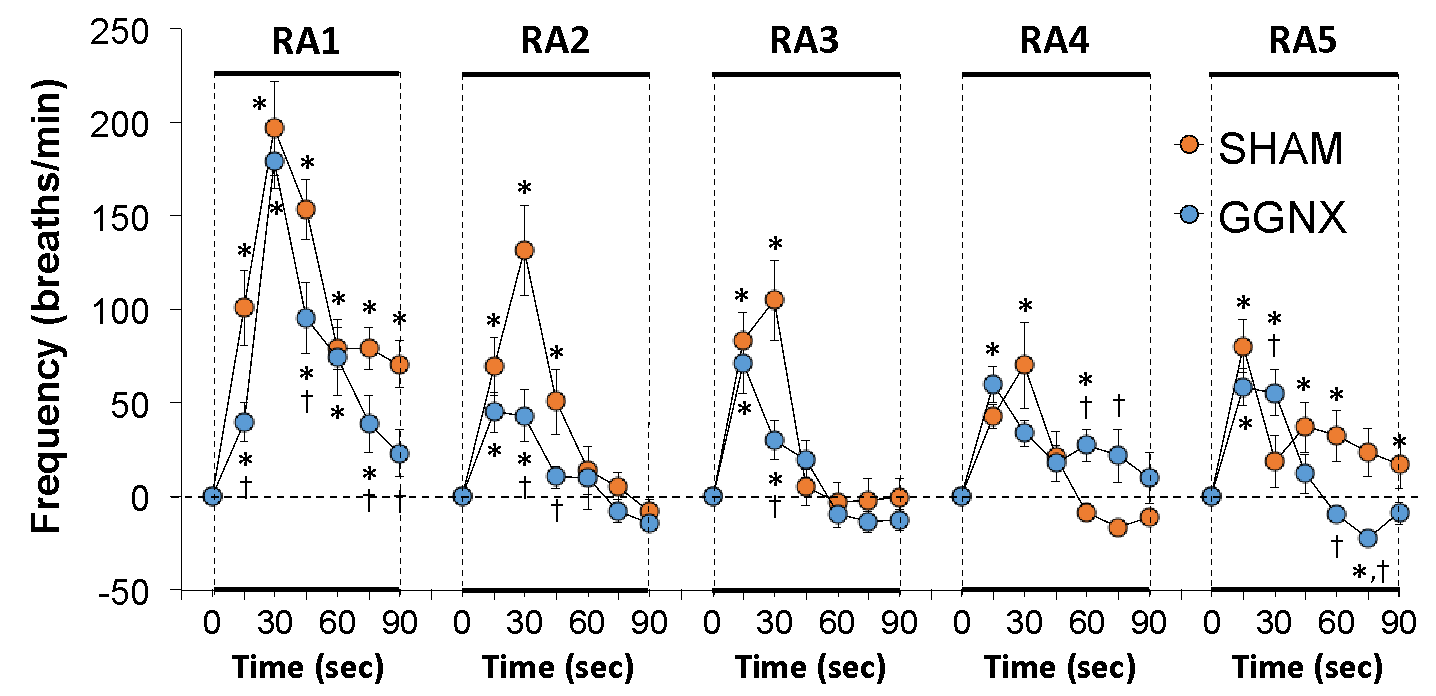


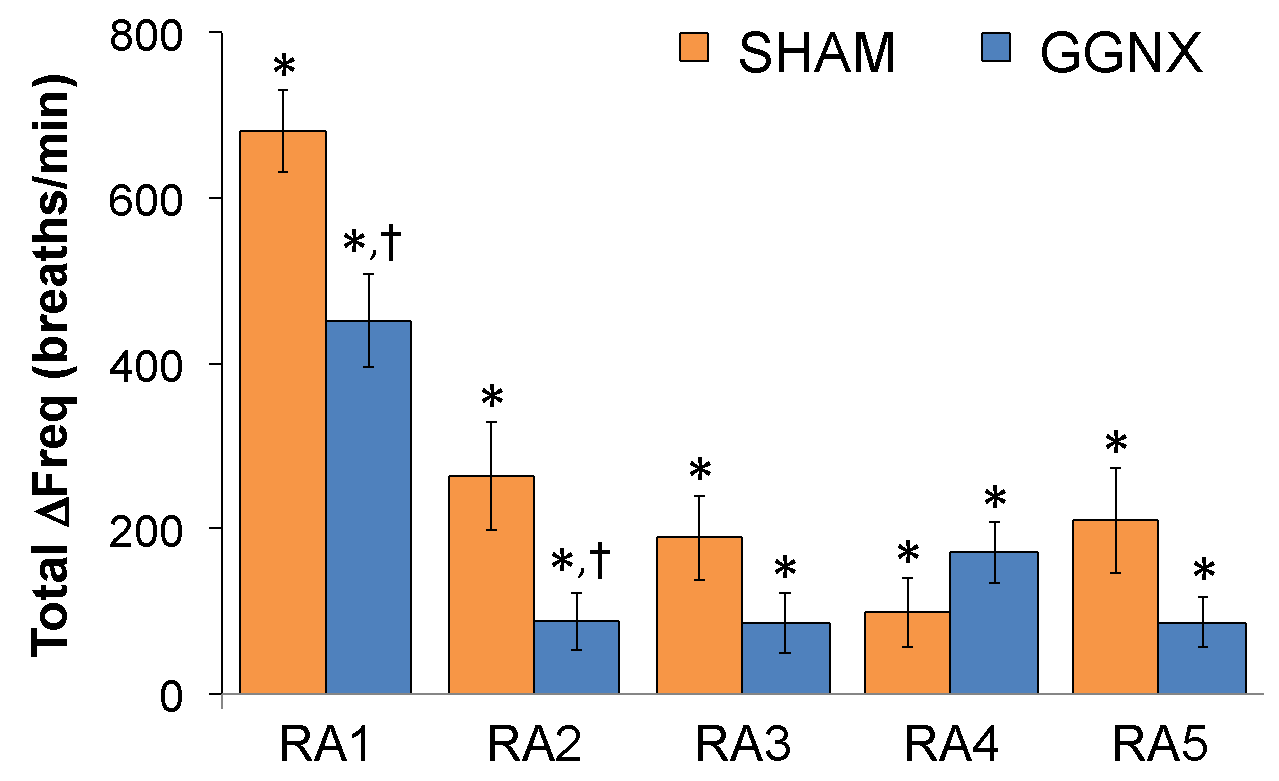

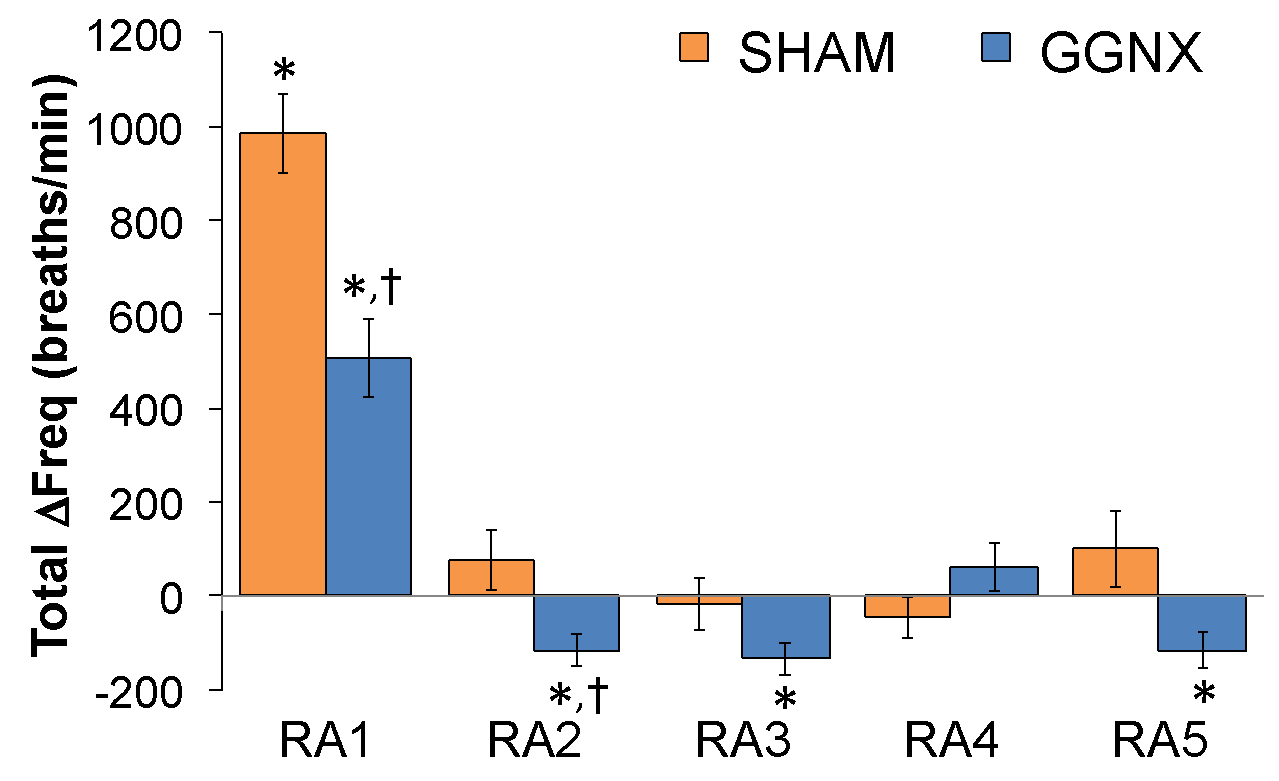


**Supplementary Figure S8. Panel A:** Arithmetic changes in frequency (Freq) of breathing from Pre-values in sham-operated (SHAM) rats and in rats with bilateral ganglioglomerular nerve transection (GGNX) during the first 90 seconds upon return to room-air (RA1-RA5), following the 5 hypoxic (HX, 10% O_2_, 90% N_2_) gas challenges. **Panel B:** Total arithmetic changes in Freq (ΔFreq) during the first 90 sec of the return to room-air phases (RA1-RA5). **Panel C:** Total arithmetic changes in Freq (ΔFreq) during the first 5 min of the return to room-air phases (RA1-RA5). The SHAM group had 10 rats. The GGNX group had 12 rats. The data are presented as mean ± SEM. **p* < 0.05, significant response. ^†^*p* < 0.05, GGNX rats *versus* SHAM rats.

**Supplementary Figure S9**



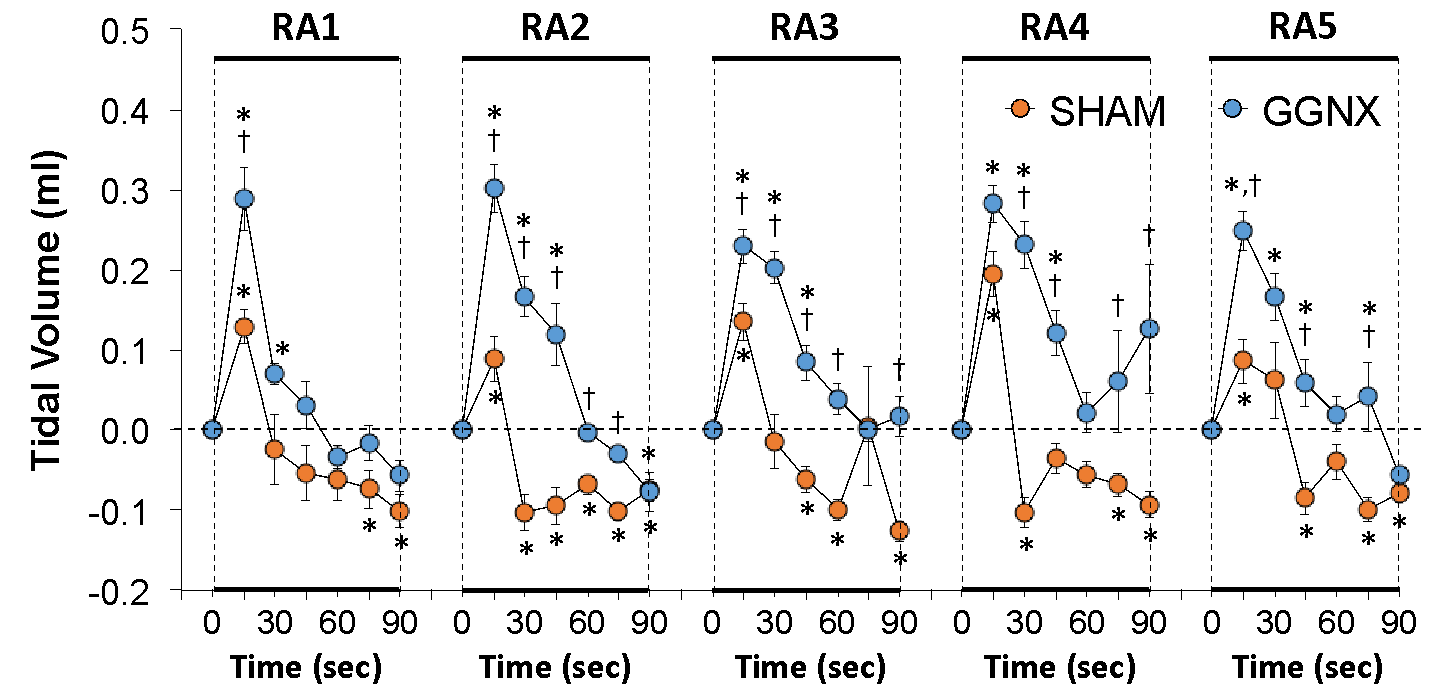


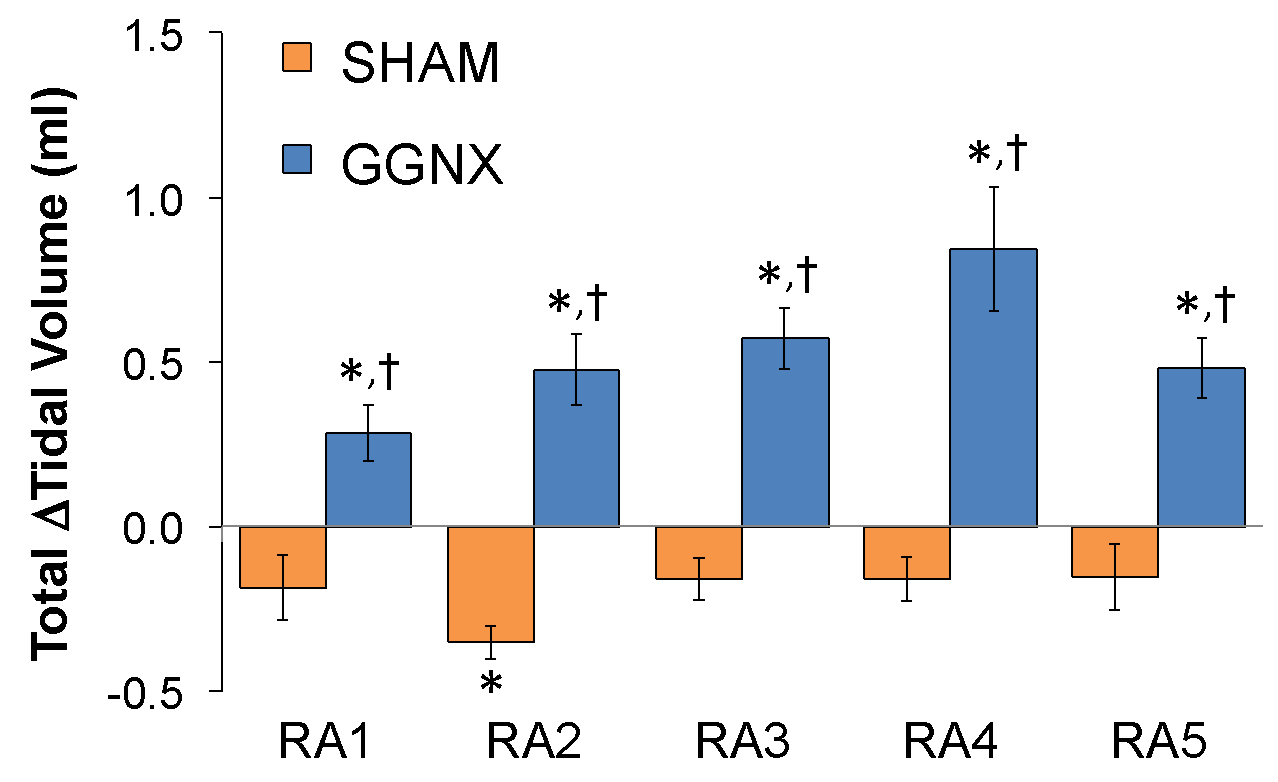

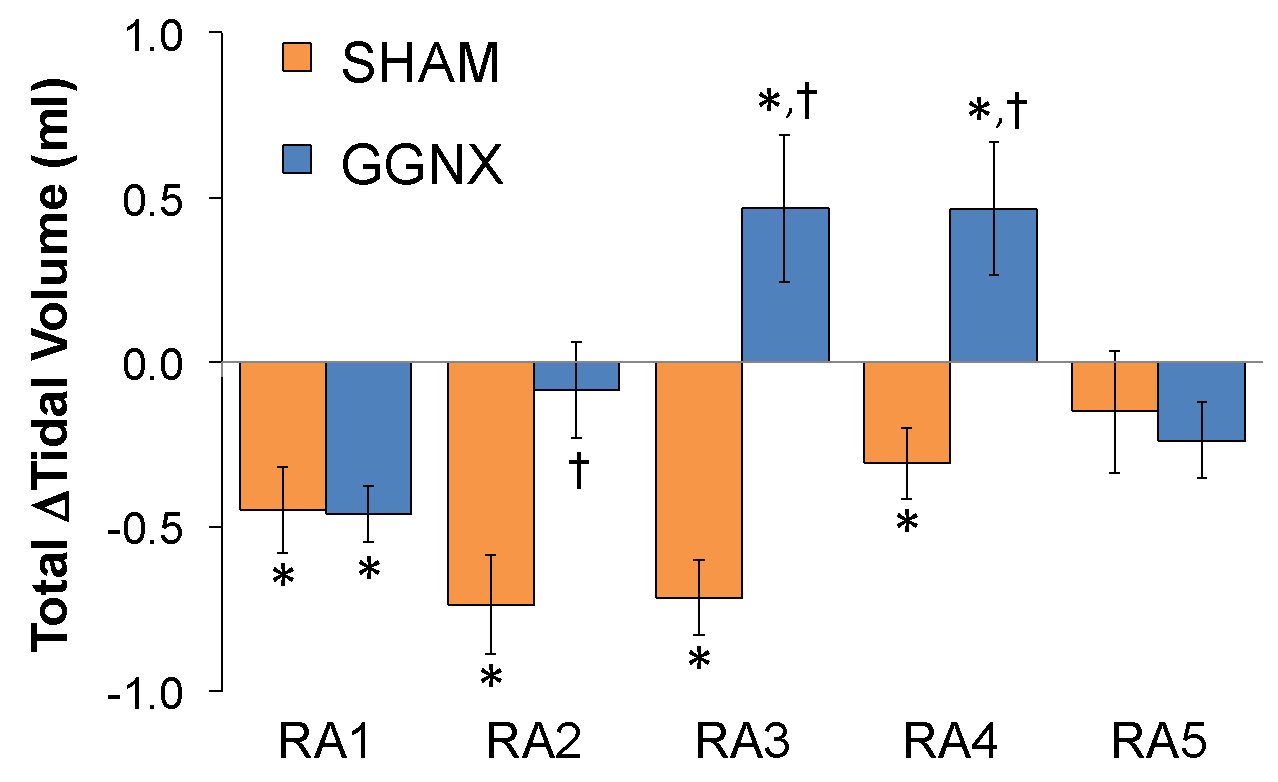


**Supplementary Figure S9. Panel A:** Arithmetic changes in tidal volume from Pre-values in sham-operated (SHAM) rats and in rats with bilateral ganglioglomerular nerve transection (GGNX) during the first 90 seconds upon return to room-air (RA1-RA5), following the 5 hypoxic (HX, 10% O_2_, 90% N_2_) gas challenges. **Panel B:** Total arithmetic changes in tidal volume during the first 90 sec of the return to room-air phases (RA1-RA5). **Panel C:** Total arithmetic changes in tidal volume during the first 5 min of the return to room-air phases (RA1-RA5). The SHAM group had 10 rats. The GGNX group had 12 rats. The data are presented as mean ± SEM. **p* < 0.05, significant response. ^†^*p* < 0.05, GGNX rats *versus* SHAM rats.

**Supplementary Figure S10**



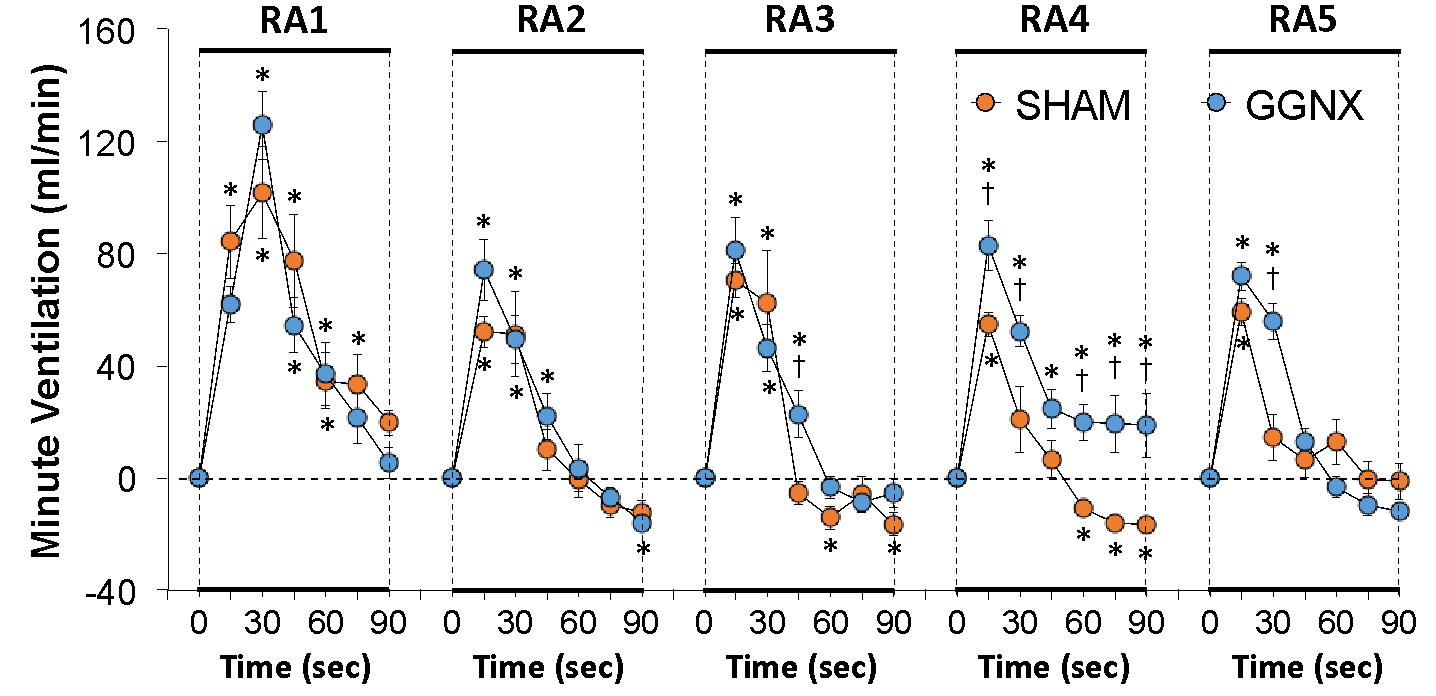


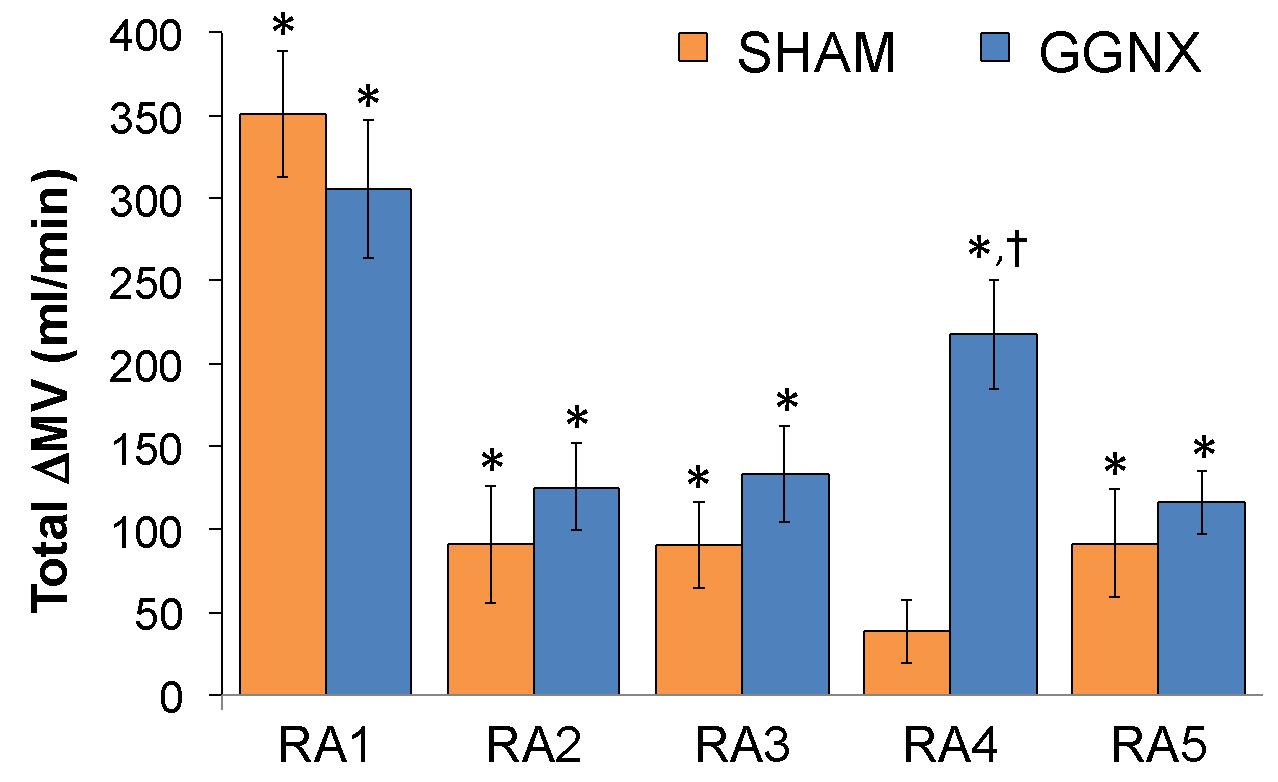

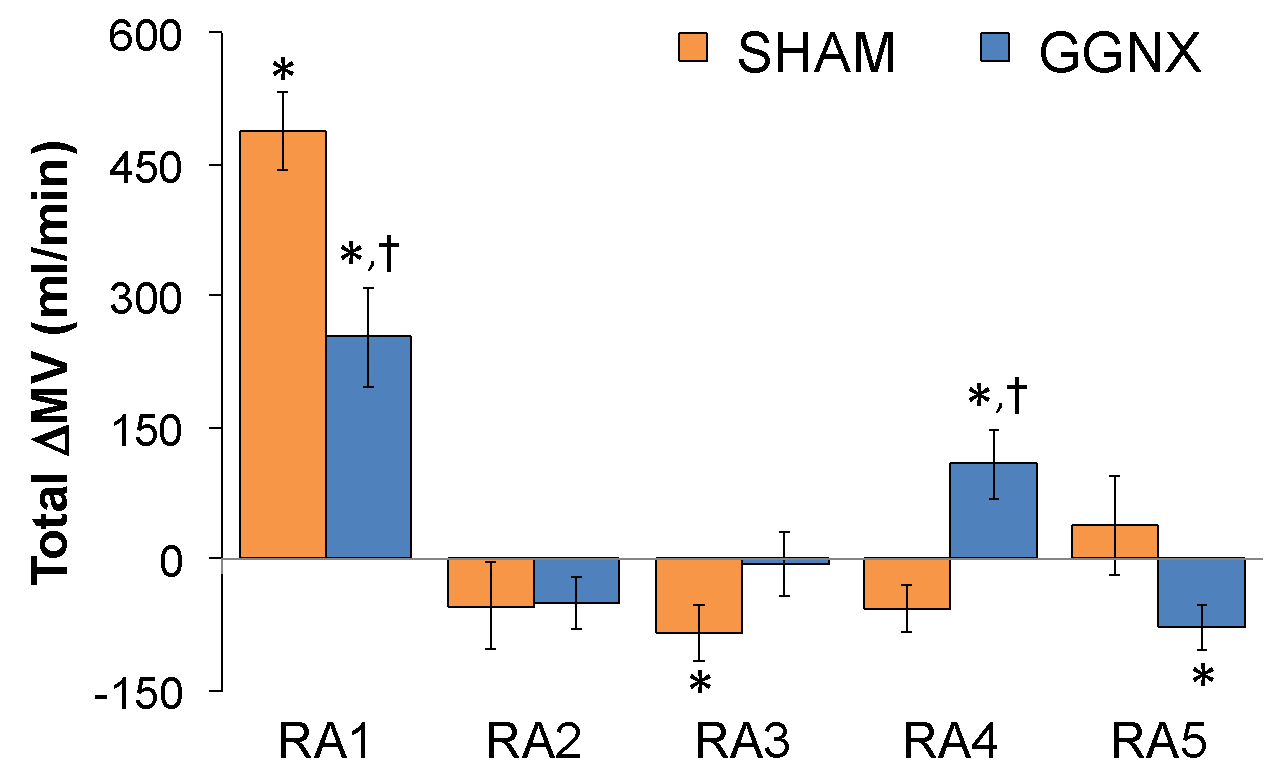


**Supplementary Figure S10. Panel A:** Arithmetic changes in minute ventilation (MV) from Pre-values in sham-operated (SHAM) rats and in rats with bilateral ganglioglomerular nerve transection (GGNX) during the first 90 seconds upon return to room-air (RA1-RA5), following the 5 hypoxic (HX, 10% O_2_, 90% N_2_) gas challenges. **Panel B:** Total arithmetic changes in MV (ΔMV) during the first 90 sec of the return to room-air phases (RA1-RA5). **Panel C:** Total arithmetic changes in MV (ΔMV) during the first 5 min of the return to room-air phases (RA1-RA5). The SHAM group had 10 rats. The GGNX group had 12 rats. The data are presented as mean ± SEM. **p* < 0.05, significant response. ^†^*p* < 0.05, GGNX rats *versus* SHAM rats.

**Supplementary Figure S11**



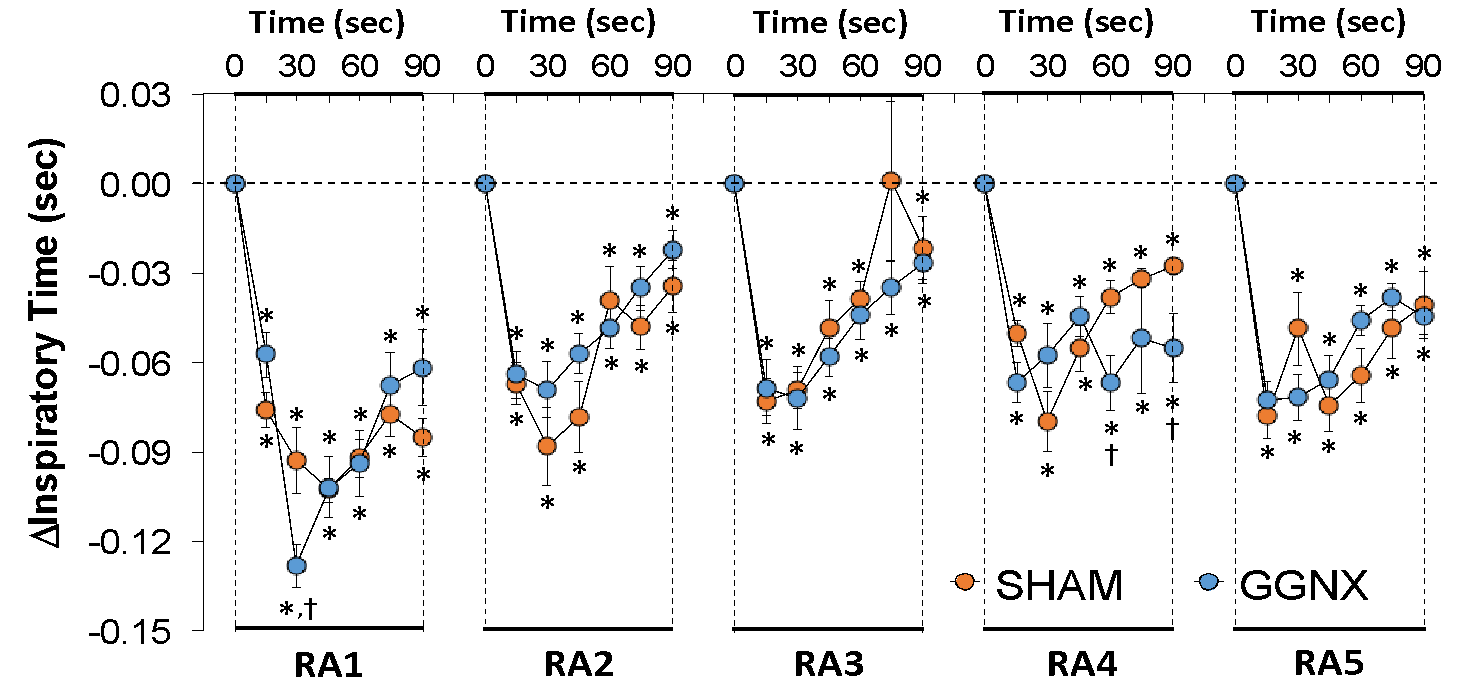


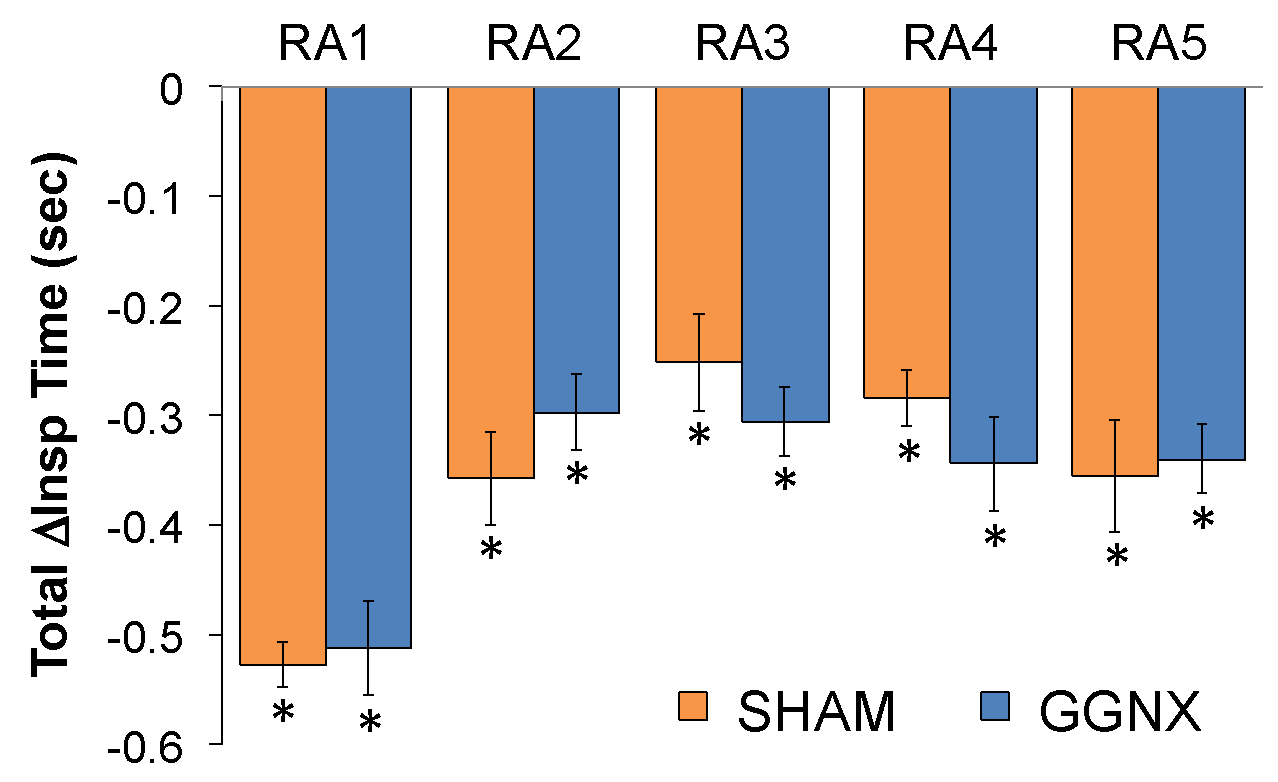


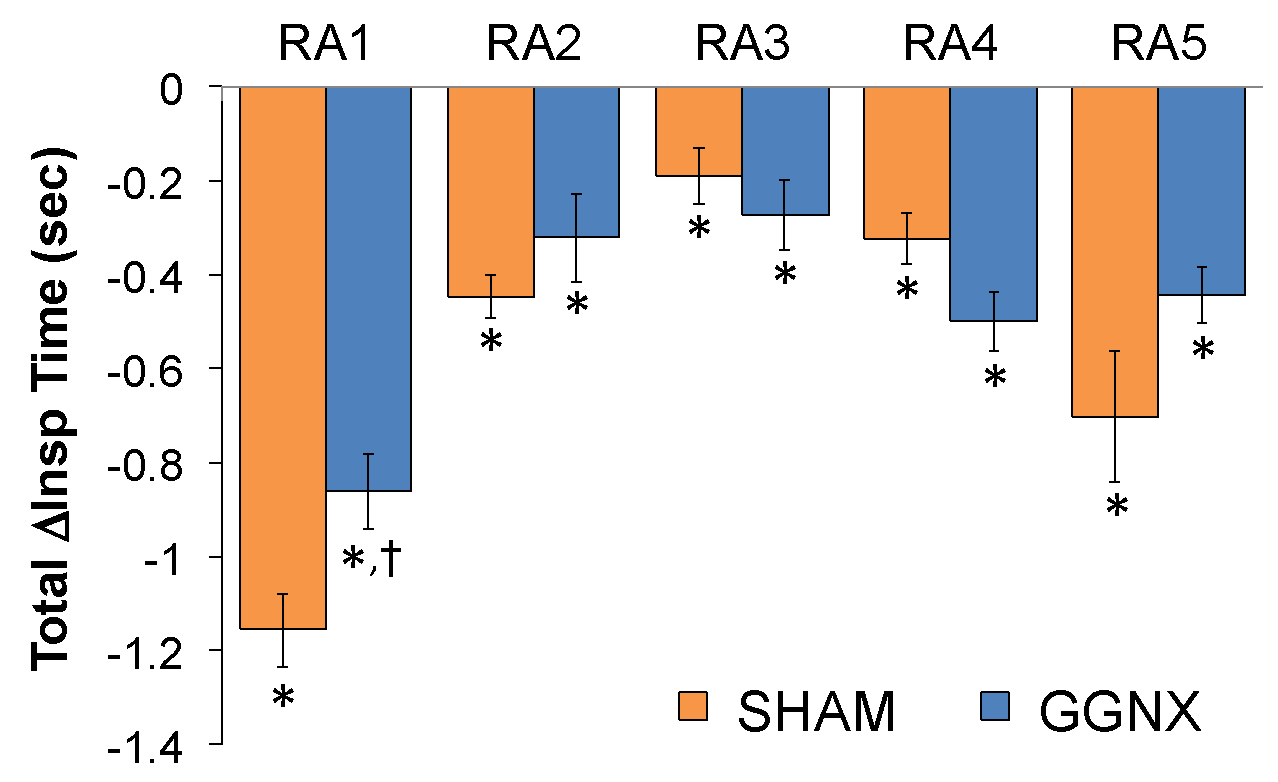


**Supplementary Figure S11. Panel A:** Arithmetic changes in Inspiratory Time (Insp Time) from Pre-values in sham-operated (SHAM) rats and in rats with bilateral ganglioglomerular nerve transection (GGNX) during the first 90 seconds upon return to room-air (RA1-RA5), following the 5 hypoxic (HX, 10% O_2_, 90% N_2_) gas challenges. **Panel B:** Total arithmetic changes in Insp Time (ΔInsp Time) during the first 90 sec of the return to room-air phases (RA1-RA5). **Panel C:** Total arithmetic changes in Insp Time (ΔInsp Time) during the first 5 min of the return to room-air phases (RA1-RA5). The SHAM group had 10 rats. The GGNX group had 12 rats. The data are presented as mean ± SEM. **p* < 0.05, significant response. ^†^*p* < 0.05, GGNX rats *versus* SHAM rats.

**Supplementary Figure S12**



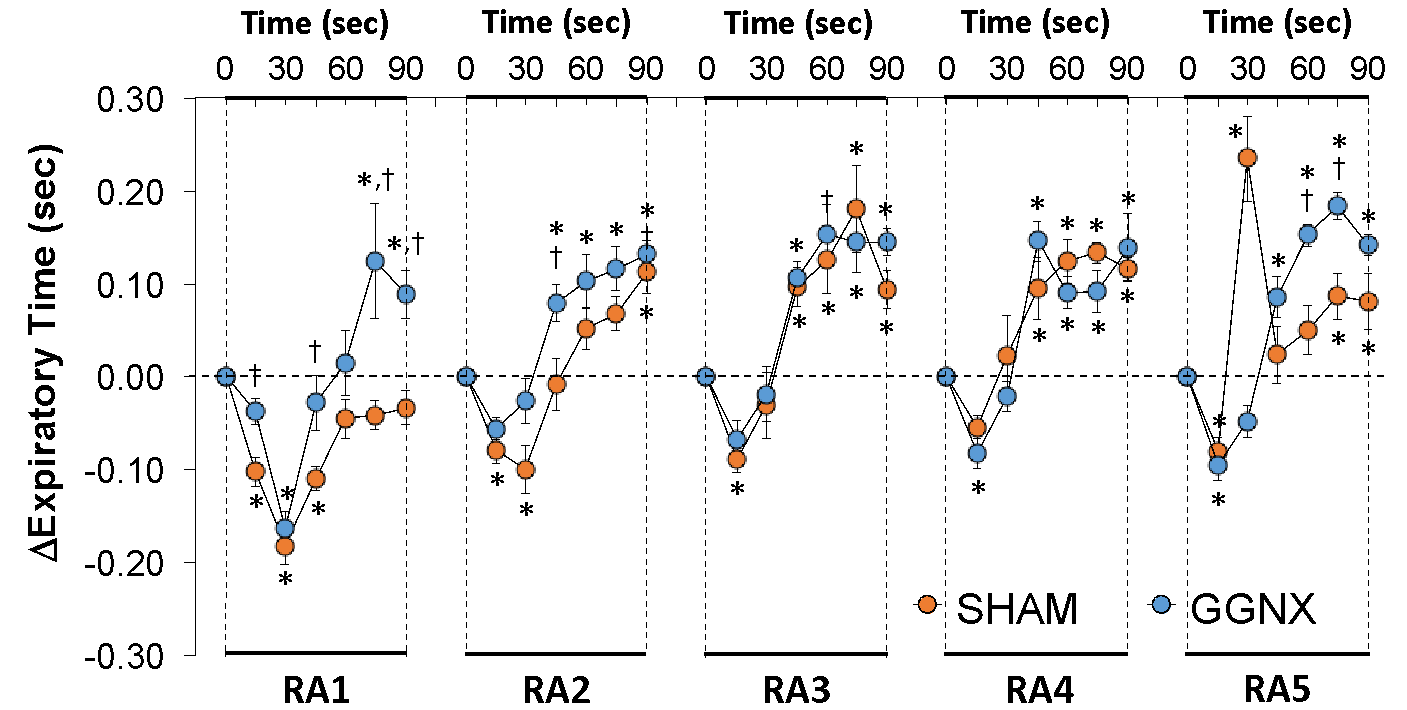


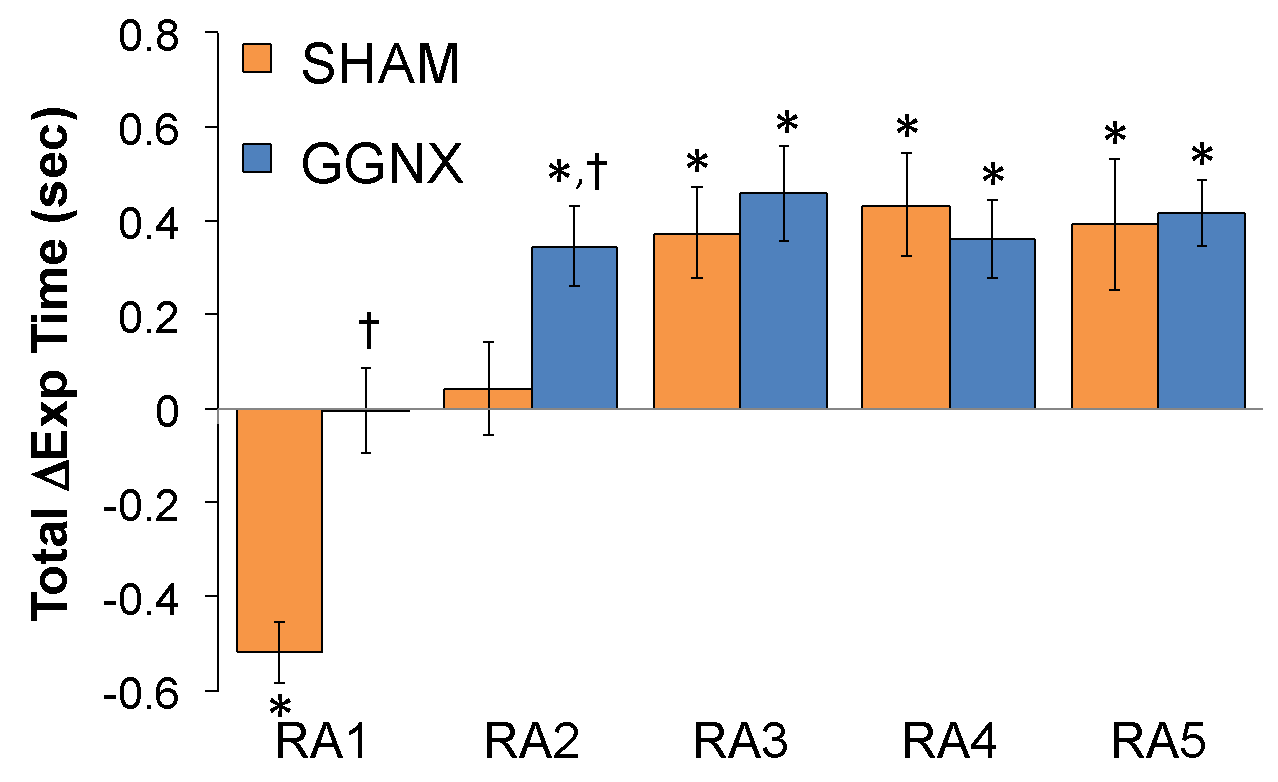

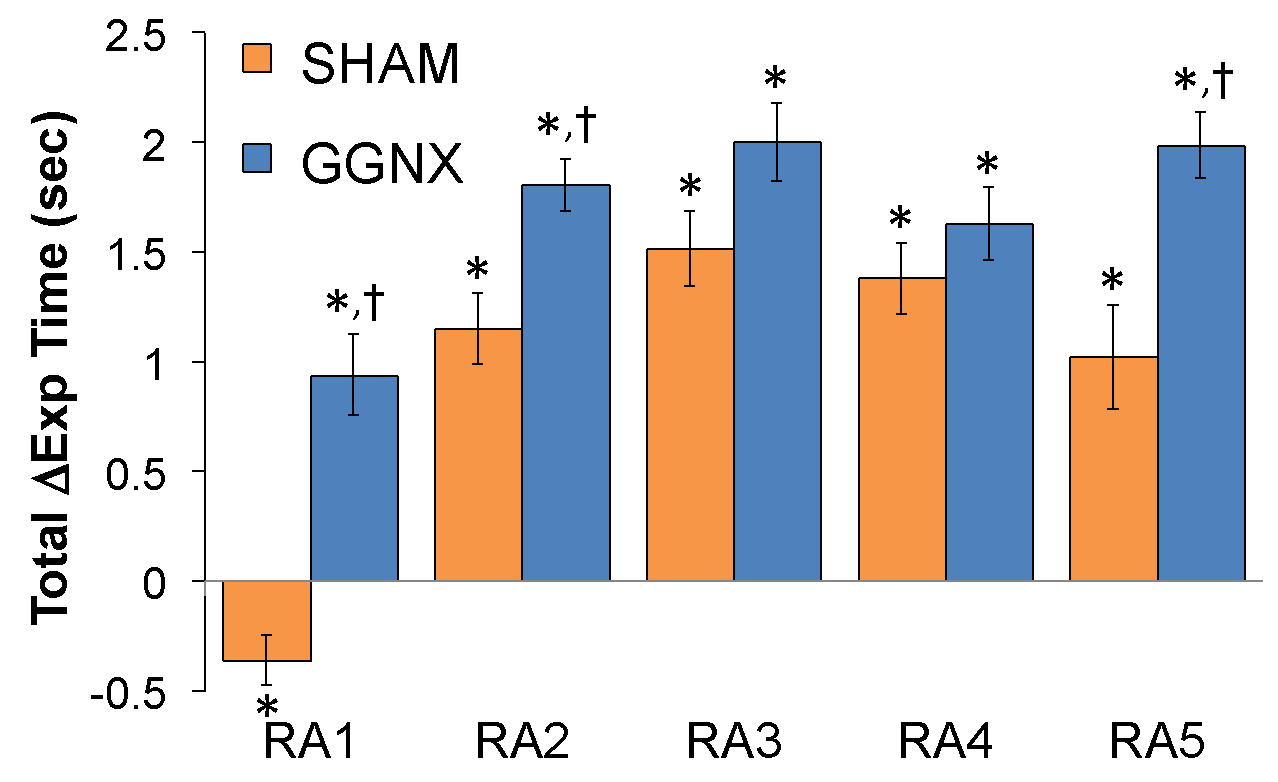


**Supplementary Figure S12. Panel A:** Arithmetic changes in Expiratory Time (Exp Time) from Pre-values in sham-operated (SHAM) rats and in rats with bilateral ganglioglomerular nerve transection (GGNX) during the first 90 seconds upon return to room-air (RA1-RA5), following the 5 hypoxic (HX, 10% O_2_, 90% N_2_) gas challenges. **Panel B:** Total arithmetic changes in Exp Time (ΔExp Time) during the first 90 sec of the return to room-air phases (RA1-RA5). **Panel C:** Total arithmetic changes in Exp Time (ΔExp Time) during the first 5 min of the return to room-air phases (RA1-RA5). The SHAM group had 10 rats. The GGNX group had 12 rats. The data are presented as mean ± SEM. **p* < 0.05, significant response. ^†^*p* < 0.05, GGNX rats *versus* SHAM rats.

**Supplementary Figure S13**



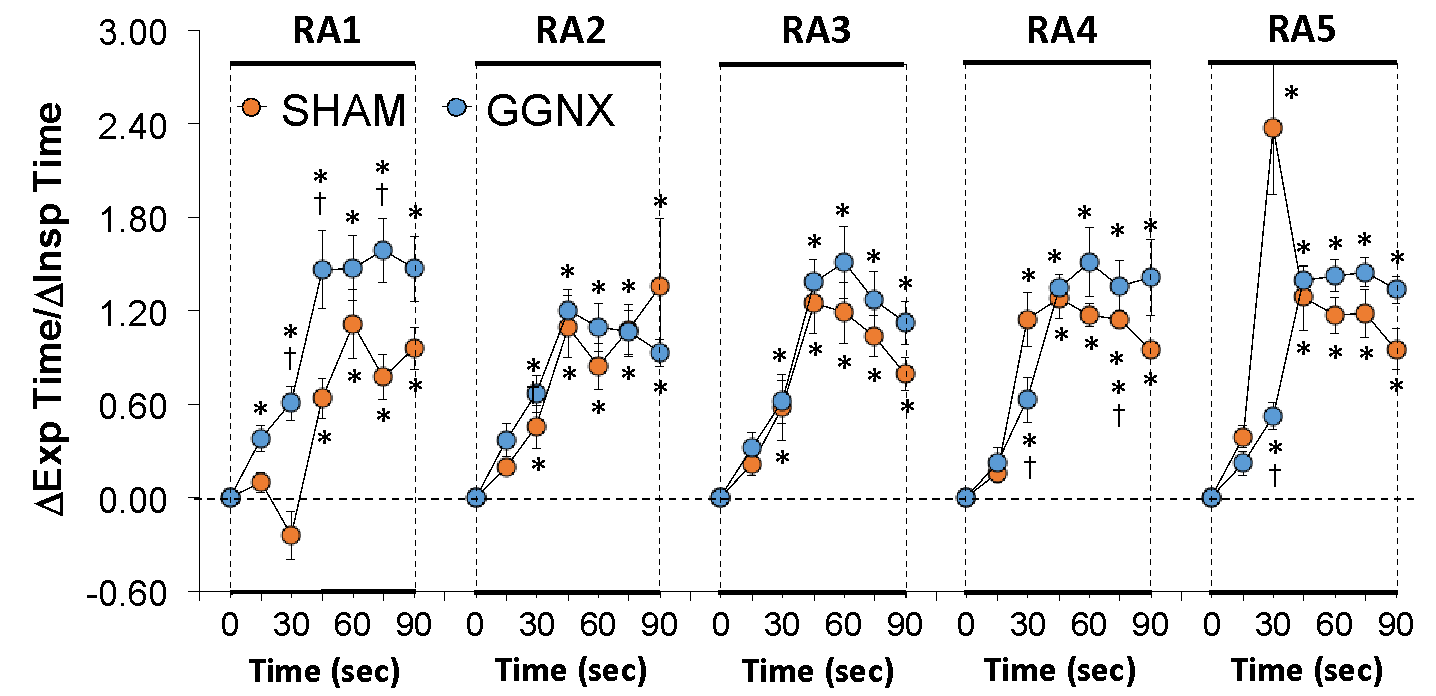


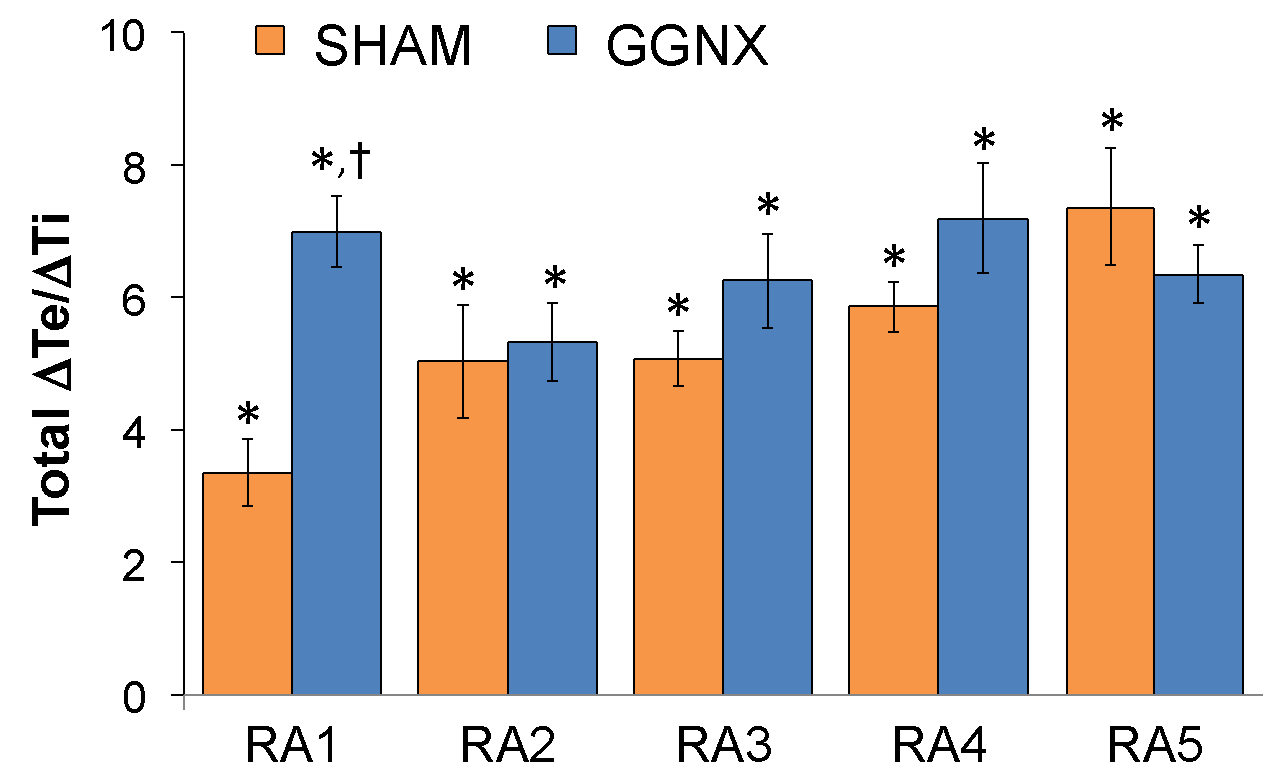

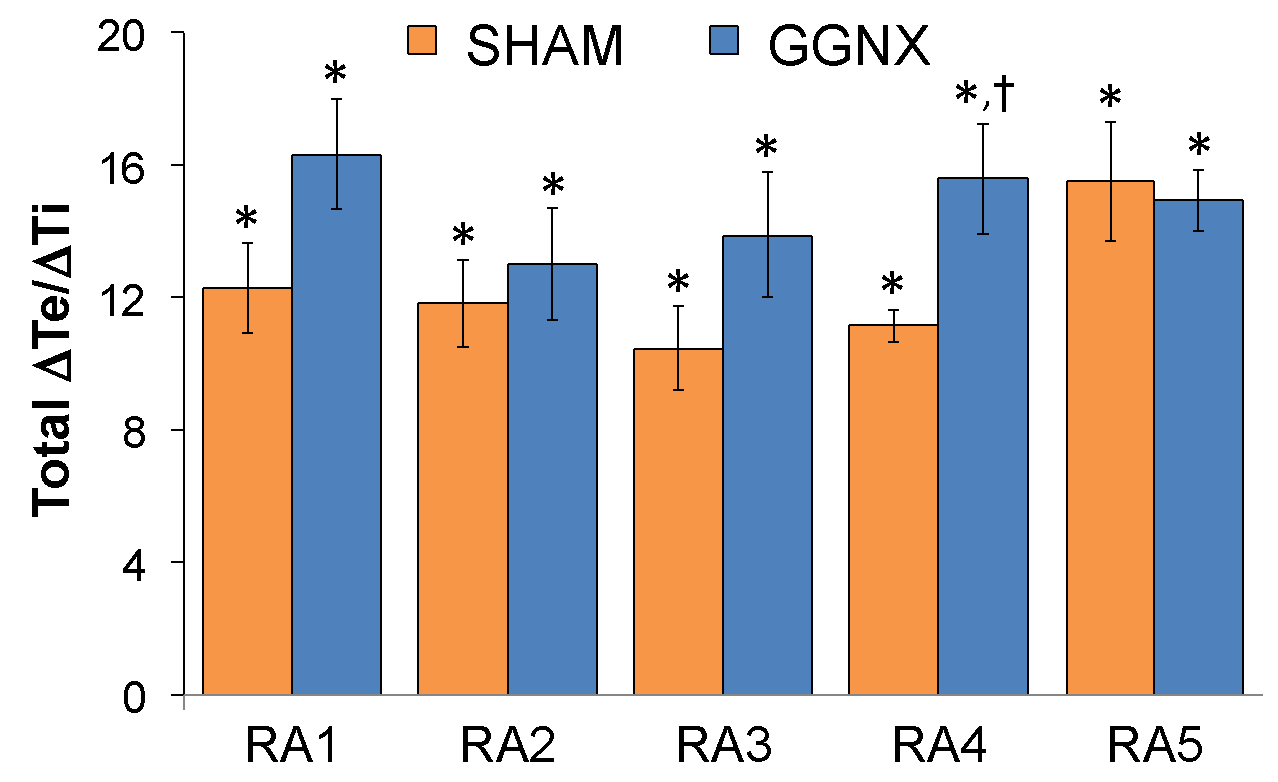


**Supplementary Figure S13. Panel A:** Arithmetic changes in Expiratory Time/Inspiratory Time (Exp Time/Insp Time, Te/Ti) from Pre-values in sham-operated (SHAM) rats and in rats with bilateral ganglioglomerular nerve transection (GGNX) during the first 90 seconds upon return to room-air (RA1-RA5), following the 5 hypoxic (HX, 10% O_2_, 90% N_2_) gas challenges. **Panel B:** Total arithmetic changes in Te/Ti (ΔTe/ΔTi) during the first 90 sec of the return to room-air phases (RA1-RA5). **Panel C:** Total arithmetic changes in Te/Ti (ΔTe/ΔTi) during the first 5 min of the return to room-air phases (RA1-RA5). The SHAM group had 10 rats. The GGNX group had 12 rats. The data are presented as mean ± SEM. **p* < 0.05, significant response. ^†^*p* < 0.05, GGNX rats *versus* SHAM rats.

**Supplementary Figure S14**


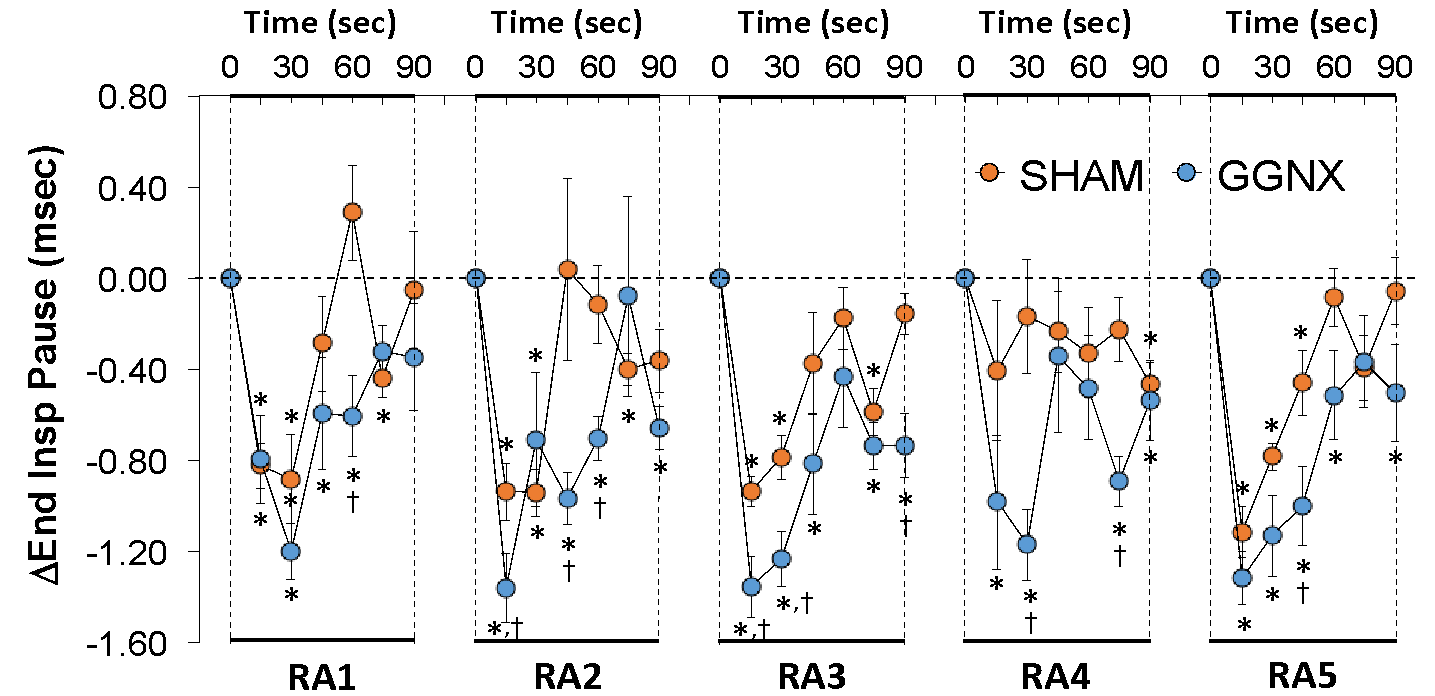


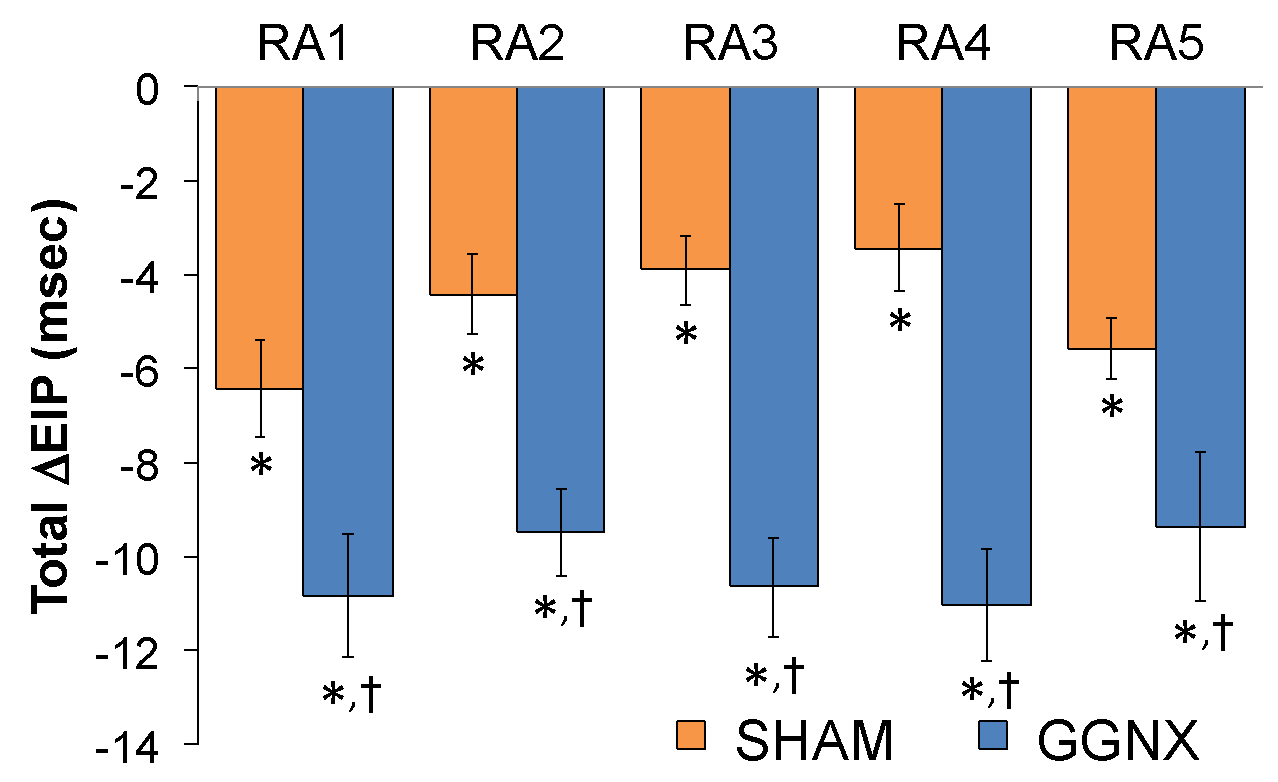


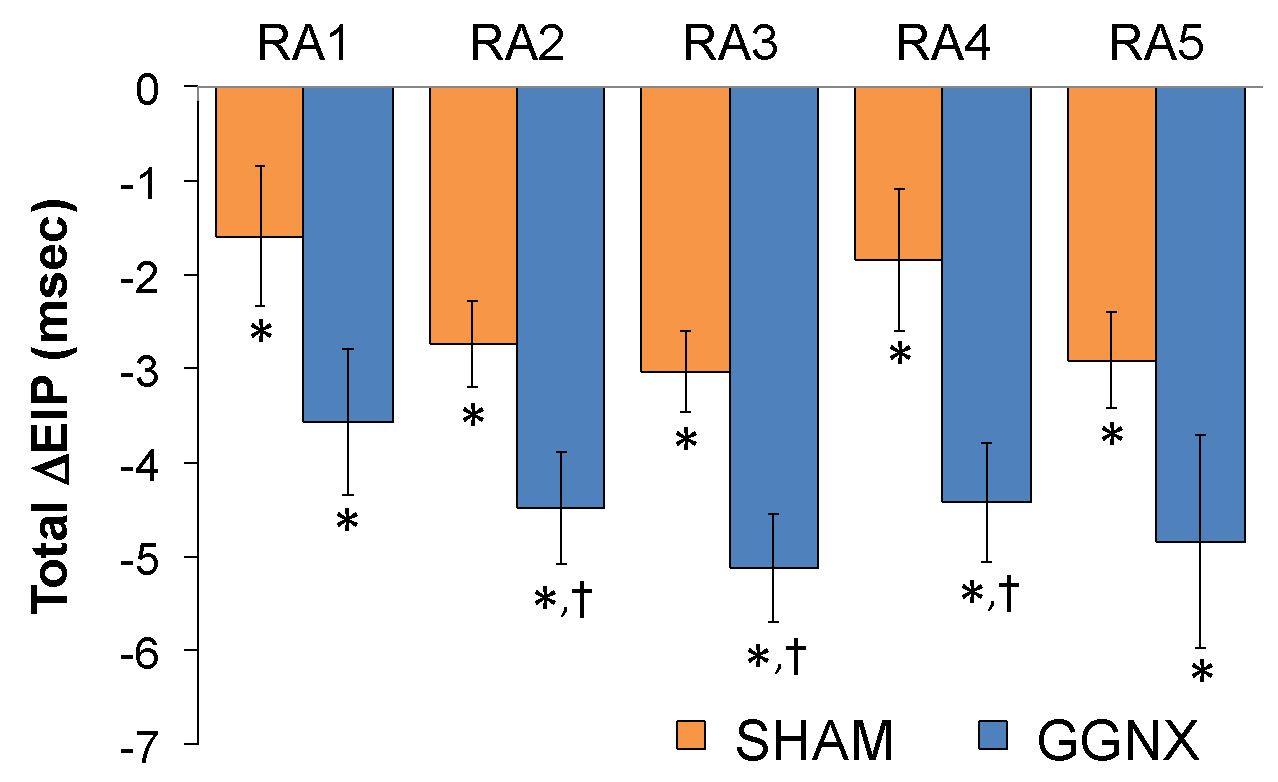


**Supplementary Figure S14. Panel A:** Arithmetic changes in End Inspiratory Pause (End Insp Pause, EIP) from Pre-values in sham-operated (SHAM) rats and in rats with bilateral ganglioglomerular nerve transection (GGNX) during the first 90 seconds upon return to room-air (RA1-RA5), following the 5 hypoxic (HX, 10% O_2_, 90% N_2_) gas challenges. **Panel B:** Total arithmetic changes in EIP (ΔEIP) during the first 90 sec of the return to room-air phases (RA1-RA5). **Panel C:** Total arithmetic changes in EIP (ΔEIP) during the first 5 min of the return to room-air phases (RA1-RA5). The SHAM group had 10 rats. The GGNX group had 12 rats. The data are presented as mean ± SEM. **p* < 0.05, significant response. ^†^*p* < 0.05, GGNX rats *versus* SHAM rats.

**Supplementary Figure S15**



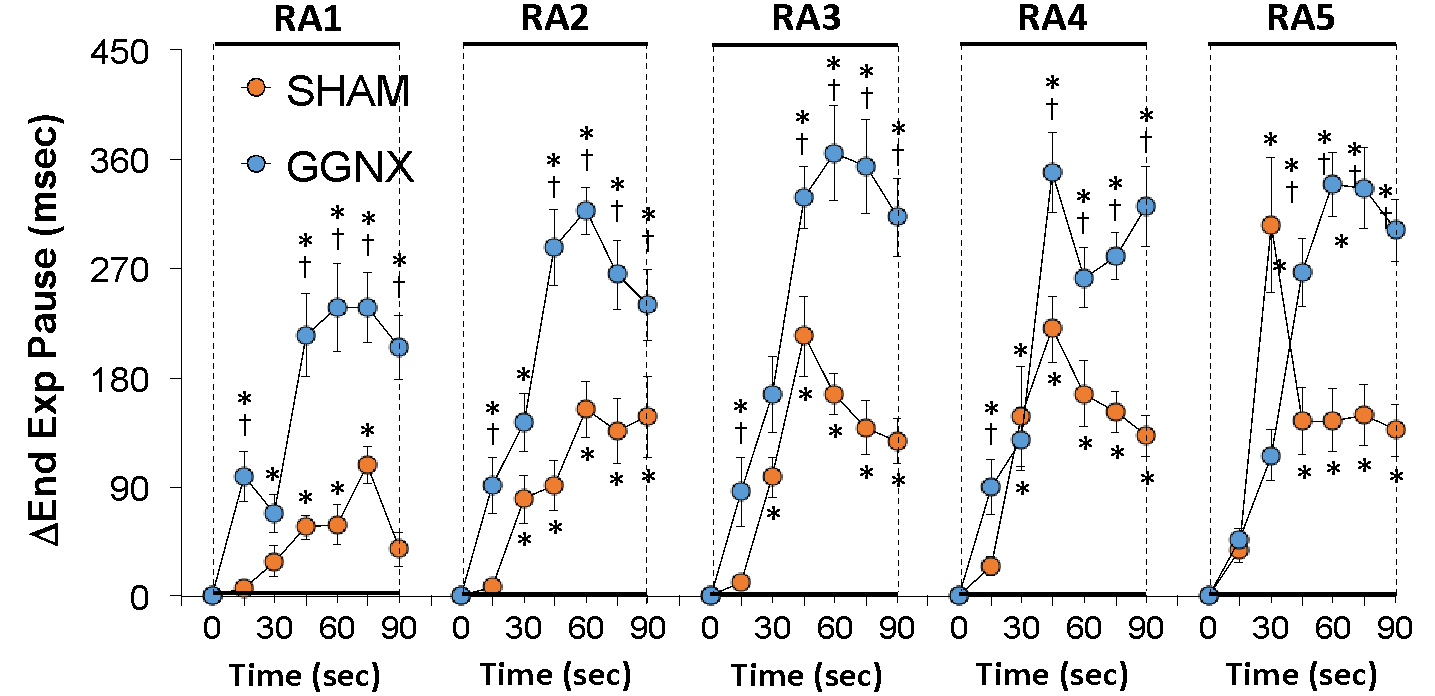


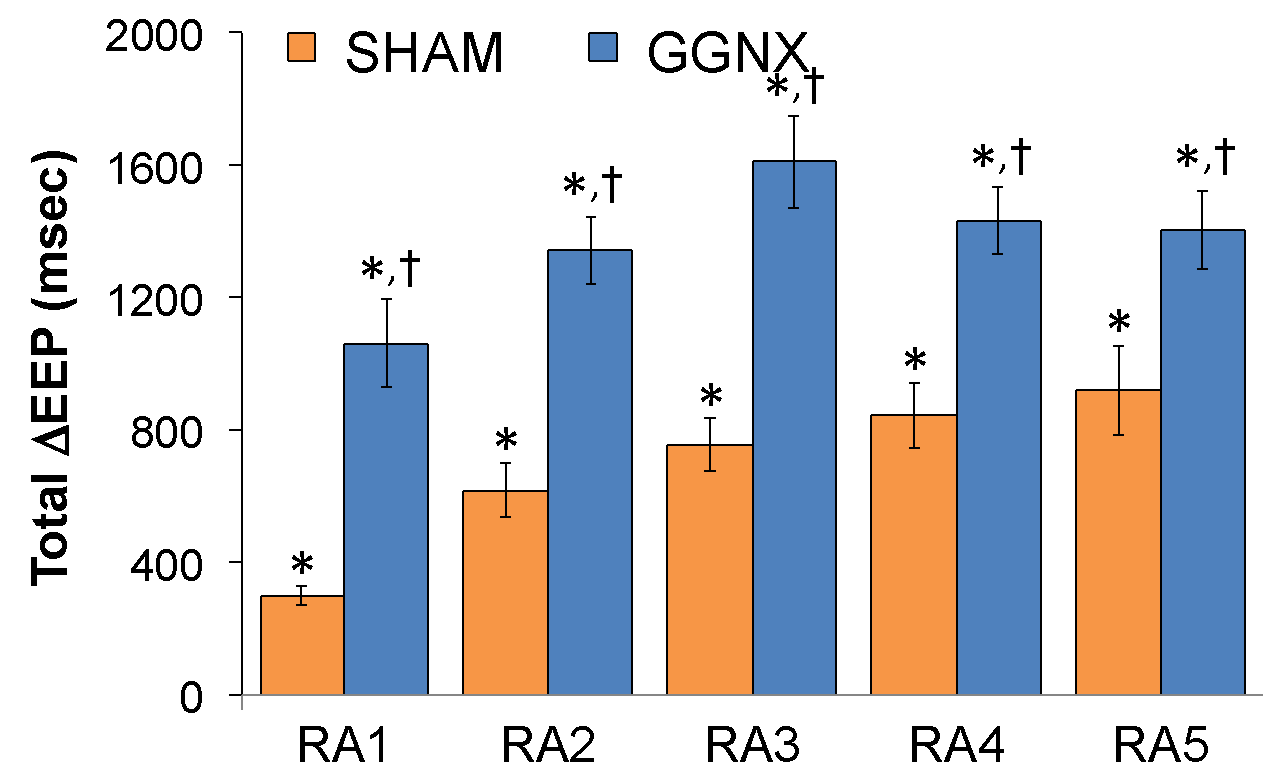

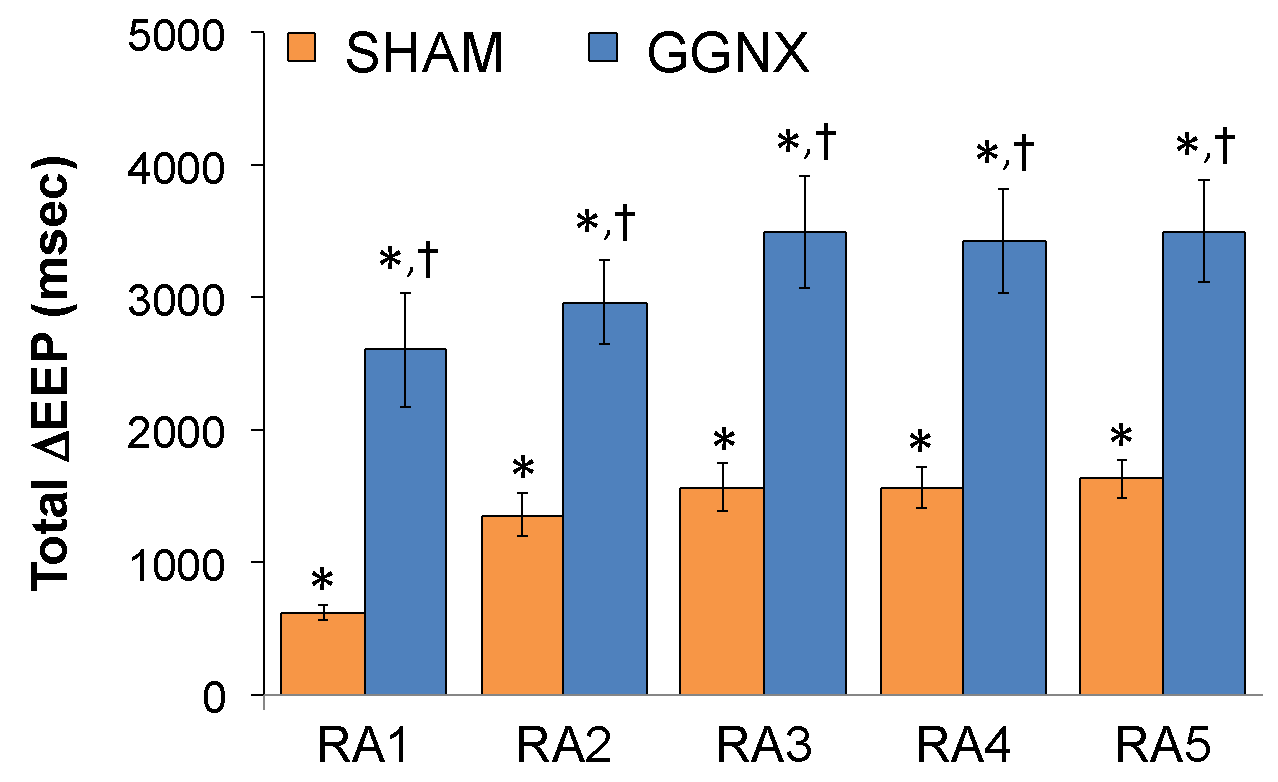


**Supplementary Figure S15. Panel A:** Arithmetic changes in End Expiratory Pause (End Exp Pause, EEP) from Pre-values in sham-operated (SHAM) rats and in rats with bilateral ganglioglomerular nerve transection (GGNX) during the first 90 seconds upon return to room-air (RA1-RA5), following the 5 hypoxic (HX, 10% O_2_, 90% N_2_) gas challenges. **Panel B:** Total arithmetic changes in EEP (ΔEEP) during the first 90 sec of the return to room-air phases (RA1-RA5). **Panel C:** Total arithmetic changes in EEP(ΔEEP) during the first 5 min of the return to room-air phases (RA1-RA5). The SHAM group had 10 rats. The GGNX group had 12 rats. The data are presented as mean ± SEM. **p* < 0.05, significant response. ^†^*p* < 0.05, GGNX rats *versus* SHAM rats.

**Supplementary Figure S16**



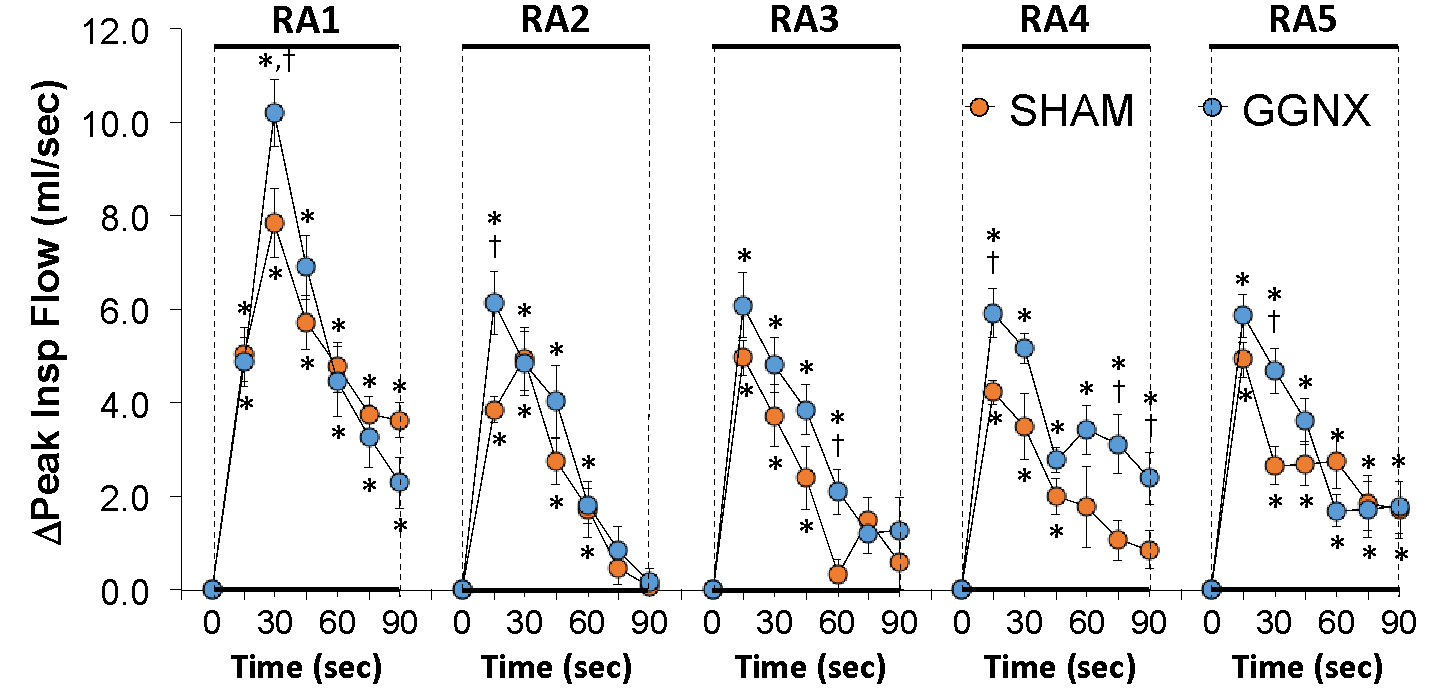


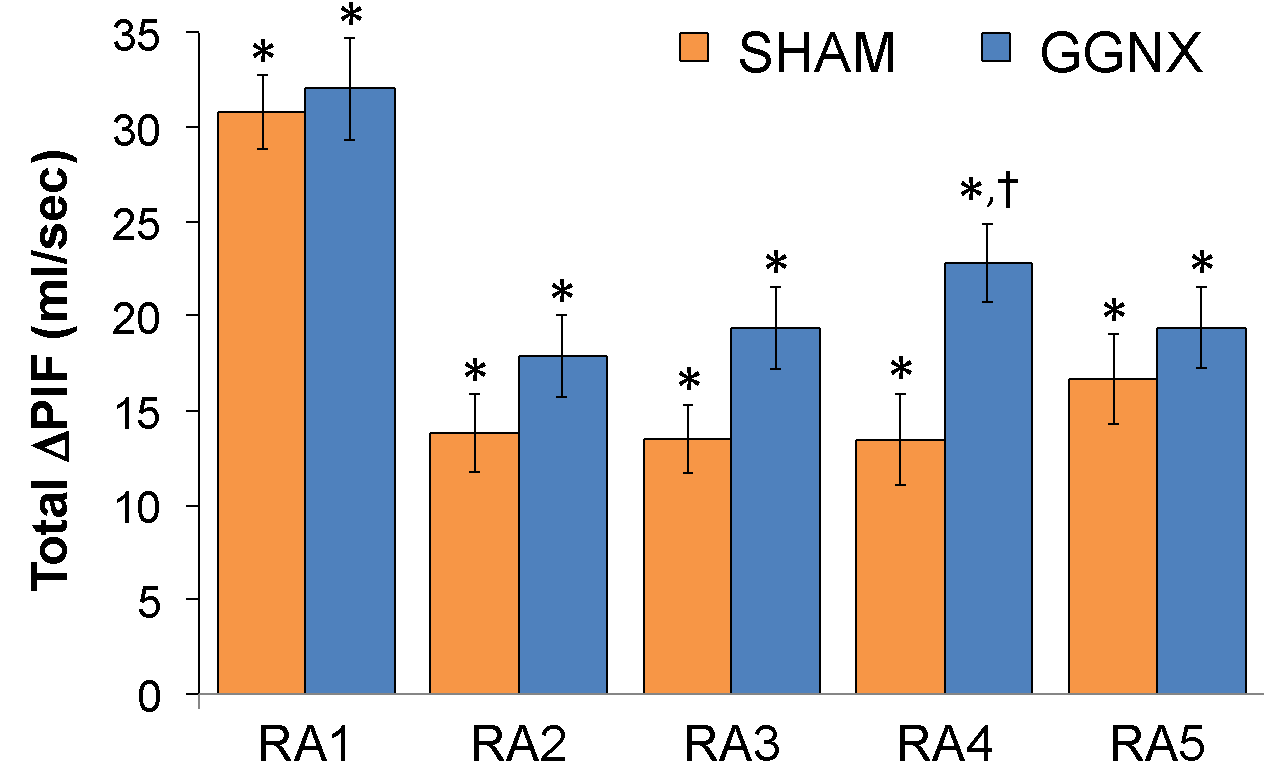

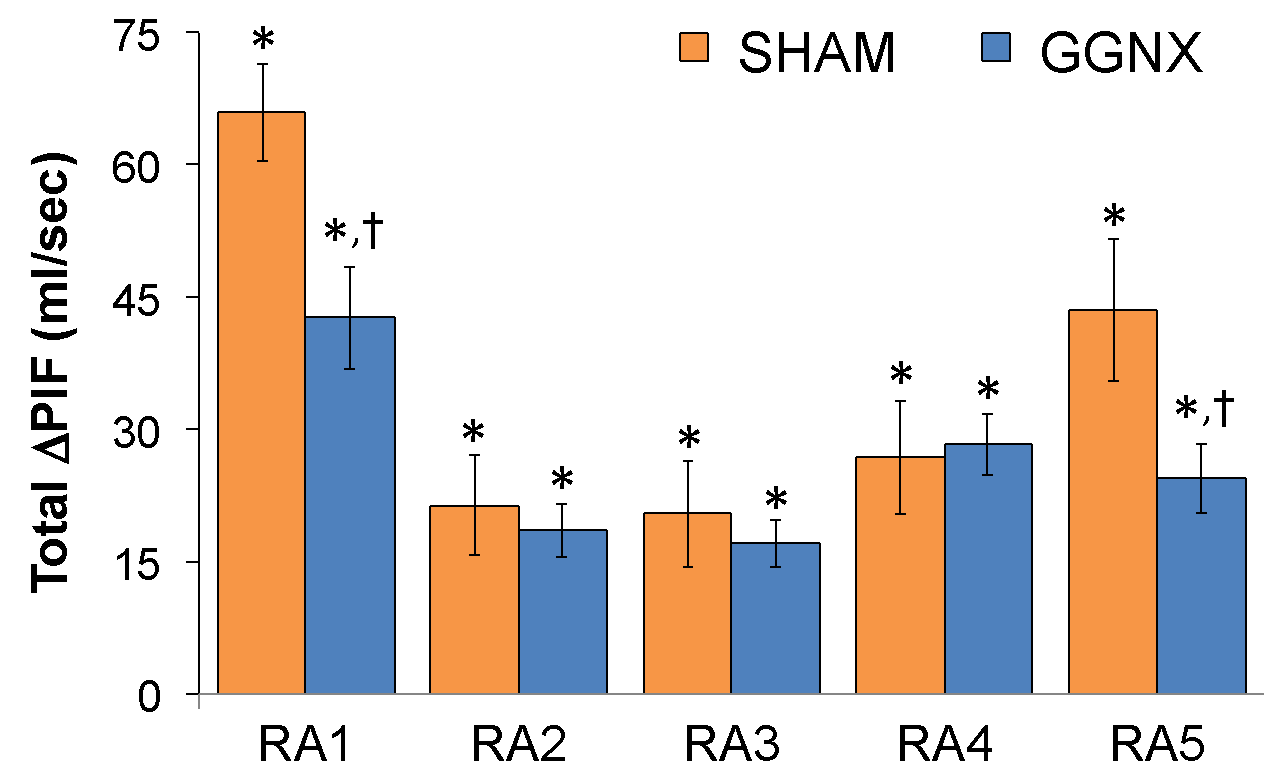


**Supplementary Figure S16. Panel A:** Arithmetic changes in Peak Inspiratory Flow (Peak Insp Flow, PIF) from Pre-values in sham-operated (SHAM) rats and in rats with bilateral ganglioglomerular nerve transection (GGNX) during the first 90 seconds upon return to room-air (RA1-RA5), following the 5 hypoxic (HX, 10% O_2_, 90% N_2_) gas challenges. **Panel B:** Total arithmetic changes in PIF (ΔPIF) during the first 90 sec of the return to room-air phases (RA1-RA5). **Panel C:** Total arithmetic changes in PIF (ΔPIF) during the first 5 min of the return to room-air phases (RA1-RA5). The SHAM group had 10 rats. The GGNX group had 12 rats. The data are presented as mean ± SEM. **p* < 0.05, significant response. ^†^*p* < 0.05, GGNX rats *versus* SHAM rats.

**Supplementary Figure S17**



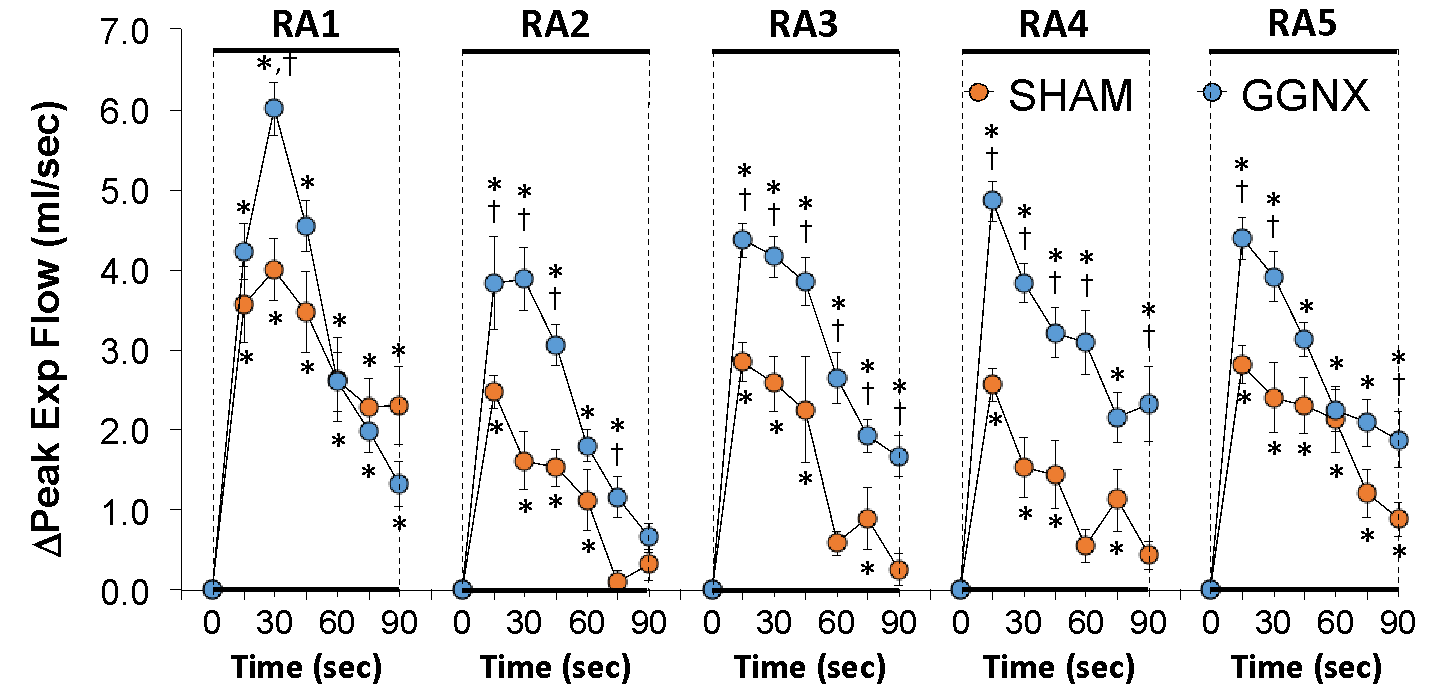


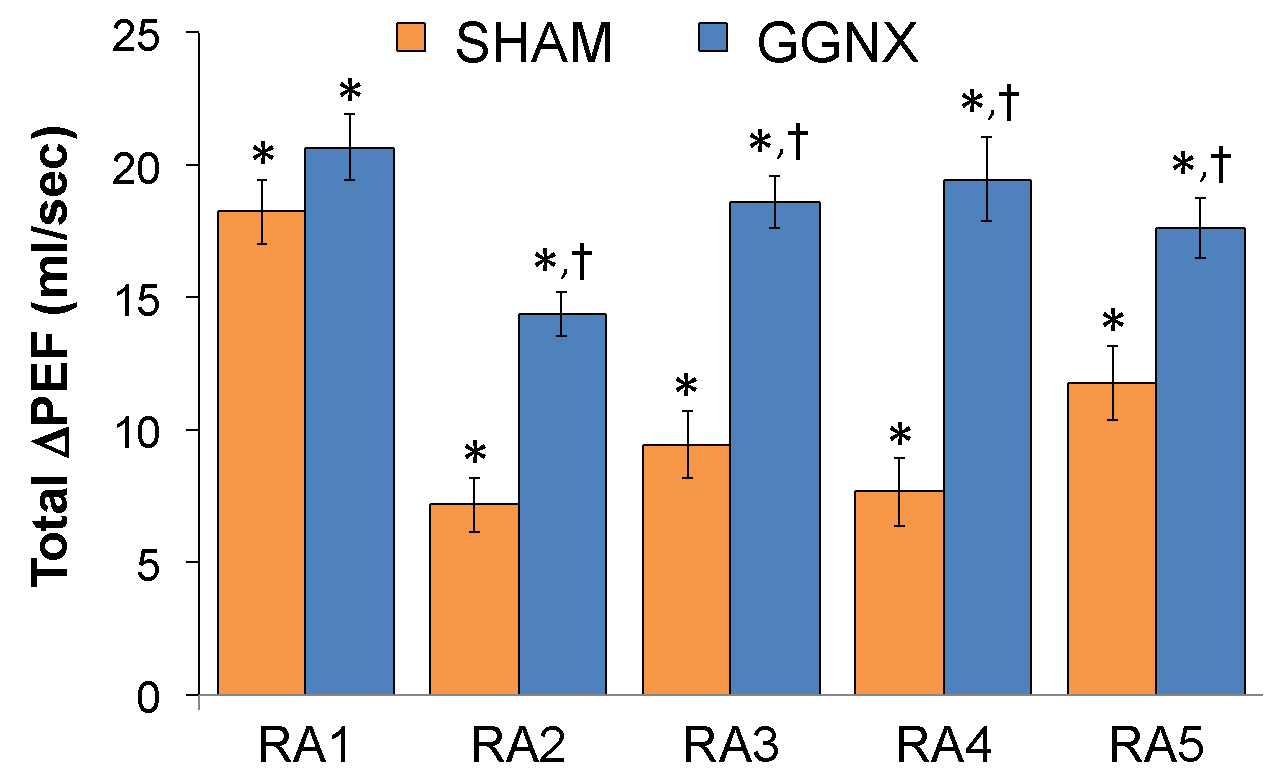

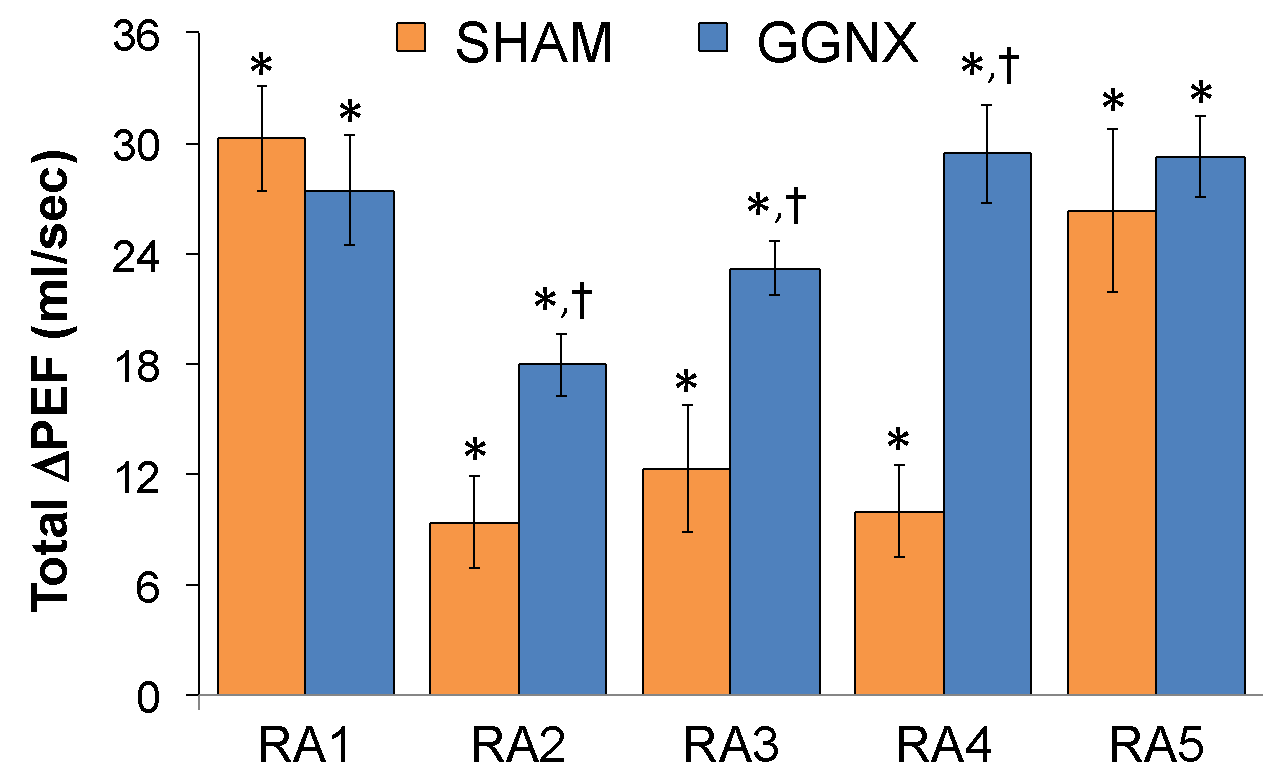


**Supplementary Figure S17. Panel A:** Arithmetic changes in Peak Expiratory Flow (Peak Exp Flow, PEF) from Pre-values in sham-operated (SHAM) rats and in rats with bilateral ganglioglomerular nerve transection (GGNX) during the first 90 seconds upon return to room-air (RA1-RA5), following the 5 hypoxic (HX, 10% O_2_, 90% N_2_) gas challenges. **Panel B:** Total arithmetic changes in PEF (ΔPEF) during the first 90 sec of the return to room-air phases (RA1-RA5). **Panel C:** Total arithmetic changes in PEF (ΔPEF) during the first 5 min of the return to room-air phases (RA1-RA5). The SHAM group had 10 rats. The GGNX group had 12 rats. The data are presented as mean ± SEM. * *p* < 0.05, significant response. ^†^*p* < 0.05, GGNX rats *versus* SHAM rats.

**Supplementary Figure S18**



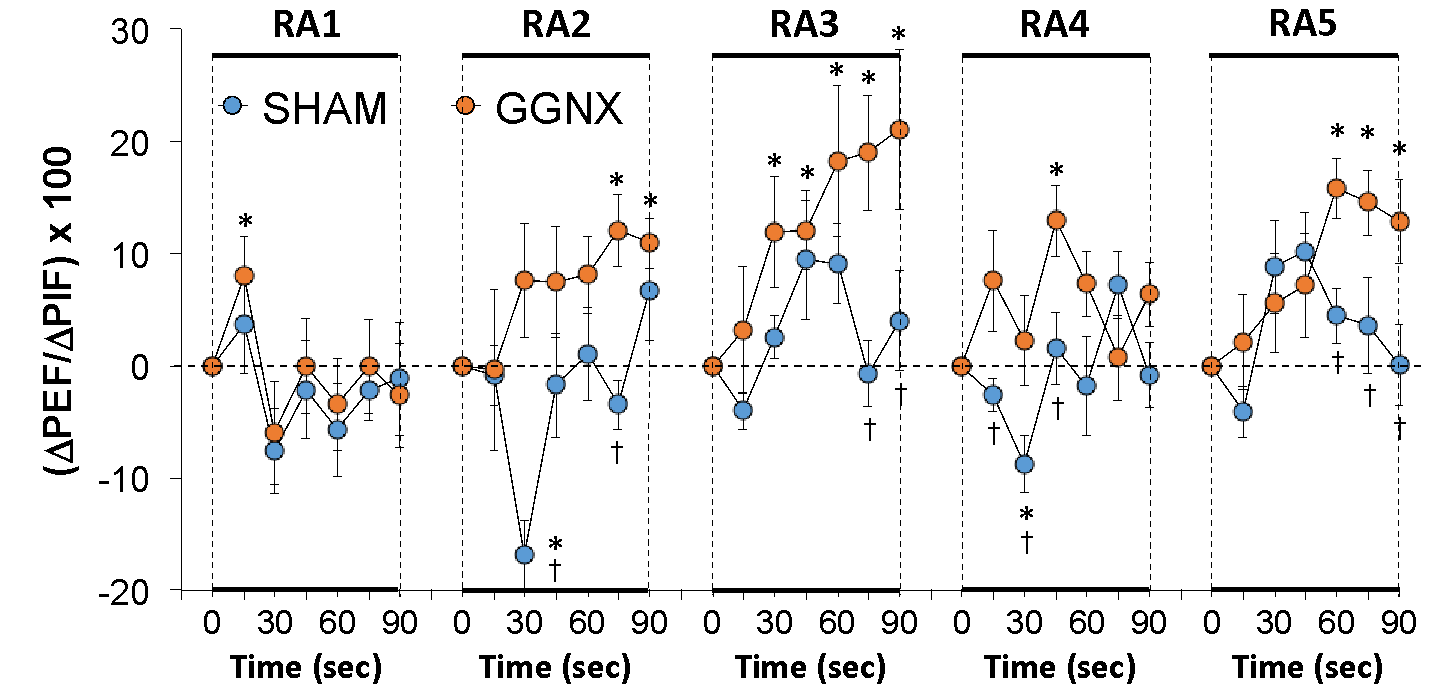


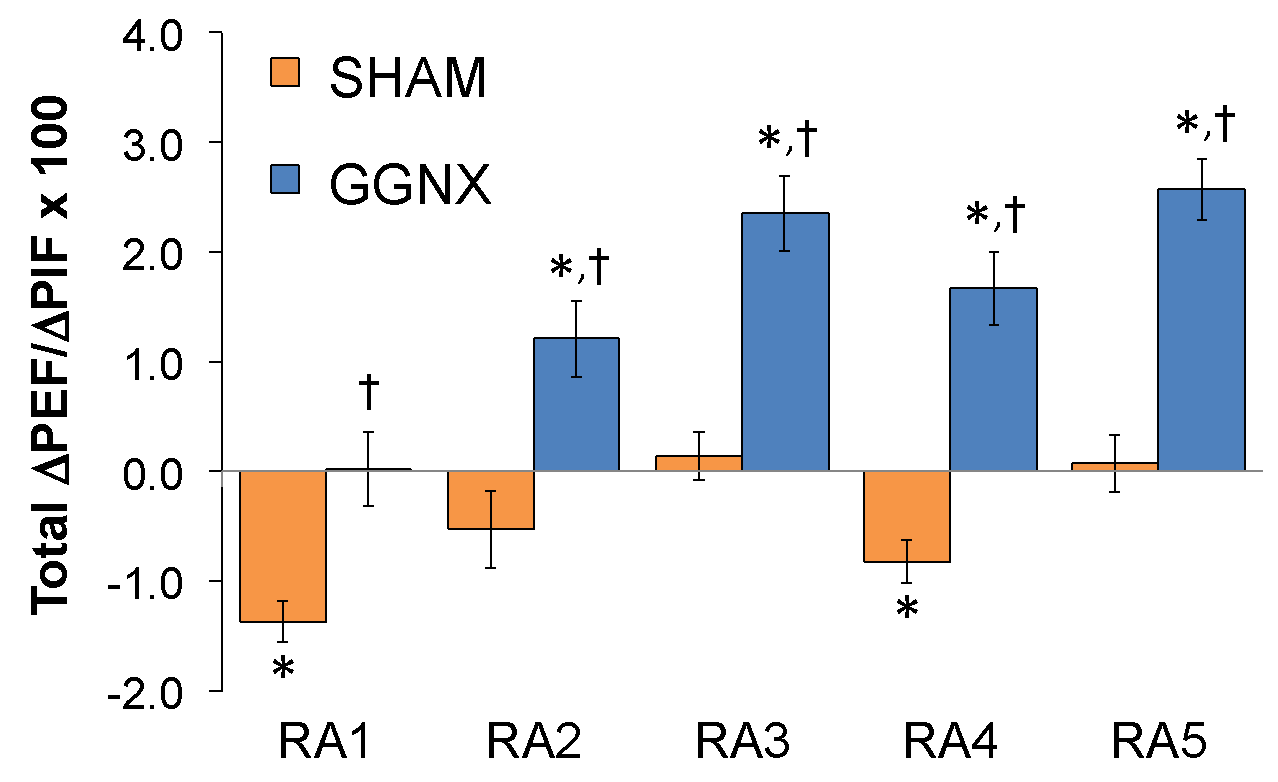


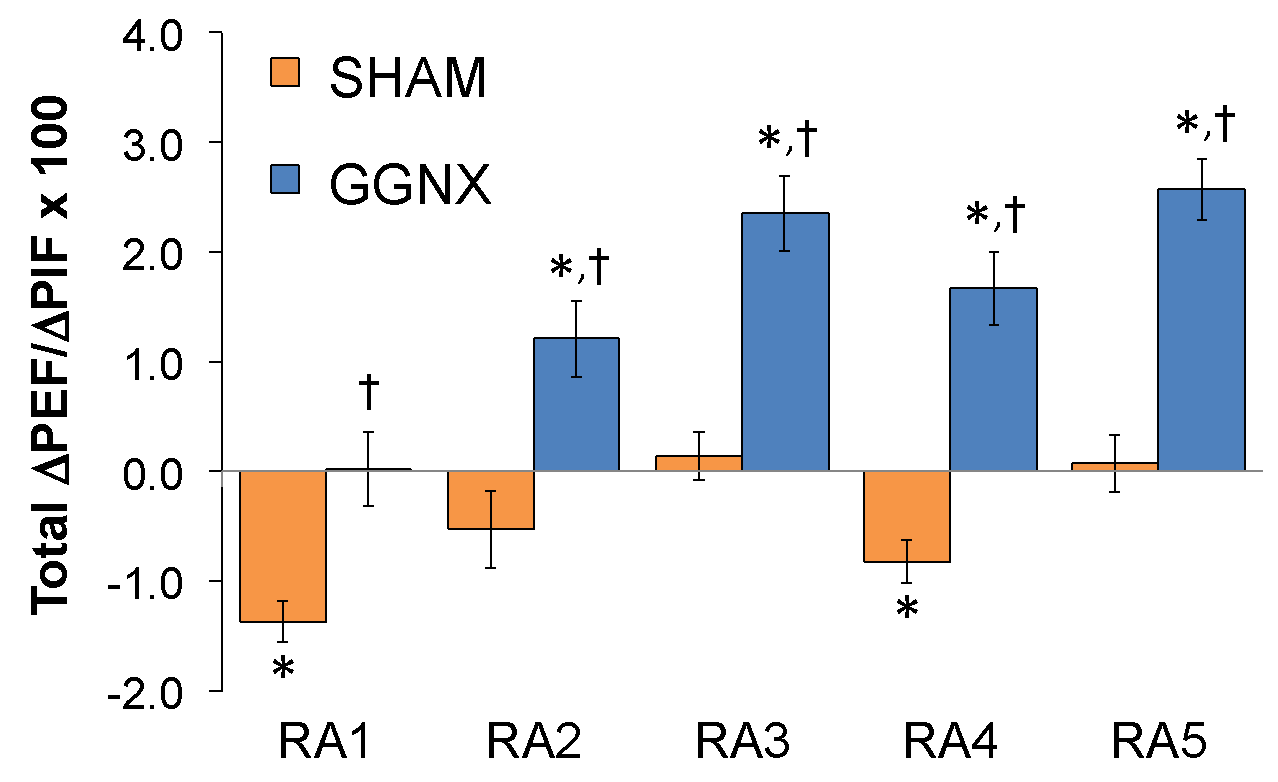

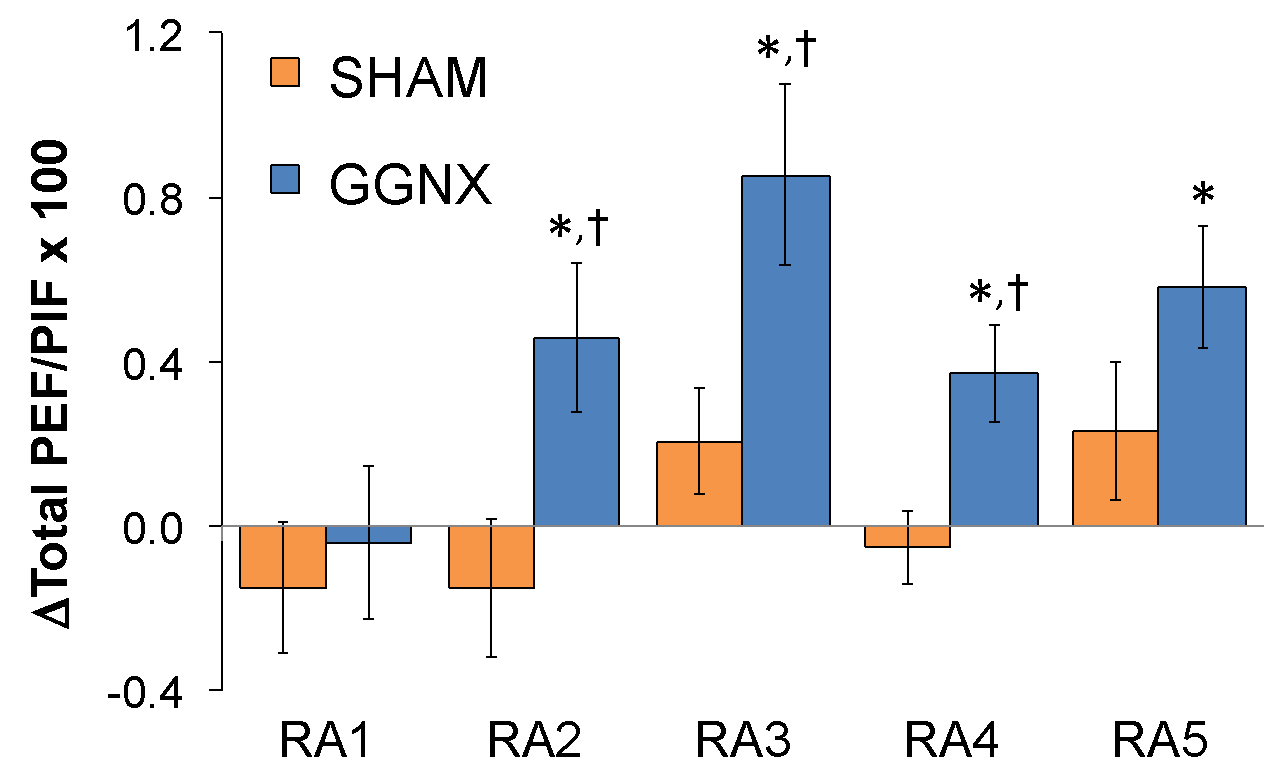


**Supplementary Figure S18. Panel A:** Arithmetic changes in Peak Expiratory Flow/Peak Inspiratory Flow (PEF/PIF) from Pre-values in sham-operated (SHAM) rats and in rats with bilateral ganglioglomerular nerve transection (GGNX) during the first 90 seconds upon return to room-air (RA1-RA5), following the 5 hypoxic (HX, 10% O_2_, 90% N_2_) gas challenges. **Panel B:** Total arithmetic changes in PEF/PIF (ΔPEF/(ΔPIF) during the first 90 sec of the return to room-air phases (RA1-RA5). **Panel C:** Total arithmetic changes in PEF/PIF (ΔPEF/(ΔPIF) during the first 5 min of the return to room-air phases (RA1-RA5). The SHAM group had 10 rats. The GGNX group had 12 rats. The data are presented as mean ± SEM. **p* < 0.05, significant response. ^†^*p* < 0.05, GGNX rats *versus* SHAM rats.

**Supplementary Figure S19**



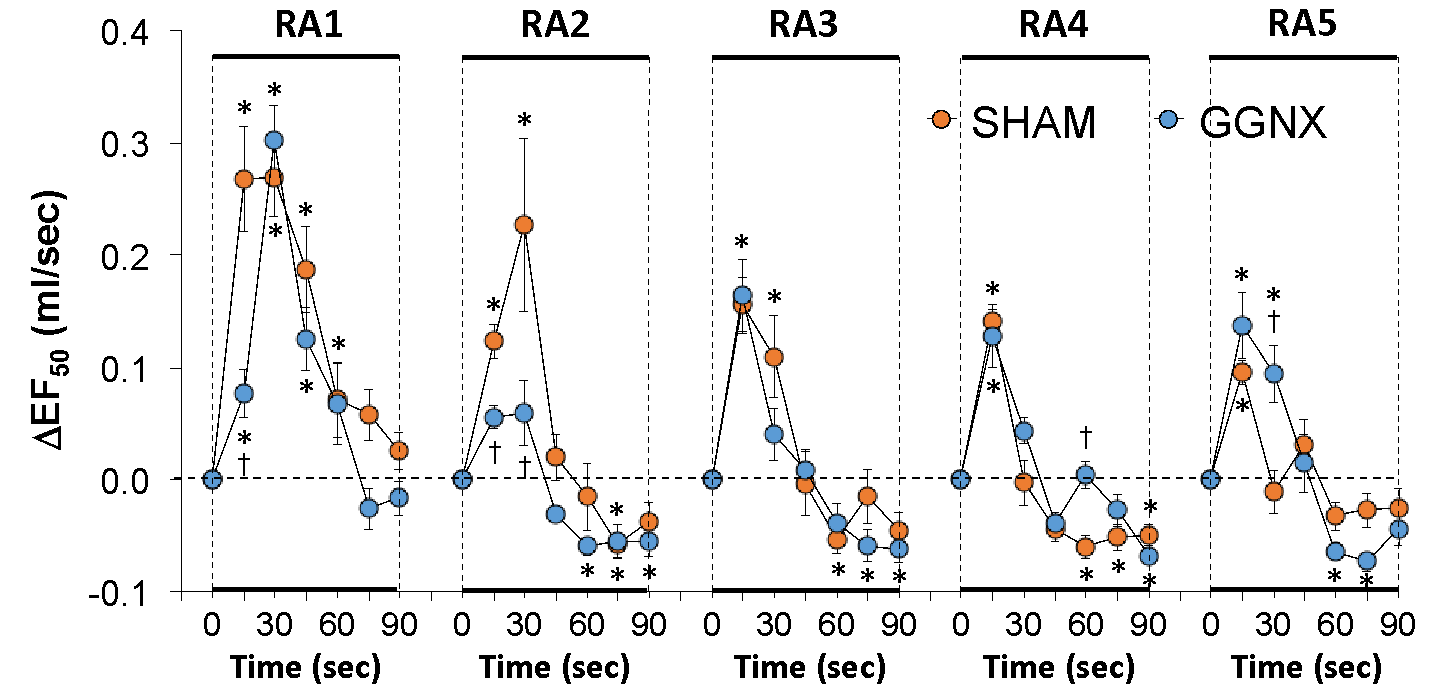


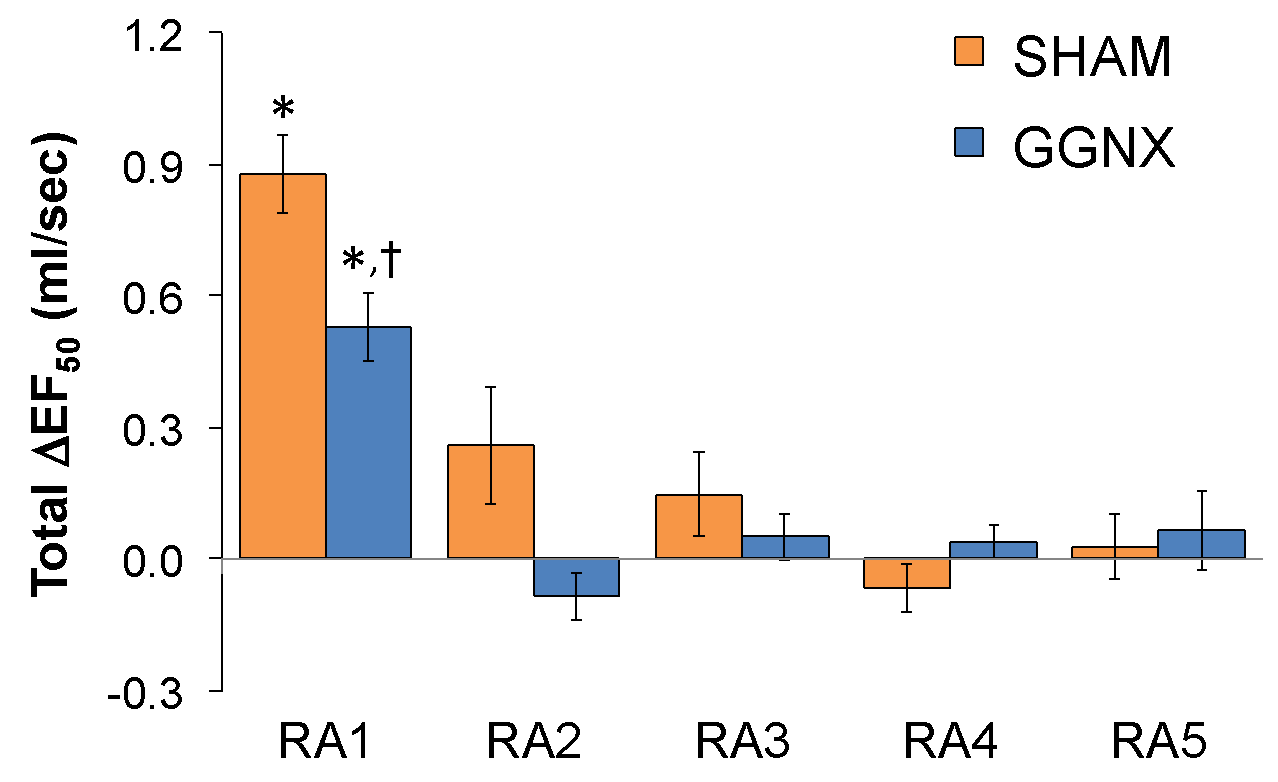


**Supplementary Figure S19. Panel A:** Arithmetic changes in Expiratory Flow at 50% expired tidal volume (EF_50_) from Pre-values in sham-operated (SHAM) rats and in rats with bilateral ganglioglomerular nerve transection (GGNX) during the first 90 seconds upon return to room-air (RA1-RA5), following the 5 hypoxic (HX, 10% O_2_, 90% N_2_) gas challenges. **Panel B:** Total arithmetic changes in EF_50_ (ΔEF_50_) during the first 90 sec of the return to room-air phases (RA1-RA5). **Panel C:** Total arithmetic changes in EF_50_ (ΔEF_50_) during the first 5 min of the return to room-air phases (RA1-RA5). The SHAM group had 10 rats. The GGNX group had 12 rats. The data are presented as mean ± SEM. **p* < 0.05, significant response. ^†^*p* < 0.05, GGNX rats *versus* SHAM rats.

**Supplementary Figure S20**

**Supplementary Figure S20. Panel A:** Arithmetic changes in Relaxation Time (RT) from Pre-values in sham-operated (SHAM) rats and in rats with bilateral ganglioglomerular nerve transection (GGNX) during the first 90 seconds upon return to room-air (RA1-RA5), following the 5 hypoxic (HX, 10% O_2_, 90% N_2_) gas challenges. **Panel B:** Total arithmetic changes in RT (ΔRT) during the first 90 sec of the return to room-air phases (RA1-RA5). **Panel C:** Total arithmetic changes in RT (ΔRT) during the first 5 min of the return to room-air phases (RA1-RA5). The SHAM group had 10 rats. The GGNX group had 12 rats. The data are presented as mean ± SEM. **p* < 0.05, significant response. ^†^*p* < 0.05, GGNX rats *versus* SHAM rats.

**Supplementary Figure S21**

**Supplementary Figure S21. Panel A:** Arithmetic changes in Apneic Pause from Pre-values in sham-operated (SHAM) rats and in rats with bilateral ganglioglomerular nerve transection (GGNX) during the first 90 seconds upon return to room-air (RA1-RA5), following the 5 hypoxic (HX, 10% O_2_, 90% N_2_) gas challenges. **Panel B:** Total arithmetic changes in Apneic Pause during the first 90 sec of the return to room-air phases (RA1-RA5). **Panel C:** Total arithmetic changes in Apneic Pause during the first 5 min of the return to room-air phases (RA1-RA5). The SHAM group had 10 rats. The GGNX group had 12 rats. The data are presented as mean ± SEM. **p* < 0.05, significant response.

**Supplementary Figure S22**

**Supplementary Figure S22. Panel A:** Arithmetic changes in Inspiratory Drive (Insp Drive) from Pre-values in sham-operated (SHAM) rats and in rats with bilateral ganglioglomerular nerve transection (GGNX) during the first 90 seconds upon return to room-air (RA1-RA5), following the 5 hypoxic (HX, 10% O_2_, 90% N_2_) gas challenges. **Panel B:** Total arithmetic changes in Inspiratory Drive (ΔInsp Drive) during the first 90 sec of the return to room-air phases (RA1-RA5). **Panel C:** Total arithmetic changes in Inspiratory Drive (ΔInsp Drive) during the first 5 min of the return to room-air phases (RA1-RA5). The SHAM group had 10 rats. The GGNX group had 12 rats. The data are presented as mean ± SEM. **p* < 0.05, significant response. ^†^*p* < 0.05, GGNX rats *versus* SHAM rats.

**Supplementary Figure S23**

**Supplementary Figure S23. Panel A:** Arithmetic changes in Expiratory Drive (Exp Drive) from Pre-values in sham-operated (SHAM) rats and in rats with bilateral ganglioglomerular nerve transection (GGNX) during the first 90 seconds upon return to room-air (RA1-RA5), following the 5 hypoxic (HX, 10% O_2_, 90% N_2_) gas challenges. **Panel B:** Total arithmetic changes in Exp Drive (ΔExp Drive) during the first 90 sec of the return to room-air phases (RA1-RA5). **Panel C:** Total arithmetic changes in Exp Drive (ΔExp Drive) during the first 5 min of the return to room-air phases (RA1-RA5). The SHAM group had 10 rats. The GGNX group had 12 rats. The data are presented as mean ± SEM. **p* < 0.05, significant response. ^†^*p* < 0.05, GGNX rats *versus* SHAM rats.

**Supplementary Figure S24**

**Total ΔNEBI/ΔFreq x 100**

**Total ΔNEBI/ΔFreq x 100**

**Supplementary Figure S24. Panel A:** Arithmetic changes in Non-Eupneic Breathing Index/Frequency of breathing index (NEBI/Freq) from Pre-values in sham-operated (SHAM) rats and in rats with bilateral ganglioglomerular nerve transection (GGNX) during the first 90 seconds upon return to room-air (RA1-RA5), following the 5 hypoxic (HX, 10% O_2_, 90% N_2_) gas challenges. **Panel B:** Total arithmetic changes in NEBI/Freq during the first 90 sec of the return to room-air phases (RA1-RA5). **Panel C:** Total arithmetic changes in NEBI/Freq during the first 5 min of the return to room-air phases (RA1-RA5). The SHAM group had 10 rats. The GGNX group had 12 rats. The data are presented as mean ± SEM. **p* < 0.05, significant response. ^†^*p* < 0.05, GGNX rats *versus* SHAM rats.

**Supplementary Table S3**

Qualitative status of resting (Pre-challenge) ventilatory parameters in GGNX and CSNX rats compared to their respective sham-operated (SHAM) controls

| **Parameter** | **GGNX rats** | **CSNX rats** |
| --- | --- | --- |
| Frequency of breathing, breaths/min | ↓ | ↓ |
| Tidal Volume (TV), ml | = | = |
| Minute Ventilation, ml/min | = | ↓ |
| Inspiratory time (Ti), sec | = | = |
| Expiratory time (Te), sec | ↑ | ↑ |
| Expiratory time/Inspiratory time | = | = |
| End inspiratory pause, msec | = | = |
| End expiratory pause, msec | ↑ | = |
| Peak inspiratory flow (PIF), ml/sec | = | = |
| Peak expiratory flow (PEF), ml/sec | = | ↓ |
| PEF/PIF | ↑ | = |
| Expiratory flow at 50% expired TV (EF_50_), ml/sec | = | = |
| Relaxation time (RT), sec | = | = |
| Apneic pause, (Te/RT)-1 | = | = |
| Inspiratory Drive (TV/Ti, ml/sec | = | ↓ |
| Expiratory Drive (TV/Te), ml/sec | = | = |
| Non-eupneic breathing index (NEBI), % | ↓ | = |
| NEBI/frequency, %/(breaths/min) | = | ↑ |

GGNX, bilateral ganglioglomerular nerve transection. CSNX, bilateral carotid sinus nerve transection. The original Freq, TV and MV data for the CSNX rats is from Getsy et al (2020) and the original data for the variables described for the CSNX rats is unpublished.

**Supplementary Table S4**

Qualitative status of ventilatory responses during HX1-HX5 in GGNX rats compared to SHAM rats

|  |  | **Initial 90 sec of HX challenge** | | | | |  | **Full 5 min of HX challenge** | | | | |
| --- | --- | --- | --- | --- | --- | --- | --- | --- | --- | --- | --- | --- |
| **Parameter** |  | **HX1** | **HX2** | **HX3** | **HX4** | **HX5** |  | **HX1** | **HX2** | **HX3** | **HX4** | **HX5** |
| Increase in Frequency of Breathing (Freq) |  | = | ↓ | ↓ | ↓ | ↓ |  | = | ↓ | ↓ | ↓ | ↓ |
| Increase in Tidal Volume (TV) |  | ↓ | ↓ | ↓ | ↓ | ↓ |  | = | = | = | = | = |
| Increase in Minute Ventilation |  | = | ↓ | ↓ | ↓ | ↓ |  | = | = | = | = | = |
| Decrease in Inspiratory Time (Ti) |  | = | ↓ | ↓ | = | = |  | = | = | ↓ | = | = |
| Decrease in Expiratory Time (Te) |  | = | = | = | = | = |  | = | = | = | = | = |
| Change in Te/Ti |  | = | ↑ | = | = | = |  | = | = | = | = | = |
| Decrease in End Inspiratory Pause |  | = | = | = | = | = |  | ↑ | = | ↑ | ↑ | = |
| Increase in End Expiratory Pause |  | ↑ | ↑ | = | = | = |  | ↑ | ↑ | ↑ | ↑ | ↑ |
| Increase in Peak Inspiratory Flow (PIF) |  | = | ↓ | ↓ | ↓ | ↓ |  | = | = | = | = | = |
| Increase in Peak Expiratory Flow (PIF) |  | = | ↓ | ↓ | ↓ | ↓ |  | = | = | ↑ | ↑ | ↑ |
| Increase in PEF/PIF except for a decrease (d) during HX1 |  | ↑ | ↑ | = | = | = |  | = | ↑ | ↑ | ↑ | ↑ |
| Increase in Expiratory Flow at 50% expired TV (EF_50_) |  | = | ↓ | ↓ | ↓ | ↓ |  | = | ↓ | ↓ | ↓ | ↓ |
| Decrease in Relaxation Time |  | = | = | = | = | = |  | = | = | = | = | = |
| Changes in Apneic Pause |  | = | = | = | = | = |  | = | = | = | = | = |
| Increase in Inspiratory Drive (TV/Ti) |  | ↓ | ↓ | ↓ | ↓ | ↓ |  | = | = | = | = | = |
| Increase in Expiratory Drive (TV/Te) |  | ↓ | ↓ | ↓ | ↓ | ↓ |  | = | = | = | = | = |
| Increase in Non-Eupneic Breathing Index (NEBI) |  | = | = | = | = | = |  | ↑ | ↑ | ↑ | ↑ | ↑ |
| Increase in NEBI/Freq – initial 90 sec |  | = | = | ↑ | ↑ | ↑ |  |  |  |  |  |  |
| Decrease in NEBI/Freq – total 5 min – reversed to increase |  |  |  |  |  |  |  | ↑ | ↑ | ↑ | ↑ | ↑ |

HX1-HX5, hypoxic gas challenges 1-5. GGNX, rats with bilateral transection of the ganglioglomerular nerves; SHAM, rats with sham-operated ganglioglomerular nerves.

**Supplementary Table S5**

Qualitative status of ventilatory responses during RA1-RA5 in GGNX rats compared to SHAM rats

|  |  | **Total of 90 sec of RA phase** | | | | |  | **Total of 5 min of RA phase** | | | | |
| --- | --- | --- | --- | --- | --- | --- | --- | --- | --- | --- | --- | --- |
| **Parameter** |  | **RA1** | **RA2** | **RA3** | **RA4** | **RA5** |  | **RA1** | **RA2** | **RA3** | **RA4** | **RA5** |
| Increase in Freq, reversed to significant decrease (d) |  | = | ↓ | ↓ | ↓ | ↓ |  | ↓ | d | d | = | d |
| Decreases in Tidal Volume (TV), reversed to an increase (i) |  | i | i | i | i | i |  | = | ↓ | i | i | = |
| Changes in Minute Ventilation, reversed in GGNX rats (r) |  | = | = | = | ↑ | = |  | ↓ | = | = | r | r |
| Decreases in Inspiratory Time (Ti) |  | = | = | = | = | = |  | ↓ | = | = | = | = |
| Decreases in Te (RA1), increase in Te (RA2-RA4), reversed (r) |  | ↓ | ↑ | = | = | = |  | r | ↑ | = | = | ↑ |
| Increases in Te/Ti |  | ↑ | = | = | = | = |  | = | = | = | ↑ | = |
| Decreases in End Inspiratory Pause |  | = | ↓ | ↓ | ↓ | = |  | ↓ | ↓ | ↓ | ↓ | ↓ |
| Increases in End Expiratory Pause |  | ↑ | ↑ | ↑ | ↑ | ↑ |  | ↑ | ↑ | ↑ | ↑ | ↑ |
| Increases in Peak Inspiratory Flow (PIF) |  | = | = | = | ↑ | = |  | ↓ | = | = | = | ↓ |
| Increases in Peak Expiratory Flow (PEF) |  | = | ↑ | ↑ | ↑ | ↑ |  | = | ↑ | ↑ | ↑ | = |
| Changes in PEF/PIF, reversed (r) |  | = | ↑ | ↑ | ↑ | = |  | r | r | r | r | r |
| Changes in Expiratory Flow at 50% expired TV (EF_50_) |  | ↓ | = | = | = | = |  | ↓ | ↑ | ↑ | = | ↑ |
| Changes in Relaxation Time, reversed in GGNX rats (r) |  | ↓ | = | = | = | = |  | r | = | = | = | = |
| Increases in Apneic Pause |  | = | = | = | = | = |  | = | = | = | = | = |
| Increase in Inspiratory Drive (TV/Ti) |  | = | = | ↑ | ↑ | = |  | = | = | ↑ | ↑ | ↑ |
| Changes in Expiratory Drive (TV/Te) |  | ↓ | = | = | = | = |  | ↓ | = | = | = | = |
| Increases in Non-Eupneic Breathing Index (NEBI) |  | ↓ | ↓ | ↓ | ↓ | ↓ |  | ↓ | ↓ | ↓ | ↓ | ↓ |
| Increases in NEBI/Freq |  | ↓ | ↓ | ↓ | ↓ | ↓ |  | ↓ | ↓ | ↓ | ↓ | ↓ |

RA1-RA5, return to room-air responses 1-5. GGNX rat, rats with bilateral transection of the ganglioglomerular nerves; SHAM rats, rats with sham-operated ganglioglomerular nerves.
